# Down-Regulation of SIX3 is Associated with Clinical Outcome in Lung Adenocarcinoma

**DOI:** 10.1371/journal.pone.0071816

**Published:** 2013-08-16

**Authors:** Min-Li Mo, Junichi Okamoto, Zhao Chen, Tomomi Hirata, Iwao Mikami, Geneviève Bosco-Clément, Hui Li, Hai-Meng Zhou, David M. Jablons, Biao He

**Affiliations:** 1 School of Life Sciences, Tsinghua University, Beijing, China; 2 Thoracic Oncology Program, Department of Surgery, Helen Diller Family Comprehensive Cancer Center, University of California, San Francisco, California, United States of America; 3 Department of Surgery, Division of Thoracic Surgery, Nippon Medical School, Tokyo, Japan; 4 Zhejiang Provincial Key Laboratory of Applied Enzymology, Yangtze Delta Region Institute of Tsinghua University, Jiaxing, China; The University of Tennessee Health Science Center, United States of America

## Abstract

**Background:**

Lung cancer is a common cancer and the leading cause of cancer-related death worldwide. SIX3 is a human homologue of the highly conserved sine oculis gene family essential during embryonic development in vertebrates, and encodes a homeo-domain containing transcription factor. Little is known about the role of SIX3 in human tumorigenesis. This study is to assess the expression/function of SIX3 and the significance of SIX3 as a prognostic biomarker in lung adenocarcinoma.

**Methods:**

Quantitative real-time RT-PCR was used to analyze SIX3 mRNA expression and quantitative methylation specific PCR (MSP) was used to examine promoter methylation. MTS and colony formation assays were performed to examine cell proliferation. Wound healing assays were used to assess cell migration, and microarrays were utilized to examine genes regulated by SIX3 in lung cancer cells. Association of SIX3 expression levels with clinical outcomes of patients with lung adenocarcinoma was evaluated using the Kaplan-Meier method and a multivariate Cox proportional hazards regression model.

**Results:**

SIX3 was down-regulated in lung adenocarcinoma tissues compared to their matched adjacent normal tissues, and this down-regulation was associated with methylation of the SIX3 promoter. SIX3 was also methylation-silenced in lung cancer cell lines. Restoration of SIX3 in lung cancer cells lacking endogenous SIX3 suppressed cell proliferation and migration, and downregulated a number of genes involved in proliferation and metastasis such as S100P, TGFB3, GINS3 and BAG1. Moreover, SIX3 mRNA expression was associated with significantly improved overall survival (OS) and progression-free survival (PFS) in adenocarcinoma patients and patients with bronchioloalveolar carcinoma (BAC) features.

**Conclusions:**

SIX3 may play an important role as a novel suppressor in human lung cancer. SIX3 has potential as a novel prognostic biomarker for patients with lung adenocarcinomas.

## Introduction

Lung cancer is one of the most common cancers and the leading cause of cancer-related death worldwide [1]. Non-small-cell lung cancer (NSCLC) constitutes ∼80% of all lung cancers [2]. Despite recent development of cancer treatments based on TNM staging, prognosis of lung cancer patients has limited improvement. Survival rate of all stages in NSCLC is about 15% [3], and even for stage I patients, the survival rate is only approximately 60–80% [4]. Recently, many efforts have been made in searching and validating novel and more meaningful molecular prognostic biomarkers using expression profiling based prognostic models[5]–[9].

Cancer specific methylation of genes that suppress uncontrolled cell proliferation or promote genome stability is one of the key steps during tumor development [10]. SIX3 belongs to the sine oculis family and is a highly conserved gene that is required for the initiation of eye development in vertebrates. SIX family genes code for transcription factors that are characterized by a homeo domain and a SIX domain [11]. Little is known about the role of SIX3 during human tumorigenesis. In this study we intend to investigate the expression and function of SIX3 in human lung cancer, and potential of SIX3 expression as a prognostic biomarker for patients with lung adenocarcinoma.

## Materials and Methods

### Ethics Statement

Investigation has been conducted in accordance with the ethical standards and according to the Declaration of Helsinki, as well as national and international guidelines, and has been approved by the Committee on Human Research at the University of California, San Francisco.

### Patients and Tissue Samples

From July 1999 to August 2006, consecutive patients with NSCLC who underwent surgery at University of California, San Francisco were studied. TNM staging designations were made according to the postsurgical pathologic international staging system from World Health Organization classification. The lymph node status was pathologically evaluated using resected specimens. A total of 160 patients were investigated (Table 1). The patients’ clinical records and histopathologic diagnosis were documented, including follow-up data as of May 10, 2007. The median follow-up period for all patients was 42.8±2.1 months. Fresh lung cancer tissue samples were collected from patients undergoing resection of their tumors. These tissue samples were snap-frozen in liquid nitrogen immediately after resection and kept at −170°C before use. Written consent was obtained from the participants.

**Table 1:** SIX3 expression and clinical characteristics in cancer patients.

Characteristics		NSCLC (N = 160)			
		Number	%	SIX3 Expression	p value
Age (years)					
Median	67.4±0.8				
Whole		160	100	21.72±5.64	
Young (<50)		9	5.6	2.83±1.54	
Middle (>50, <75)		107	66.9	21.5±6.71	
Old (>75)		44	27.5	26.13±12.47	N.S.
Sex					
Male		63	39.4	10.02±3.46	
Female		97	60.6	29.32±9.00	0.04
Race					
Caucasian		123	76.8	22.23±6.79	
Asian		22	13.8	26.31±15.51	
Others		15	9.4	11.36±5.33	N.S.
Smoking Status					
Never		32	20	18.27±9.84	
Past		79	49.4	24.92±8.92	
Current		44	27.5	20.09±10.81	
Unknown		5	3.1	7.59±3.60	N.S.
Smoking/Sex					
Male, Smoker		55	34.4	9.92±3.77	
Male, Non-Smoker		7	4.4	11.72±10.36	
Female, Smoker		68	42.5	33.93±11.96	
Female, non-Smoker		25	15.6	20.10±12.33	
Unknown		5	3.1	7.59±3.60	N.S.
Tumor Size (cm)					
Median	3.5±0.2				
3 cm or less		85	53.1	23.59±7.47	
over 3 cm		74	46.3	19.77±8.72	0.01
Pathological Stage					
I		99	61.9	21.42±7.17	
II		21	13.1	33.89±22.37	
III		30	18.7	20.47±11.45	
IV		10	6.3	6.16±3.00	N.S.
Histology					
Adenocarcinoma		81	50.6	16.09±6.86	
BAC		64	40	33.57±10.99	
Squamous		15	9.4	1.57±0.38	N.S.
*ECOG PS					
0		86	53.8	26.83±9.01	
1		32	20	24.07±14.10	
2		6	3.7	0.94±0.27	
Unknown		36	22.5	10.89±2.83	0.01
Operation Procedure					
Wedge Resection		14	8.8	9.28±2.91	
Segmentectomy		5	3.1	88.61±87.43	
Lobectomy		126	78.8	22.20±6.29	
bilobectomy		9	5.6	9.70±8.04	
Pnumonectomy		6	3.7	3.04±1.86	N.S.
Vital Status					
Alive		100	62.5	28.49±8.29	
Dead		60	37.5	10.43±5.74	0.004
Recurrence					
Positive		65	40.6	11.67±5.41	
Negative		77	48.1	33.95±10.66	0.02
Follow-up (months)					
Median	42.8±2.1				

* ECOG PS: Eastern Cooperative Oncology Group Performance Status.

### RNA Extraction

Total RNA from cell lines was isolated using an extraction kit (RNeasy Mini kit; Qiagen). Total RNA from fresh tumor samples was extracted with TRIzol LS (Life Technologies).

### Semi-quantitative and Quantitative Real-time RT-PCR

Semi-quantitative RT-PCR was performed in a GeneAmp PCR system 9700 (Applied Biosystems (ABI)) using SuperScript II one-step RT-PCR with Platinum Taq kit (Life Technologies) according to the manufacturer’s protocol. Primers for RT-PCR were obtained from Operon. Total RNA of three normal human adult lung tissues was obtained from BioChain. 500 ng of total RNA was used for each reaction.

For real-time RT-PCR, first-strand cDNA was synthesized from total RNA using iScript cDNA synthesis kit (Bio-Rad) according to the manufacturer’s instructions. Transcript analysis was done by real-time reverse transcription-PCR using the TaqMan assay. Hybridization probes and primers were purchased from ABI. All samples were run simultaneously in triplicate in a 7900 real-time PCR System. To calculate SIX3 expression levels, each Ct value was normalized to its housekeeping gene GAPDH level (dCt), and expression level was calculated by 2^−dCt^×100 method.

### Cell Culture and 5-Aza-2′-deoxycytidine (DAC) Treatment

Human lung cancer cell lines A549, H1703, H838, H1650, H1975, H460, A427, H358, H1299, H522, H322, and H441 were purchased from American Type Culture Collection (ATCC). Genomics, Proteomics and immunology technologies are used to characterize cell lines at ATCC. Experiments were performed within 3 months after resuscitation of each cell line. They were cultured in RPMI 1640 with 10% fetal bovine serum, penicillin (100 IU/ml)/streptomycin (100 µg/ml) at 37°C in a humidified 5% CO_2_ incubator.

For DAC treatment, 1×10^5^ cells were seeded into 6-well culture plates in regular medium for 24 hours. Then fresh culture medium with 5 µM DAC (Sigma) was used to replace regular medium to culture the cells, and cells were harvested after 72 hours. Total RNA was extracted using Qiagen RNeasy Mini kit for semi-quantitative RT-PCR analysis.

### Quantitative Methylation-specific PCR (qMSP)

qMSP was modified from the previous study[12]–[16]. Genomic DNA from cell lines and tissue samples were extracted with a DNeasy tissue kit (Qiagen), according to the manufacturer’s instruction. Primers and probes were designed using Primer Express® Software and Methyl Primer Express® Software v1.0 (ABI). Bisulfite modification of genomic DNA was performed with a methylation kit (EZ DNA Methylation-Gold Kit; Zymo Research). In addition, specific primers and probe were designed to amplify an internal reference gene, ACTB [16]. They were located in areas without CpG nucleotides, thus amplifying the ACTB gene independently from methylation status of CpG nucleotides. PCR was performed in the same plates for both genes. To determine the relative levels of methylated promoter DNA in each sample, the values of the gene of interest were compared with the values of the internal reference gene to obtain a ratio that was then multiplied by 100 to give a percentage value (gene of interest/reference gene×100). The primer sequences are: for SIX3 (113 - bp amplicon; position 1020–1171; GenBank accession # NC_000010), Forward, 5′-CGTTTTATATTTTTGGCGAGTAGC-3′, Reverse, 5′-ACTCCGCCAACACCG-3′, and TaqMan Probe, FAM5′- CGGCGGCGGCGCGGGAGGCGG-3′TAMRA; For ACTB (133 - bp amplicon; position 390–522; GenBank accession # Y00474), Forward, 5′-TGGTGATGGAGGAGGTTTAGTAAGT-3′, Reverse, 5′-AACCAATAAAACCTACTCCTCCCTTAA-3′, and TaqMan Probe, FAM5′-ACCACCACCCAACACACAATAACAAACACA-3′TAMRA.

Fluorogenic PCR was carried out in a reaction volume of 25 µl. Each PCR reaction mixture consisted of 500 nM of each primer, 200 nM probe, and TaqMan Universal PCR Master MIX (1×). Thermal cycling was initiated with a first denaturation step of 95°C for 10 min, followed by 45 cycles of 95°C for 15 s and 58°C for 1 min. Amplifications were carried out in 96-well plates in a 7300 Sequence detector (ABI). All samples were run in triplicate. Each plate included multiple water blanks, a negative control.

### Immunohistochemistry (IHC)

IHC staining of SIX3 was performed using a rabbit anti-SIX3 polyclonal antibody (Abcam) at 1∶500 dilution. Five-micron tissue slides from tumor and adjacent normal lung tissue were de-paraffinized using xylene. Heat-mediated antigen retrieval was performed using citrate buffer (BioGenex Laboratories). Antibody staining was visualized with DAB (Histostain Plus Broad Spectrum, Life Technologies) and hematoxylin counterstain (Fisher Scientific). Representative 40× fields were examined for positive nuclear staining and the mean number of positive cells in each tumor and matched normal tissue sample was compared using a 2-tailed paired *t* test.

### Transfection and RNA Interference

pcDNA 3.1/SIX3 mammalian expression-vector was subcloned from pCMV6-XL5/SIX3 vector (Origene). SIX3 shRNAs and control (non-silencing) shRNA (all are in pRFP-C-RS vector) were purchased from Origene. The targeted SIX3 sequences are: 5′- GGTAACCTCCAGCGACTCGGAATGTGATG-3′ (shRNA-1) and 5′- ACACAAGTAGGCAACTGGTTTAAGAACCG-3′ (shRNA-2). Cell lines were plated in six-well plates with fresh media without antibiotics for 24 hours before transfection. Transfection was performed using Lipofectamine2000 (Life Technologies) per manufacturer’s protocol. Transfected cells were re-plated in 10 cm dishes for selection with G418 (500 µg/ml; Life Technologies). Stable transfectants were maintained in regular medium with G418 (300 µg/ml) for further analysis.

### Proliferation Assays

Stably transfected cells were plated in 96-well plates at a density of 500–1000 cells/well in 100 µl of G418 culture medium. Medium was changed every day. Cell viability was evaluated in triplicate by MTS assay (CellTiter 96 Aqueous kit; Promega) per manufacturer’s protocol. For the colony formation assay, 500 individual cells of the stable lines were seeded in 10 cm dishes and cultured for 10 days. Colonies were then fixed by 10% formalin, stained with 0.5% crystal violet and counted.

### Microarray

Lung cancer cell line H1299 stably transfected with SIX3 and empty vector control was used on microarray for global gene expression analysis. Total RNA quality was assessed using a Pico Chip on an Agilent 2100 Bioanalyzer (Agilent). RNA was amplified and labeled with Cy3-CTP or Cy5-CTP using the Agilent low RNA input fluorescent linear amplification kits following the manufacturer’s protocol. Labeled cRNA was assessed using the Nandrop ND-100, and equal amounts of Cy3 and Cy5 labeled target were hybridized to Agilent whole human genome 44K Ink-jet arrays. Hybridization samples were randomized on the 3×44K format to correct any batch bias. Hybridizations were performed for 14 hours according to the manufacturer’s protocol. Arrays were scanned using the Agilent microarray scanner and raw signal intensities were extracted with Feature Extraction v9.5 software (Agilent).

Differential expression (DE) analysis was then performed on genes in H1299 cells stably transfected with SIX3 versus empty vector control. Raw log-intensities were normalized using *quantile* normalization method that is proposed by Bolstad et al [17]. No background subtraction was performed, and the median feature pixel intensity was used as the raw signal before normalization. A linear model was used to estimate effect of interest: fold changes of SIX3 vs control. Moderated t-statistic, B statistic, false discovery rate and p-value for each gene were obtained. Adjusted p-values were produced by the method proposed by Holm [18]. All procedures were carried out using functions in the R package *limma in Bioconductor* [19], [20]. DE declarations are summarized in Table S1.

### Statistical Analysis

The Kaplan-Meier method was used to estimate overall survival and progression-free survival. Differences in survival between low-risk group (high level of SIX3 expression) and high-risk group (low level of SIX3 expression) were analyzed by log-rank test. Optimal cut-off points for partitioning into high/low risk survival subgroups based on threshold SIX3 mRNA expression at the cut-off point were determined using survival trees. Assessment of the predictive reproducibility of the partition made recourse to cross-validation [21]. Cross-validation is operationally equivalent to having a training and validation cohort but uses data more efficiently by using repeated data-splitting [22]. Because cross-validation avoids model over-fitting and generates more accurate estimates of cut-off points [22], it is widely employed to generate prognostic marker models [23]–[26]. Multivariate Cox proportional hazards regression analysis was used to assess effect of SIX3 expression on survival. Hazard ratios (HR) and 95% confidence interval (CI) were calculated from Cox regression model. The associations between gene expression and discrete clinical categories were analyzed by the t-test, ANOVA with Bonferrini/Dunn test, Mann-Whitney’s U-test for variables with two-categories, and the Kruskal-Wallis test for variables with more than two categories. All reported p values were two-sided. Statistical significance was defined as p<0.05.

## Results

### SIX3 Expression was Downregulated by Methylation in NSCLC

**Figure 1:**
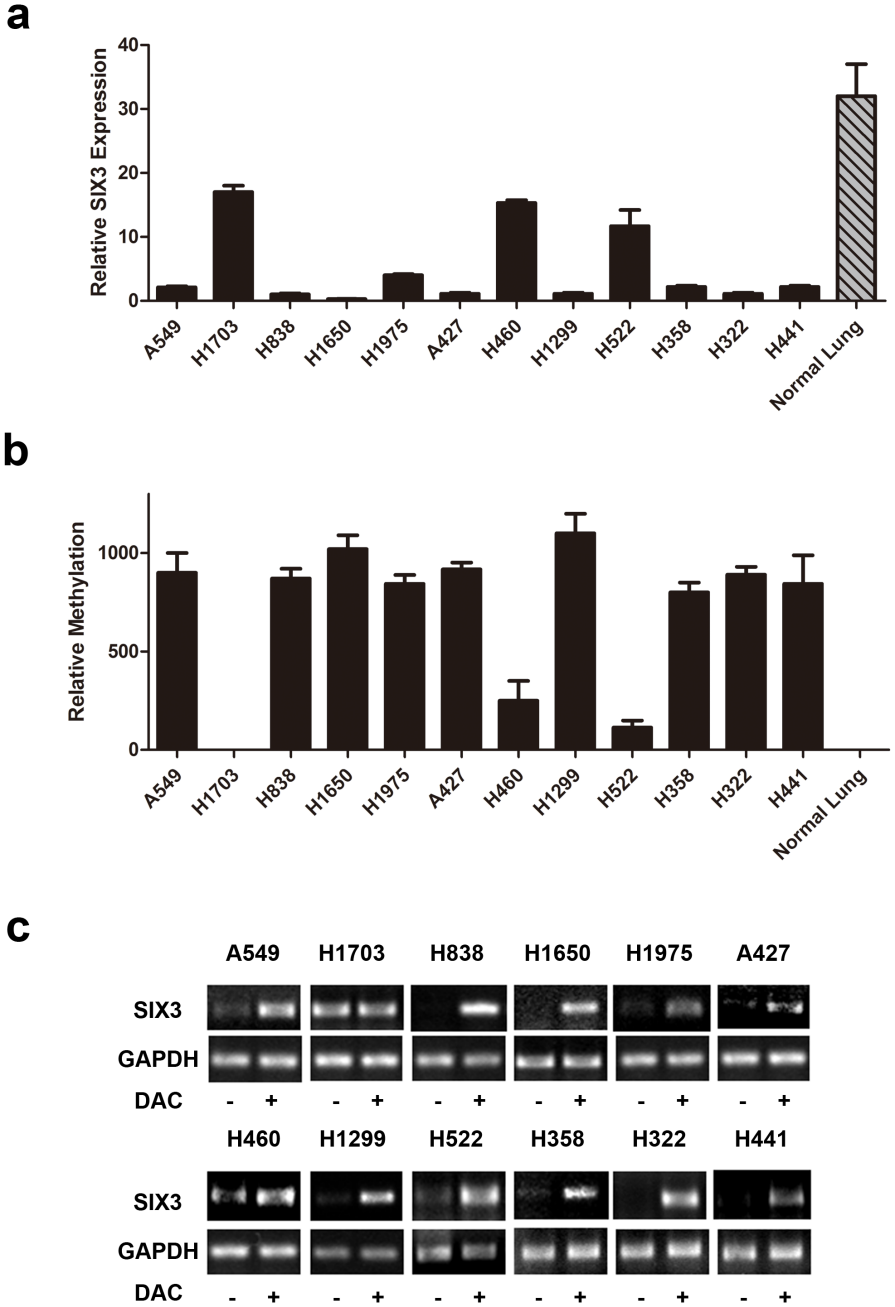
Methylation in the promoter region caused down-regulation of SIX3 expression in NSCLC cell lines. (a) Quantitative RT- PCR of SIX3 expression in NSCLC cell lines and total RNA of normal adult lung tissues was included as a control. (b) Quantitative MSP of the SIX3 promoter region in NSCLC cell lines. Genomic DNA of normal adult lung tissues was included as a control. (c) Semi-quantitative RT-PCR of SIX3 expression after 5 µM 5-aza-2′-deoxycytidine (DAC) treatment in NSCLC cell lines. GAPDH was used as control for RNA quality and loading.

**Figure 2:**
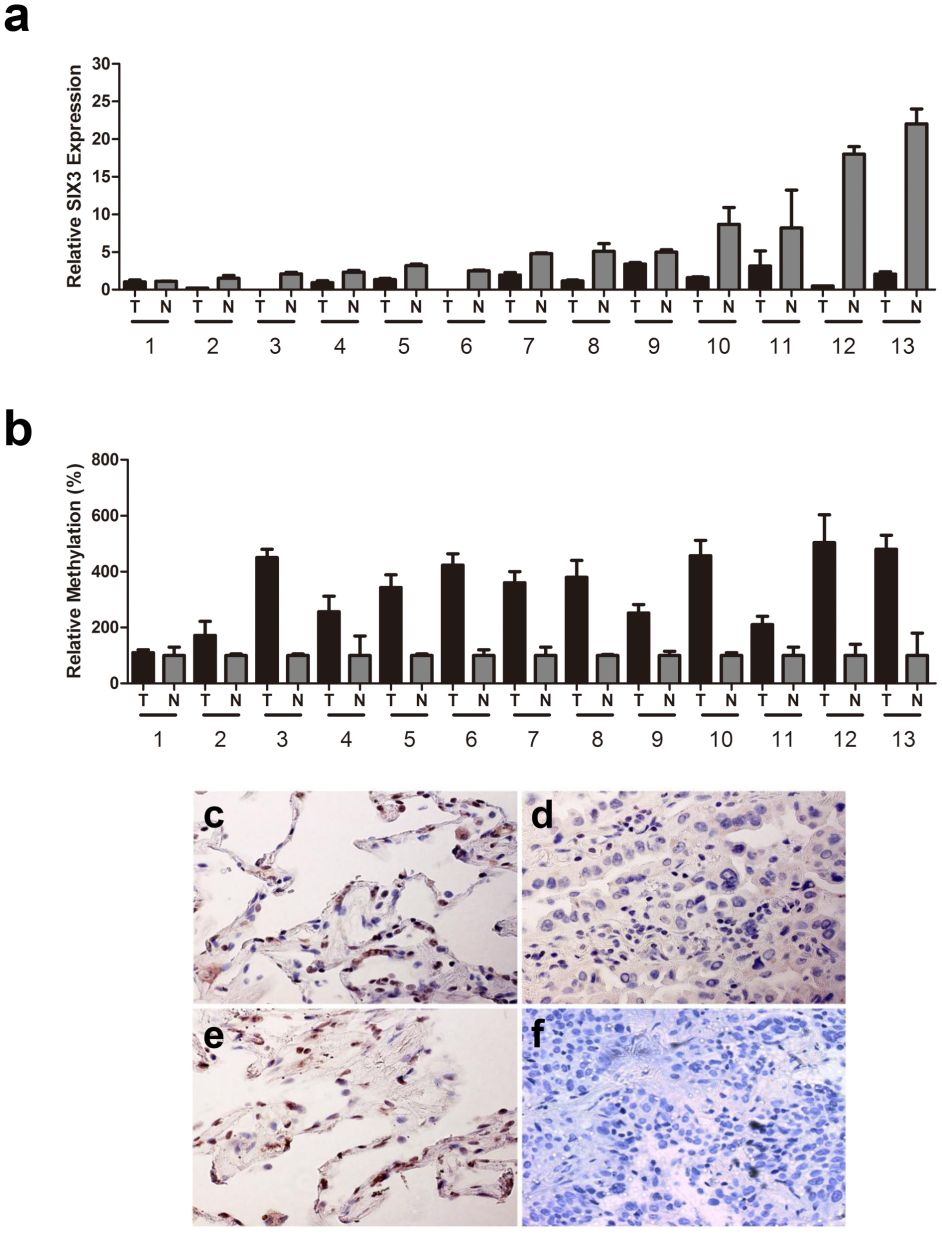
Methylation in the promoter region caused down-regulation of SIX3 expression in NSCLC tissue samples. (a) Quantitative RT-PCR of SIX3 expression in matched pairs of fresh NSCLC tumor tissues and adjacent normal lung tissues, (b) Quantitative MSP of the SIX3 promoter region in matched pairs of fresh NSCLC tumor tissues and adjacent normal lung tissues. (c–f): Immunohistochemistry (IHC) examples of SIX3 protein in matched normal lung tissues (c and e) and tumor tissues (d and f) from two patients (#10 (c and d) and #12 (e and f)). SIX3 protein is represented by positive nuclear staining. Pictures were taken under using a light microscope (magnification 40×).

We first examined SIX3 expression in NSCLC cell lines by quantitative real-time RT-PCR. We observed that 9 of the 12 NSCLC cell lines that we examined lacked SIX3 expression and the other three cell lines (H1703, H460 and H522) had down-regulated SIX3 expression, compared to that in normal adult lung tissues (Fig. 1a). We next examined methylation status of the SIX3 promoter by methylation-specific PCR (MSP) in these NSCLC cell lines. Our results showed that the SIX3 promoter was methylated in these NSCLC cell lines except H1703 (Fig. 1b), correlating with the SIX3 expression levels in these cell lines. As a control, no methylation in the SIX3 promoter in the normal adult lung tissues was observed (Fig. 1b). To confirm the correlation between SIX3 expression and the SIX3 promoter methylation, we treated these NSCLC cell lines with a methyltransferase inhibitor DAC. We found that SIX3 expression levels were dramatically upregulated after DAC treatment in these cell lines except H1703 (Fig. 1c). Next, we examined the SIX3 expression in matched pairs of fresh NSCLC tissue samples. Almost all NSCLC samples (except the case #1) that we examined were found to have significantly down-regulated SIX3 expression than their matched normal samples (Fig. 2a). Consistently, we observed methylation of the SIX3 promoter in these tissue samples with down-regulated SIX3 expression (Fig. 2b). We also examined SIX3 protein expression by IHC in those NSCLC specimens. SIX3 protein down-regulation in tumor tissues when compared with their matched normal tissues was observed (nuclear staining in Fig. 2c and 2e versus 2d and 2f), with an average of 33.6 versus 5.2 positively-stained cells per 40× field respectively (p*<*0.001), consistent with our real-time RT-PCR results. Taken together, our results indicate that down-regulation of SIX3 expression is correlated with the SIX3 promoter methylation in human NSCLC.

### SIX3 Inhibited Proliferation and Migration of NSCLC Cells

**Figure 3:**
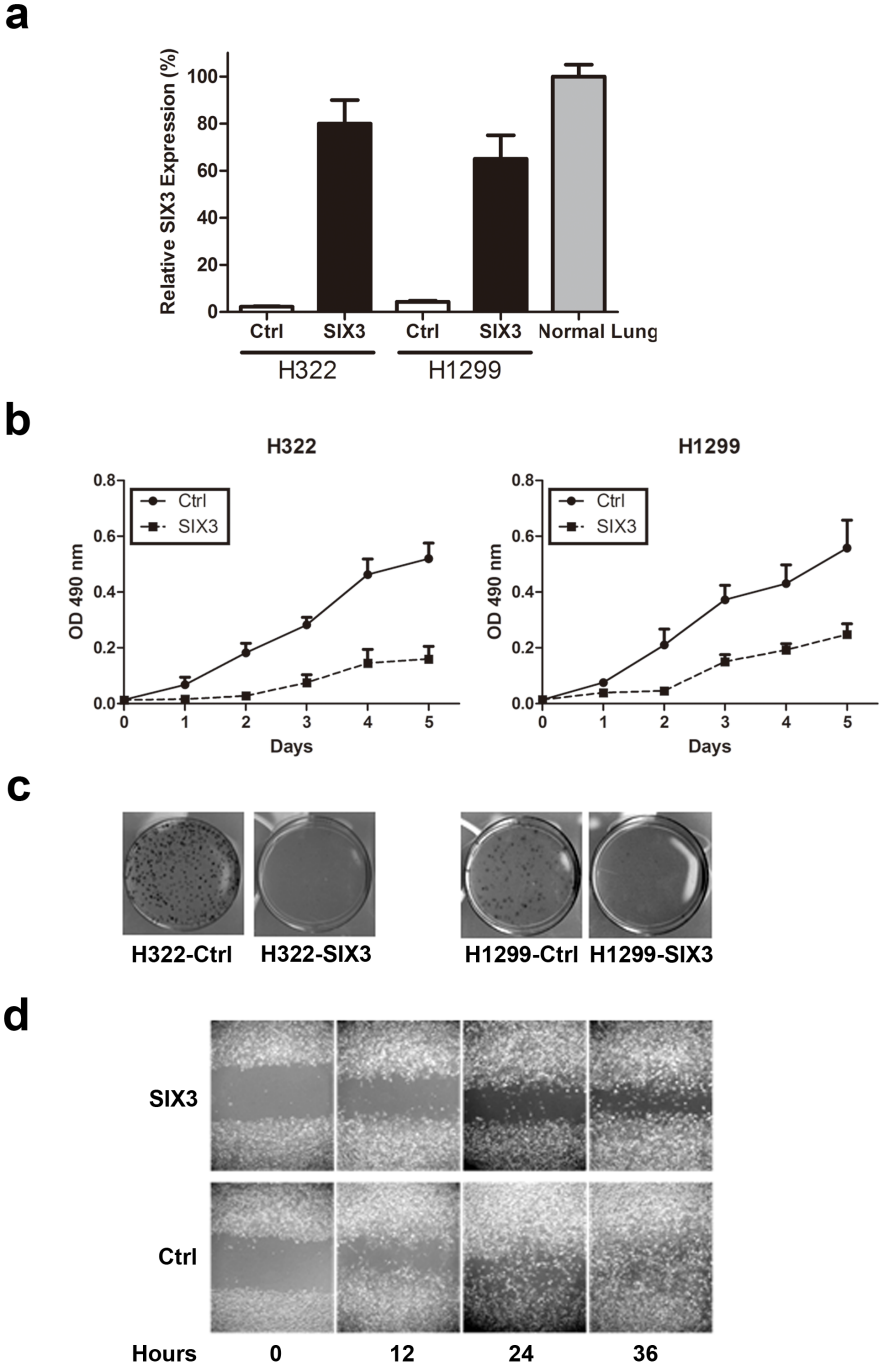
SIX3 suppressed proliferation and migration of NSCLC cells. (a) Quantitative RT-PCR of SIX3 expression in cell lines H1299 and H322 stably transfected with control or SIX3 expression vector. (b) MTS assay of SIX3 stably transfected cells. (c) Colony formation assay of SIX3 stably transfected cells. (d) Wound healing assay of SIX3 stably transfected H1299 cells. Pictures were taken under a light microscope (20×).

**Figure 4:**
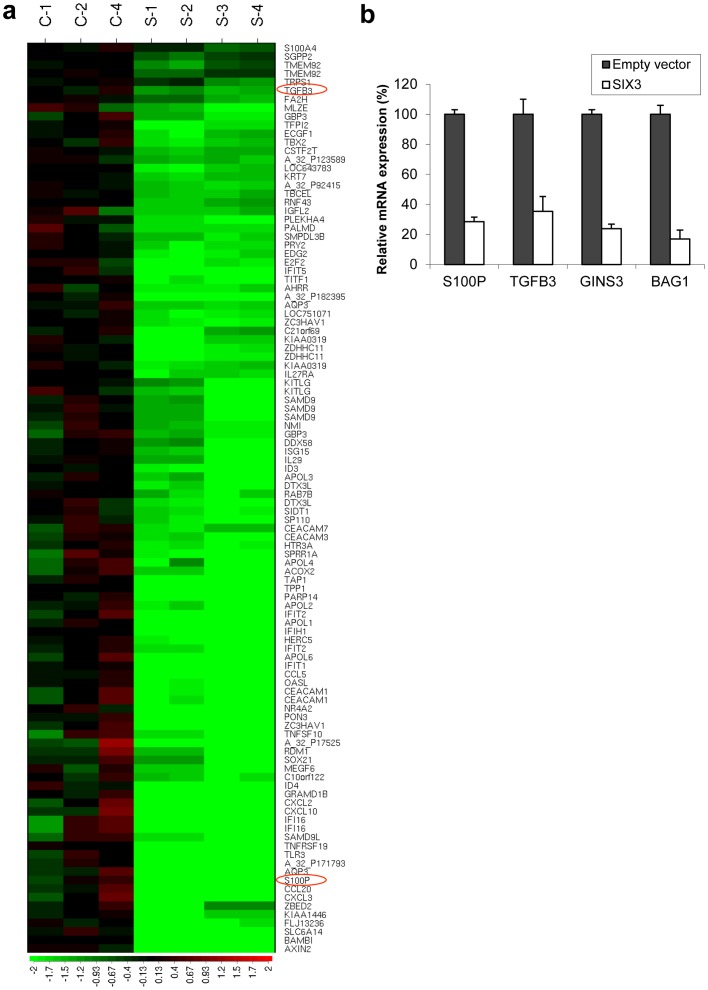
Microarray profiling of NSCLC cell line H1299 stably transfected with SIX3 and empty vector control. (a) Partial heatmap plot of hierarchical clustering of genes that were down-regulated by over-expression of SIX3, including S100P and TGFB3 (highlighted by red cycles) is shown. (b) Validation of down-regulation of some representative genes by over-expression of SIX3 using quantitative RT-PCR.

To investigate roles of SIX3 in NSCLC cells where SIX3 was methylation-silenced, we established stable NSCLC cell lines after transfection with SIX3 cDNA and subsequent G418 selection, and confirmed restoration of SIX3 expression in these stable transfectants (Fig. 3a, SIX3 expression in normal adult lung tissues was used as a reference). We found that the growth of H322 and H1299 cells stably transfected with SIX3 was significantly suppressed compared to the control cells stably transfected with an empty vector (Fig. 3b, p<0.001). This observation was also confirmed by colony formation assay (Fig. 3c, p<0.001). Furthermore, we found that the mobility of H1299 cells stably transfected with SIX3, measured by wound healing assay, was dramatically reduced compared to the control cells (Fig. 3d). These results demonstrate an anti-proliferation and anti-migration function of SIX3 in NSCLC cells, suggesting that SIX3 may play a role as a novel suppressor of malignant NSCLC progression. As a support, our microarray analysis of H1299 cells stably transfected with SIX3 revealed that SIX3 over-expression down-regulated a number of genes related to proliferation and metastasis such as S100P, member of the EF-hand calcium-binding protein family [27]–[30], as well as TGFB3, GINS3, and BAG1[31]–[33] (Fig. 4a, Table S1). Down-regulation of these representative genes by SIX3 over-expression was also confirmed by real-time RT-PCR (Fig. 4b).

**Figure 5:**
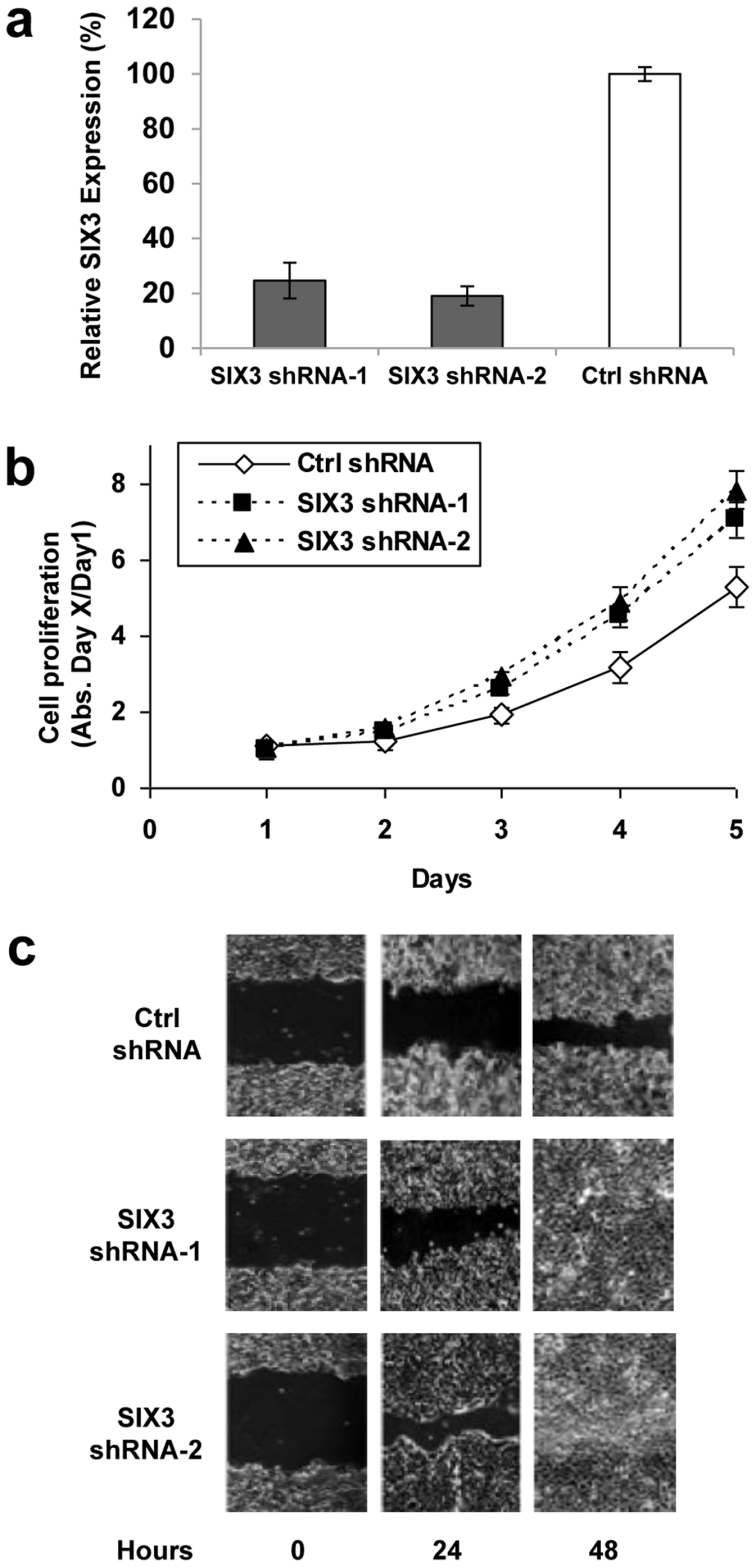
shRNA knock-down of SIX3 in NSCLC cells expressing endogenous SIX3. (a) Quantitative RT-PCR of SIX3 expression in cell line H1703 stably transfected with control shRNA or shRNAs against SIX3 (shRNA-1 or -2). (b) MTS assay of H1703 cells stably transfected with SIX3 shRNAs. (c) Wound healing assay of H1703 cells stably transfected with SIX3 shRNAs. Pictures were taken under a light microscope (20×).

In contrast, when endogenous SIX3 expression in H1703 cells was silenced by anti-SIX3 shRNAs, cell proliferation and migration were promoted (MTS: p = 0.03; migration: p = 0.01) (Fig. 5). These results complement and support the data observed in NSCLC cells H322 and H1299 stably transfected with SIX3 cDNA (Fig. 3).

### SIX3 Expression was Associated with Improved Survival in Lung Adenocarcinoma

**Figure 6:**
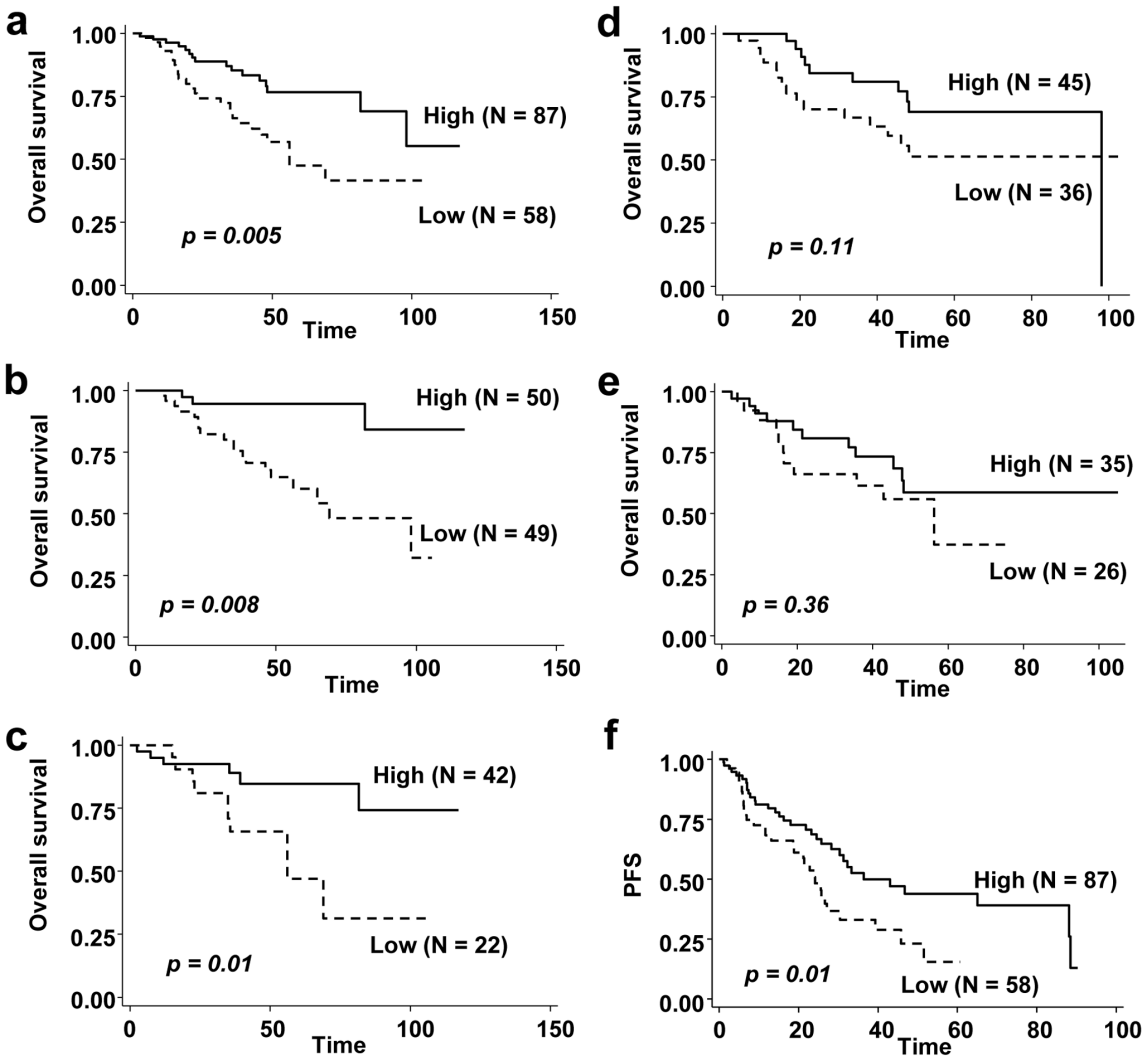
Kaplan-Meier analyses of overall survival and progression-free survival (PFS) based on the SIX3 expression levels. (a) Overall survival in 145 patients with lung adenocarcinomas, (b) Overall survival in 99 Stage I patients, (c) Overall survival in 64 adenocarcinoma patients with BAC features, (d) Overall survival in 81 adenocarcinoma patients without BAC features, (e) Overall survival in 61 Stage II-IV patients, (f) PFS in 145 patients with lung adenocarcinomas.

SIX3 expression was assessed on 160 NSCLC frozen specimens including 145 lung adenocarcinomas by real-time PCR (Table S2). By univariate analysis, we found that SIX3 mRNA expression was significantly associated with gender, tumor size, or ECOG PS (Table 1). There was no significant association between SIX3 mRNA expression and tumor stage, age, smoking, or histology (Table 1). Most interestingly, SIX3 mRNA expression was associated with improved overall survival (p = 0.004) and decreased recurrence (p = 0.02) (Table 1). By Cox multivariate regression analysis, pathologic stages III and IV, and male patients were significantly associated with overall survival (Table 2). Kaplan-Meier analysis using Cox proportional hazards modeling also demonstrated strong associations between SIX3 mRNA expression and survival. Overall survival was significantly better in patients with SIX3-high expression group than in those with SIX3 low expression group in all patients with lung adenocarcinomas (Fig. 6a, p = 0.005), stage I patients (Fig. 6b, p = 0.008), and patients with BAC features (Fig. 6c, p = 0.01). The median overall survival was 69 months for patients with low levels of SIX3 and more than 120 months (not reached at 50%) for those with high levels of SIX3. On the other hand, no significant difference was observed in overall survival of patients without BAC features (Fig. 6d, p = 0.11), and stage II-IV patients (Fig. 6e, p = 0.36). Lastly, there was a significant difference in progression-free survival (PFS) in those patients with lung adenocarcinomas. The median PFS in SIX3-low expression group was 24 months, compared with 36 months for the SIX3-high expression group (Fig. 6f, p = 0.01).

**Table 2:** Cox-multivariate model in lung cancer patients.

Variable		Hazard Ratio	95% CI	p value
SIX3 Expression	Low	–		
	High	0.58	0.31–1.10	0.095
Pathologic Stage	I	–		
	II	1.32	0.50–3.53	0.571
	III	2.44	1.15–5.15	0.02
	IV	2.83	1.05–7.68	0.041
Tumor Size	3 cm or less	–		
	over 3 cm	1.09	0.94–1.27	0.27
Sex	Female	–		
	Male	1.92	1.04–3.55	0.037

## Discussion

Cancer specific promoter methylation of tumor suppressor genes promotes tumor development. It has been reported that homeobox genes were potentially useful as DNA methylation markers for early diagnosis of disease [10]. In present study, our observation of down-regulation of SIX3, one of the homeobox genes, by methylation in NSCLC suggests that SIX3 may function as a transcription repressor in a similar manner in NSCLC as it does during embryonic development. To test this hypothesis, we forced SIX3 expression in NSCLC cell lines lacking endogenous SIX3 expression and observed significant transcriptional suppression of a number of oncogenic genes that are related to proliferation, migration and metastasis. Consistently, over-expressing SIX3 also inhibited cell proliferation and migration measured by MTS, colony formation and wound healing assays, suggesting that SIX3 inhibits the phenotypes of NSCLC cells through transcriptional regulation of those relevant oncogenic pathways. For example, S100P, as well as TGFB3, GINS3 and BAG1 were significantly down-regulated by over-expressing SIX3 in NSCLC cells. S100P protein has been reported to induce metastasis in rodent mammary model systems for breast cancer and to be associated with poor patient outcomes in breast, colon, lung cancer, and esophageal carcinoma[27]–[30]. TGFB3, GINS3 and BAG1 have also been reported to be involved in cancer cell proliferation and invasion[31]–[33]. Interestingly, we noticed that target genes of canonical Wnt pathway such as Axin2 and Myc were down-regulated by over-expressing SIX3 in NSCLC cells. Therefore it is possible that SIX3 regulates the transcription of those proliferation and metastasis related genes directly or indirectly by attenuating the canonical Wnt signaling pathway in NSCLC. Supportively, S100P was reported to be direct downstream targets of the canonical Wnt signaling pathway [34]. However, identification of direct target(s) of SIX3 and further investigations are needed to elucidate mechanisms of how SIX3 regulates those proliferation and metastasis related genes and the relationship between SIX3 and the canonical Wnt signaling pathway in NSCLC.

More importantly, we identified SIX3 as prognostic of increased OS and PFS in lung adenocarcinomas. SIX3 expression was significantly associated with improved OS in stage I patients but not in stage II-III patients. SIX3 expression was also significantly associated with improved OS in adenocarcinoma patients with BAC features. Notably, stage I and BAC patients usually have good prognosis (60–80% 5-year survival rate [35]). BAC is a subset of adenocarcinoma characterized by non-invasive growth along alveolar septae. It is more prevalent in women, non-smokers, and Asians. BAC has a unique clinical and radiological presentation and a different response to systematic treatments compared with conventional lung adenocarcinoma. Previous studies support a multistep process model in lung adenocarcinoma development [36], [37]. Expression difference in genes such as K-ras, p53, Ki-67, and E-cadherin [36] was reported with histologic progression from AAH to BAC to adenocarcinoma. There are also differences in the chromosomal abnormality [36]. Therefore, it is possible that genomic and genetic differences between BAC and adenocarcinomas without BAC features account for the different prognostic values of SIX3. To date, although several prognostic markers for adenocarcinoma and NSCLC exist [38], [39], few are specific to BAC. Our data demonstrate the prognostic significance of SIX3 expression in adenocarcinoma and BAC. The importance of finding reliable prognostic markers for NSCLC, especially early-stage NSCLC, is becoming increasingly apparent. The five-year survival rate for stage I NSCLC is 60–80%, indicating that our current staging methods do not adequately stratify outcome. As a result, the current gold standard of treating early-stage lung cancer by lobectomy and mediastinal lymph node dissection may represent overtreatment in patients with early-stage tumors expressing biomarkers associated with good outcomes. At the same time, because neo-adjuvant and adjuvant therapy is not considered standard of care for early-stage lung cancer, we may be undertreating patients with early-stage tumors expressing biomarkers associated with poor outcomes by surgical resection alone.

In summary, we demonstrate for the first time, the importance of SIX3 as a transcription factor regulating the control of cellular proliferation and migration in the context of lung carcinogenesis, by itself and by suppressing the transcription of oncogenic genes. We also demonstrate that epigenetic events affecting SIX3 lead to its silencing, loss of function and consequent cancer cell proliferation and metastasis. Moreover, we demonstrate clinical relevance of SIX3 expression levels in patients with adenocarcinomas and BAC. Taken together, our results suggest that SIX3 may play an important role as a novel suppressor in NSCLC and it may have potential as a novel prognostic marker for early stage patients of this disease.

## Supporting Information

Table S1 **Differential expression gene list in NSCLC H1299 cells transfected with SIX3 (S) vs. CTRL (C).**
(DOC)

Table S2 **qPCR Analysis of SIX3 Expression in Tissue Samples of NSCLC Patients.**
(DOC)
