# Supplementary material for: Down-Regulation of SIX3 is Associated with Clinical Outcome in Lung Adenocarcinoma
Source: PLoS One. 2013 Aug 16;8(8):e71816. doi: 10.1371/journal.pone.0071816 (PMC3745425; doi:10.1371/journal.pone.0071816)
Supplement: Table S1 — Differential expression gene list in NSCLC H1299 cells transfected with SIX3 (S) vs. CTRL (C). (DOC) [file pone.0071816.s001.doc]

Table S1. Differential expression gene list in NSCLC H1299 cells transfected with SIX3 (S) vs. CTRL (C)

| ID | Symbol | EntrezGene | UniGene | aveCTRL | aveSIX3 | log2FC  (Svs.C) | Adjustp  (Svs.C) | Fdr  (Svs.C) | B  (Svs.C) | Description |
| --- | --- | --- | --- | --- | --- | --- | --- | --- | --- | --- |
| [A_23_P395566](http://arrays.ucsf.edu/cgi-bin/oligo_db.pl?oligo=A_23_P395566) | FBXO31 | [79791](http://www.ncbi.nih.gov/entrez/query.fcgi?db=gene&cmd=Retrieve&dopt=summary&list_uids=79791) | Hs.567582 | 10.3 | 9.3 | -1 | 1 | 0.005 | 2.035 | Homo sapiens F-box protein 31 (FBXO31), mRNA [NM_024735] |
| [A_32_P772230](http://arrays.ucsf.edu/cgi-bin/oligo_db.pl?oligo=A_32_P772230) | SLC25A45 | [283130](http://www.ncbi.nih.gov/entrez/query.fcgi?db=gene&cmd=Retrieve&dopt=summary&list_uids=283130) | Hs.661604 | 7.77 | 6.77 | -1 | 1 | 0.013 | 0.462 | Homo sapiens mRNA for FLJ00351 protein. [AK090434] |
| [A_23_P206661](http://arrays.ucsf.edu/cgi-bin/oligo_db.pl?oligo=A_23_P206661) | NQO1 | [1728](http://www.ncbi.nih.gov/entrez/query.fcgi?db=gene&cmd=Retrieve&dopt=summary&list_uids=1728) | Hs.406515 | 13.55 | 12.54 | -1 | 1 | 0.012 | 0.568 | Homo sapiens NAD(P)H dehydrogenase, quinone 1 (NQO1), transcript variant 1, mRNA [NM_000903] |
| [A_32_P171921](http://arrays.ucsf.edu/cgi-bin/oligo_db.pl?oligo=A_32_P171921) | LOC729597 | [729597](http://www.ncbi.nih.gov/entrez/query.fcgi?db=gene&cmd=Retrieve&dopt=summary&list_uids=729597) | Hs.489449 | 8.87 | 7.86 | -1.01 | 1 | 0.013 | 0.442 | BX339010 Homo sapiens PLACENTA COT 25-NORMALIZED Homo sapiens cDNA clone CS0DI066YH21 5-PRIME, mRNA sequence [BX339010] |
| [A_32_P212920](http://arrays.ucsf.edu/cgi-bin/oligo_db.pl?oligo=A_32_P212920) |  | [NA](../SIX3/NA) |  | 7.56 | 6.55 | -1.01 | 1 | 0.011 | 0.745 | ALU4_HUMAN (P39191) Alu subfamily SB2 sequence contamination warning entry, partial (10%) [THC2754005] |
| [A_23_P55076](http://arrays.ucsf.edu/cgi-bin/oligo_db.pl?oligo=A_23_P55076) | SKIP | [51763](http://www.ncbi.nih.gov/entrez/query.fcgi?db=gene&cmd=Retrieve&dopt=summary&list_uids=51763) | Hs.632238 | 11.57 | 10.56 | -1.01 | 0.013 | 0 | 7.182 | Homo sapiens skeletal muscle and kidney enriched inositol phosphatase (SKIP), transcript variant 2, mRNA [NM_130766] |
| [A_23_P3775](http://arrays.ucsf.edu/cgi-bin/oligo_db.pl?oligo=A_23_P3775) | OGFOD1 | [55239](http://www.ncbi.nih.gov/entrez/query.fcgi?db=gene&cmd=Retrieve&dopt=summary&list_uids=55239) | Hs.231883 | 11.75 | 10.74 | -1.01 | 1 | 0.004 | 2.523 | Homo sapiens 2-oxoglutarate and iron-dependent oxygenase domain containing 1 (OGFOD1), mRNA [NM_018233] |
| [A_23_P352426](http://arrays.ucsf.edu/cgi-bin/oligo_db.pl?oligo=A_23_P352426) | CDRT1 | [374286](http://www.ncbi.nih.gov/entrez/query.fcgi?db=gene&cmd=Retrieve&dopt=summary&list_uids=374286) | Hs.632233 | 7.43 | 6.42 | -1.01 | 1 | 0.012 | 0.501 | Homo sapiens CMT1A duplicated region transcript 1 (CDRT1), mRNA [NM_006382] |
| [A_24_P94351](http://arrays.ucsf.edu/cgi-bin/oligo_db.pl?oligo=A_24_P94351) | C1orf56 | [54964](http://www.ncbi.nih.gov/entrez/query.fcgi?db=gene&cmd=Retrieve&dopt=summary&list_uids=54964) | Hs.549171 | 11.12 | 10.11 | -1.01 | 0.103 | 0.001 | 5.288 | Homo sapiens chromosome 1 open reading frame 56 (C1orf56), mRNA [NM_017860] |
| [A_32_P169735](http://arrays.ucsf.edu/cgi-bin/oligo_db.pl?oligo=A_32_P169735) | TTC8 | [123016](http://www.ncbi.nih.gov/entrez/query.fcgi?db=gene&cmd=Retrieve&dopt=summary&list_uids=123016) | Hs.303055 | 11.16 | 10.15 | -1.01 | 1 | 0.006 | 1.874 | Homo sapiens tetratricopeptide repeat domain 8 (TTC8), transcript variant 1, mRNA [NM_144596] |
| [A_23_P20437](http://arrays.ucsf.edu/cgi-bin/oligo_db.pl?oligo=A_23_P20437) | TIGD5 | [84948](http://www.ncbi.nih.gov/entrez/query.fcgi?db=gene&cmd=Retrieve&dopt=summary&list_uids=84948) | Hs.71574 | 13.29 | 12.28 | -1.01 | 1 | 0.005 | 1.946 | Homo sapiens tigger transposable element derived 5 (TIGD5), mRNA [NM_032862] |
| [A_23_P21376](http://arrays.ucsf.edu/cgi-bin/oligo_db.pl?oligo=A_23_P21376) | MAGI2 | [9863](http://www.ncbi.nih.gov/entrez/query.fcgi?db=gene&cmd=Retrieve&dopt=summary&list_uids=9863) | Hs.654788 | 7.55 | 6.54 | -1.01 | 1 | 0.008 | 1.301 | Homo sapiens membrane associated guanylate kinase, WW and PDZ domain containing 2 (MAGI2), mRNA [NM_012301] |
| [A_23_P129425](http://arrays.ucsf.edu/cgi-bin/oligo_db.pl?oligo=A_23_P129425) | TSNAXIP1 | [55815](http://www.ncbi.nih.gov/entrez/query.fcgi?db=gene&cmd=Retrieve&dopt=summary&list_uids=55815) | Hs.632212 | 7.42 | 6.4 | -1.02 | 1 | 0.004 | 2.609 | Homo sapiens translin-associated factor X interacting protein 1 (TSNAXIP1), mRNA [NM_018430] |
| [A_23_P210210](http://arrays.ucsf.edu/cgi-bin/oligo_db.pl?oligo=A_23_P210210) | EPAS1 | [2034](http://www.ncbi.nih.gov/entrez/query.fcgi?db=gene&cmd=Retrieve&dopt=summary&list_uids=2034) | Hs.468410 | 13.09 | 12.07 | -1.02 | 1 | 0.008 | 1.306 | Homo sapiens endothelial PAS domain protein 1 (EPAS1), mRNA [NM_001430] |
| [A_23_P44112](http://arrays.ucsf.edu/cgi-bin/oligo_db.pl?oligo=A_23_P44112) | LAT | [27040](http://www.ncbi.nih.gov/entrez/query.fcgi?db=gene&cmd=Retrieve&dopt=summary&list_uids=27040) | Hs.632179 | 10.25 | 9.24 | -1.02 | 1 | 0.003 | 3.071 | Homo sapiens linker for activation of T cells (LAT), transcript variant 1, mRNA [NM_014387] |
| [A_23_P120125](http://arrays.ucsf.edu/cgi-bin/oligo_db.pl?oligo=A_23_P120125) | COLEC11 | [78989](http://www.ncbi.nih.gov/entrez/query.fcgi?db=gene&cmd=Retrieve&dopt=summary&list_uids=78989) | Hs.32603 | 8 | 6.98 | -1.02 | 1 | 0.008 | 1.177 | Homo sapiens collectin sub-family member 11 (COLEC11), transcript variant 2, mRNA [NM_199235] |
| [A_23_P217507](http://arrays.ucsf.edu/cgi-bin/oligo_db.pl?oligo=A_23_P217507) | ZBED1 | [9189](http://www.ncbi.nih.gov/entrez/query.fcgi?db=gene&cmd=Retrieve&dopt=summary&list_uids=9189) | Hs.131452 | 10.81 | 9.78 | -1.02 | 1 | 0.004 | 2.335 | Homo sapiens zinc finger, BED-type containing 1 (ZBED1), mRNA [NM_004729] |
| [A_24_P360269](http://arrays.ucsf.edu/cgi-bin/oligo_db.pl?oligo=A_24_P360269) | RNASET2 | [8635](http://www.ncbi.nih.gov/entrez/query.fcgi?db=gene&cmd=Retrieve&dopt=summary&list_uids=8635) | Hs.529989 | 12.02 | 11 | -1.02 | 0.395 | 0.002 | 3.988 | Homo sapiens ribonuclease T2 (RNASET2), mRNA [NM_003730] |
| [A_23_P432385](http://arrays.ucsf.edu/cgi-bin/oligo_db.pl?oligo=A_23_P432385) | H2AFJ | [55766](http://www.ncbi.nih.gov/entrez/query.fcgi?db=gene&cmd=Retrieve&dopt=summary&list_uids=55766) | Hs.524280 | 8.03 | 7.01 | -1.03 | 1 | 0.008 | 1.282 | Homo sapiens H2A histone family, member J (H2AFJ), transcript variant 1, mRNA [NM_018267] |
| [A_23_P76402](http://arrays.ucsf.edu/cgi-bin/oligo_db.pl?oligo=A_23_P76402) | TECT1 | [79600](http://www.ncbi.nih.gov/entrez/query.fcgi?db=gene&cmd=Retrieve&dopt=summary&list_uids=79600) | Hs.211511 | 11.75 | 10.72 | -1.03 | 0.471 | 0.002 | 3.814 | Homo sapiens tectonic 1 (TECT1), transcript variant 3, mRNA [NM_024549] |
| [A_23_P130965](http://arrays.ucsf.edu/cgi-bin/oligo_db.pl?oligo=A_23_P130965) | ARRDC2 | [27106](http://www.ncbi.nih.gov/entrez/query.fcgi?db=gene&cmd=Retrieve&dopt=summary&list_uids=27106) | Hs.515249 | 11.76 | 10.73 | -1.03 | 1 | 0.004 | 2.772 | Homo sapiens arrestin domain containing 2 (ARRDC2), transcript variant 1, mRNA [NM_015683] |
| [A_23_P54079](http://arrays.ucsf.edu/cgi-bin/oligo_db.pl?oligo=A_23_P54079) | OSGEP | [55644](http://www.ncbi.nih.gov/entrez/query.fcgi?db=gene&cmd=Retrieve&dopt=summary&list_uids=55644) | Hs.525196 | 11.77 | 10.74 | -1.03 | 0.069 | 0.001 | 5.669 | Homo sapiens O-sialoglycoprotein endopeptidase (OSGEP), mRNA [NM_017807] |
| [A_32_P512061](http://arrays.ucsf.edu/cgi-bin/oligo_db.pl?oligo=A_32_P512061) | GBAP | [2630](http://www.ncbi.nih.gov/entrez/query.fcgi?db=gene&cmd=Retrieve&dopt=summary&list_uids=2630) | Hs.282997 | 11.78 | 10.74 | -1.03 | 1 | 0.01 | 0.849 | Homo sapiens glucosidase, beta; acid, pseudogene (GBAP) on chromosome 1 [NR_002188] |
| [A_23_P425752](http://arrays.ucsf.edu/cgi-bin/oligo_db.pl?oligo=A_23_P425752) | TRIM14 | [9830](http://www.ncbi.nih.gov/entrez/query.fcgi?db=gene&cmd=Retrieve&dopt=summary&list_uids=9830) | Hs.575631 | 9.04 | 8 | -1.03 | 1 | 0.01 | 0.872 | Homo sapiens tripartite motif-containing 14 (TRIM14), transcript variant 2, mRNA [NM_033219] |
| [A_24_P211106](http://arrays.ucsf.edu/cgi-bin/oligo_db.pl?oligo=A_24_P211106) |  | [NA](../SIX3/NA) | Hs.204044 | 9.26 | 8.22 | -1.04 | 1 | 0.004 | 2.527 | Tumor necrosis factor receptor superfamily member 11A precursor (Receptor activator of NF-KB) (Osteoclast differentiation factor receptor) (ODFR) (CD265 antigen). [Source:Uniprot/SWISSPROT;Acc:Q9Y6Q6] [ENST00000382790] |
| [A_23_P382154](http://arrays.ucsf.edu/cgi-bin/oligo_db.pl?oligo=A_23_P382154) | MRPL43 | [84545](http://www.ncbi.nih.gov/entrez/query.fcgi?db=gene&cmd=Retrieve&dopt=summary&list_uids=84545) | Hs.421848 | 9.85 | 8.81 | -1.04 | 1 | 0.006 | 1.882 | Homo sapiens mitochondrial ribosomal protein L43 (MRPL43), nuclear gene encoding mitochondrial protein, transcript variant 4, mRNA [NM_176794] |
| [A_23_P65983](http://arrays.ucsf.edu/cgi-bin/oligo_db.pl?oligo=A_23_P65983) | CCDC102A | [92922](http://www.ncbi.nih.gov/entrez/query.fcgi?db=gene&cmd=Retrieve&dopt=summary&list_uids=92922) | Hs.644611 | 11.31 | 10.27 | -1.04 | 1 | 0.015 | 0.2 | Homo sapiens coiled-coil domain containing 102A (CCDC102A), mRNA [NM_033212] |
| [A_23_P4679](http://arrays.ucsf.edu/cgi-bin/oligo_db.pl?oligo=A_23_P4679) | ERF | [2077](http://www.ncbi.nih.gov/entrez/query.fcgi?db=gene&cmd=Retrieve&dopt=summary&list_uids=2077) | Hs.655969 | 11.22 | 10.18 | -1.04 | 0.146 | 0.001 | 4.952 | Homo sapiens Ets2 repressor factor (ERF), mRNA [NM_006494] |
| [A_24_P161725](http://arrays.ucsf.edu/cgi-bin/oligo_db.pl?oligo=A_24_P161725) |  | [NA](../SIX3/NA) | Hs.526423 | 10.85 | 9.81 | -1.04 | 1 | 0.015 | 0.195 | Homo sapiens cDNA clone IMAGE:30390722, containing frame-shift errors. [BC089388] |
| [A_24_P568190](http://arrays.ucsf.edu/cgi-bin/oligo_db.pl?oligo=A_24_P568190) | GUSBP1 | [153561](http://www.ncbi.nih.gov/entrez/query.fcgi?db=gene&cmd=Retrieve&dopt=summary&list_uids=153561) | Hs.654588 | 8.29 | 7.25 | -1.04 | 1 | 0.01 | 0.791 | Homo sapiens glucuronidase, beta pseudogene 1, mRNA (cDNA clone IMAGE:4824349), complete cds. [BC035411] |
| [A_23_P208158](http://arrays.ucsf.edu/cgi-bin/oligo_db.pl?oligo=A_23_P208158) | DTNA | [1837](http://www.ncbi.nih.gov/entrez/query.fcgi?db=gene&cmd=Retrieve&dopt=summary&list_uids=1837) | Hs.643454 | 10.23 | 9.18 | -1.05 | 1 | 0.007 | 1.393 | Homo sapiens dystrobrevin, alpha (DTNA), transcript variant 7, mRNA [NM_001392] |
| [A_23_P121396](http://arrays.ucsf.edu/cgi-bin/oligo_db.pl?oligo=A_23_P121396) | DNAJC19 | [131118](http://www.ncbi.nih.gov/entrez/query.fcgi?db=gene&cmd=Retrieve&dopt=summary&list_uids=131118) | Hs.230601 | 10.05 | 9 | -1.05 | 0.785 | 0.003 | 3.312 | Homo sapiens DnaJ (Hsp40) homolog, subfamily C, member 19 (DNAJC19), mRNA [NM_145261] |
| [A_23_P395609](http://arrays.ucsf.edu/cgi-bin/oligo_db.pl?oligo=A_23_P395609) | FAM110B | [90362](http://www.ncbi.nih.gov/entrez/query.fcgi?db=gene&cmd=Retrieve&dopt=summary&list_uids=90362) | Hs.154652 | 8.61 | 7.56 | -1.05 | 0.689 | 0.002 | 3.442 | Homo sapiens family with sequence similarity 110, member B (FAM110B), mRNA [NM_147189] |
| [A_24_P901986](http://arrays.ucsf.edu/cgi-bin/oligo_db.pl?oligo=A_24_P901986) |  | [NA](../SIX3/NA) | Hs.660833 | 7.78 | 6.72 | -1.06 | 1 | 0.015 | 0.167 | Homo sapiens, clone IMAGE:5170250, mRNA. [BC029255] |
| [A_23_P90780](http://arrays.ucsf.edu/cgi-bin/oligo_db.pl?oligo=A_23_P90780) |  | [NA](../SIX3/NA) |  | 8.8 | 7.74 | -1.06 | 1 | 0.003 | 3.007 | Q5I0G3_HUMAN (Q5I0G3) MDH1B protein, partial (43%) [THC2746571] |
| [A_23_P140434](http://arrays.ucsf.edu/cgi-bin/oligo_db.pl?oligo=A_23_P140434) | MYO5C | [55930](http://www.ncbi.nih.gov/entrez/query.fcgi?db=gene&cmd=Retrieve&dopt=summary&list_uids=55930) | Hs.487036 | 11.62 | 10.56 | -1.06 | 1 | 0.013 | 0.375 | Homo sapiens myosin VC (MYO5C), mRNA [NM_018728] |
| [A_23_P13150](http://arrays.ucsf.edu/cgi-bin/oligo_db.pl?oligo=A_23_P13150) | C11orf71 | [54494](http://www.ncbi.nih.gov/entrez/query.fcgi?db=gene&cmd=Retrieve&dopt=summary&list_uids=54494) | Hs.91816 | 9.92 | 8.86 | -1.06 | 1 | 0.015 | 0.216 | Homo sapiens chromosome 11 open reading frame 71 (C11orf71), mRNA [NM_019021] |
| [A_24_P96474](http://arrays.ucsf.edu/cgi-bin/oligo_db.pl?oligo=A_24_P96474) | LDOC1L | [84247](http://www.ncbi.nih.gov/entrez/query.fcgi?db=gene&cmd=Retrieve&dopt=summary&list_uids=84247) | Hs.332795 | 10.64 | 9.58 | -1.06 | 1 | 0.012 | 0.517 | Homo sapiens leucine zipper, down-regulated in cancer 1-like (LDOC1L), mRNA [NM_032287] |
| [A_23_P204296](http://arrays.ucsf.edu/cgi-bin/oligo_db.pl?oligo=A_23_P204296) | RERG | [85004](http://www.ncbi.nih.gov/entrez/query.fcgi?db=gene&cmd=Retrieve&dopt=summary&list_uids=85004) | Hs.199487 | 9.32 | 8.26 | -1.06 | 1 | 0.01 | 0.909 | Homo sapiens RAS-like, estrogen-regulated, growth inhibitor (RERG), mRNA [NM_032918] |
| [A_23_P80974](http://arrays.ucsf.edu/cgi-bin/oligo_db.pl?oligo=A_23_P80974) | TDO2 | [6999](http://www.ncbi.nih.gov/entrez/query.fcgi?db=gene&cmd=Retrieve&dopt=summary&list_uids=6999) | Hs.183671 | 8.81 | 7.75 | -1.06 | 1 | 0.009 | 1.078 | Homo sapiens tryptophan 2,3-dioxygenase (TDO2), mRNA [NM_005651] |
| [A_23_P375281](http://arrays.ucsf.edu/cgi-bin/oligo_db.pl?oligo=A_23_P375281) | TRPV1 | [7442](http://www.ncbi.nih.gov/entrez/query.fcgi?db=gene&cmd=Retrieve&dopt=summary&list_uids=7442) | Hs.655380 | 8.81 | 7.74 | -1.06 | 0.058 | 0.001 | 5.831 | Homo sapiens transient receptor potential cation channel, subfamily V, member 1 (TRPV1), transcript variant 3, mRNA [NM_080706] |
| [A_23_P72850](http://arrays.ucsf.edu/cgi-bin/oligo_db.pl?oligo=A_23_P72850) | MFSD5 | [84975](http://www.ncbi.nih.gov/entrez/query.fcgi?db=gene&cmd=Retrieve&dopt=summary&list_uids=84975) | Hs.654660 | 13.1 | 12.02 | -1.07 | 1 | 0.009 | 1.036 | Homo sapiens major facilitator superfamily domain containing 5 (MFSD5), mRNA [NM_032889] |
| [A_23_P383986](http://arrays.ucsf.edu/cgi-bin/oligo_db.pl?oligo=A_23_P383986) | GALNAC4S-6ST | [51363](http://www.ncbi.nih.gov/entrez/query.fcgi?db=gene&cmd=Retrieve&dopt=summary&list_uids=51363) | Hs.287537 | 13.05 | 11.98 | -1.07 | 1 | 0.011 | 0.728 | Homo sapiens B cell RAG associated protein (GALNAC4S-6ST), mRNA [NM_015892] |
| [A_24_P289471](http://arrays.ucsf.edu/cgi-bin/oligo_db.pl?oligo=A_24_P289471) | RNASET2 | [8635](http://www.ncbi.nih.gov/entrez/query.fcgi?db=gene&cmd=Retrieve&dopt=summary&list_uids=8635) | Hs.529989 | 11.36 | 10.28 | -1.08 | 0.018 | 0 | 6.929 | Homo sapiens ribonuclease T2 (RNASET2), mRNA [NM_003730] |
| [A_23_P434268](http://arrays.ucsf.edu/cgi-bin/oligo_db.pl?oligo=A_23_P434268) |  | [NA](../SIX3/NA) | Hs.520246 | 9.47 | 8.39 | -1.08 | 1 | 0.013 | 0.441 | dopey family member 1 [Source:RefSeq_peptide;Acc:NP_055833] [ENST00000369739] |
| [A_24_P186065](http://arrays.ucsf.edu/cgi-bin/oligo_db.pl?oligo=A_24_P186065) | DHFRL1 | [200895](http://www.ncbi.nih.gov/entrez/query.fcgi?db=gene&cmd=Retrieve&dopt=summary&list_uids=200895) | Hs.448003 | 9.84 | 8.76 | -1.08 | 1 | 0.012 | 0.517 | Homo sapiens dihydrofolate reductase-like 1 (DHFRL1), mRNA [NM_176815] |
| [A_24_P89887](http://arrays.ucsf.edu/cgi-bin/oligo_db.pl?oligo=A_24_P89887) | C9orf3 | [84909](http://www.ncbi.nih.gov/entrez/query.fcgi?db=gene&cmd=Retrieve&dopt=summary&list_uids=84909) | Hs.434253 | 12.83 | 11.75 | -1.08 | 1 | 0.006 | 1.682 | Homo sapiens chromosome 9 open reading frame 3 (C9orf3), mRNA [NM_032823] |
| [A_32_P6653](http://arrays.ucsf.edu/cgi-bin/oligo_db.pl?oligo=A_32_P6653) |  | [NA](../SIX3/NA) | Hs.471148 | 8 | 6.91 | -1.09 | 0.812 | 0.003 | 3.279 | Homo sapiens cDNA clone IMAGE:5276307. [BC041389] |
| [A_32_P120211](http://arrays.ucsf.edu/cgi-bin/oligo_db.pl?oligo=A_32_P120211) |  | [NA](../SIX3/NA) | Hs.43744 | 8.16 | 7.07 | -1.09 | 1 | 0.008 | 1.333 | AI123606 oo15f01.x1 Soares_NSF_F8_9W_OT_PA_P_S1 Homo sapiens cDNA clone IMAGE:1566265 3', mRNA sequence [AI123606] |
| [A_23_P201035](http://arrays.ucsf.edu/cgi-bin/oligo_db.pl?oligo=A_23_P201035) | GBA | [2629](http://www.ncbi.nih.gov/entrez/query.fcgi?db=gene&cmd=Retrieve&dopt=summary&list_uids=2629) | Hs.282997 | 12.95 | 11.86 | -1.09 | 1 | 0.006 | 1.755 | Homo sapiens glucosidase, beta; acid (includes glucosylceramidase) (GBA), transcript variant 4, mRNA [NM_001005749] |
| [A_23_P117411](http://arrays.ucsf.edu/cgi-bin/oligo_db.pl?oligo=A_23_P117411) | FOXG1B | [2290](http://www.ncbi.nih.gov/entrez/query.fcgi?db=gene&cmd=Retrieve&dopt=summary&list_uids=2290) | Hs.649934 | 8.45 | 7.35 | -1.09 | 0.125 | 0.001 | 5.101 | Homo sapiens forkhead box G1B (FOXG1B), mRNA [NM_005249] |
| [A_23_P203558](http://arrays.ucsf.edu/cgi-bin/oligo_db.pl?oligo=A_23_P203558) | HBB | [3043](http://www.ncbi.nih.gov/entrez/query.fcgi?db=gene&cmd=Retrieve&dopt=summary&list_uids=3043) | Hs.654454 | 7.62 | 6.53 | -1.1 | 1 | 0.01 | 0.842 | Homo sapiens hemoglobin, beta (HBB), mRNA [NM_000518] |
| [A_23_P125639](http://arrays.ucsf.edu/cgi-bin/oligo_db.pl?oligo=A_23_P125639) | ZFX | [7543](http://www.ncbi.nih.gov/entrez/query.fcgi?db=gene&cmd=Retrieve&dopt=summary&list_uids=7543) | Hs.336681 | 8.55 | 7.46 | -1.1 | 1 | 0.007 | 1.394 | Homo sapiens zinc finger protein, X-linked (ZFX), mRNA [NM_003410] |
| [A_23_P65967](http://arrays.ucsf.edu/cgi-bin/oligo_db.pl?oligo=A_23_P65967) | FLJ10815 | [55238](http://www.ncbi.nih.gov/entrez/query.fcgi?db=gene&cmd=Retrieve&dopt=summary&list_uids=55238) | Hs.10499 | 10.55 | 9.45 | -1.1 | 1 | 0.003 | 3.017 | Homo sapiens amino acid transporter (FLJ10815), mRNA [NM_018231] |
| [A_23_P38505](http://arrays.ucsf.edu/cgi-bin/oligo_db.pl?oligo=A_23_P38505) | CXCL16 | [58191](http://www.ncbi.nih.gov/entrez/query.fcgi?db=gene&cmd=Retrieve&dopt=summary&list_uids=58191) | Hs.651206 | 10.33 | 9.23 | -1.1 | 0.261 | 0.001 | 4.392 | Homo sapiens chemokine (C-X-C motif) ligand 16 (CXCL16), mRNA [NM_022059] |
| [A_23_P368187](http://arrays.ucsf.edu/cgi-bin/oligo_db.pl?oligo=A_23_P368187) | NAB2 | [4665](http://www.ncbi.nih.gov/entrez/query.fcgi?db=gene&cmd=Retrieve&dopt=summary&list_uids=4665) | Hs.159223 | 9.77 | 8.67 | -1.11 | 1 | 0.008 | 1.222 | Homo sapiens NGFI-A binding protein 2 (EGR1 binding protein 2) (NAB2), mRNA [NM_005967] |
| [A_32_P186157](http://arrays.ucsf.edu/cgi-bin/oligo_db.pl?oligo=A_32_P186157) | LRRC27 | [80313](http://www.ncbi.nih.gov/entrez/query.fcgi?db=gene&cmd=Retrieve&dopt=summary&list_uids=80313) | Hs.119897 | 8.55 | 7.45 | -1.11 | 1 | 0.012 | 0.537 | Homo sapiens leucine rich repeat containing 27 (LRRC27), mRNA [NM_030626] |
| [A_23_P326319](http://arrays.ucsf.edu/cgi-bin/oligo_db.pl?oligo=A_23_P326319) | C16orf45 | [89927](http://www.ncbi.nih.gov/entrez/query.fcgi?db=gene&cmd=Retrieve&dopt=summary&list_uids=89927) | Hs.460095 | 7.21 | 6.1 | -1.12 | 1 | 0.003 | 2.913 | Homo sapiens chromosome 16 open reading frame 45 (C16orf45), mRNA [NM_033201] |
| [A_32_P107644](http://arrays.ucsf.edu/cgi-bin/oligo_db.pl?oligo=A_32_P107644) | FLJ35409 | [400765](http://www.ncbi.nih.gov/entrez/query.fcgi?db=gene&cmd=Retrieve&dopt=summary&list_uids=400765) | Hs.659018 | 8.01 | 6.89 | -1.12 | 0.547 | 0.002 | 3.668 | Homo sapiens cDNA FLJ35409 fis, clone SKNSH2009435. [AK092728] |
| [A_23_P29330](http://arrays.ucsf.edu/cgi-bin/oligo_db.pl?oligo=A_23_P29330) | SMC1B | [27127](http://www.ncbi.nih.gov/entrez/query.fcgi?db=gene&cmd=Retrieve&dopt=summary&list_uids=27127) | Hs.334176 | 7.36 | 6.24 | -1.12 | 0.67 | 0.002 | 3.469 | Homo sapiens structural maintenance of chromosomes 1B (SMC1B), mRNA [NM_148674] |
| [A_23_P144244](http://arrays.ucsf.edu/cgi-bin/oligo_db.pl?oligo=A_23_P144244) |  | [NA](../SIX3/NA) |  | 10.02 | 8.9 | -1.12 | 1 | 0.015 | 0.232 | Q3MHD6_HUMAN (Q3MHD6) COX17 homolog, cytochrome c oxidase assembly protein, partial (98%) [THC2671344] |
| [A_23_P152136](http://arrays.ucsf.edu/cgi-bin/oligo_db.pl?oligo=A_23_P152136) | GINS3 | [64785](http://www.ncbi.nih.gov/entrez/query.fcgi?db=gene&cmd=Retrieve&dopt=summary&list_uids=64785) | Hs.47125 | 13.61 | 12.49 | -1.12 | 0.795 | 0.003 | 3.3 | Homo sapiens GINS complex subunit 3 (Psf3 homolog) (GINS3), mRNA [NM_022770] |
| [A_23_P46141](http://arrays.ucsf.edu/cgi-bin/oligo_db.pl?oligo=A_23_P46141) | CTSS | [1520](http://www.ncbi.nih.gov/entrez/query.fcgi?db=gene&cmd=Retrieve&dopt=summary&list_uids=1520) | Hs.181301 | 7.94 | 6.82 | -1.12 | 1 | 0.009 | 1.019 | Homo sapiens cathepsin S (CTSS), mRNA [NM_004079] |
| [A_24_P43391](http://arrays.ucsf.edu/cgi-bin/oligo_db.pl?oligo=A_24_P43391) | TMEM165 | [55858](http://www.ncbi.nih.gov/entrez/query.fcgi?db=gene&cmd=Retrieve&dopt=summary&list_uids=55858) | Hs.479766 | 10.56 | 9.43 | -1.13 | 0.449 | 0.002 | 3.861 | Homo sapiens transmembrane protein 165 (TMEM165), mRNA [NM_018475] |
| [A_32_P210168](http://arrays.ucsf.edu/cgi-bin/oligo_db.pl?oligo=A_32_P210168) | LOC388135 | [388135](http://www.ncbi.nih.gov/entrez/query.fcgi?db=gene&cmd=Retrieve&dopt=summary&list_uids=388135) | Hs.40794 | 8.46 | 7.32 | -1.14 | 0.56 | 0.002 | 3.646 | Homo sapiens similar to RIKEN cDNA 6030419C18 gene (LOC388135), mRNA [NM_001039614] |
| [A_24_P298894](http://arrays.ucsf.edu/cgi-bin/oligo_db.pl?oligo=A_24_P298894) | LOC399956 | [399956](http://www.ncbi.nih.gov/entrez/query.fcgi?db=gene&cmd=Retrieve&dopt=summary&list_uids=399956) | Hs.595921 | 13.89 | 12.75 | -1.14 | 0.22 | 0.001 | 4.559 | PREDICTED: Homo sapiens similar to Keratin, type II cytoskeletal 8 (Cytokeratin-8) (CK-8) (Keratin-8) (K8) (LOC399956), mRNA [XR_018724] |
| [A_23_P23947](http://arrays.ucsf.edu/cgi-bin/oligo_db.pl?oligo=A_23_P23947) | MAP3K8 | [1326](http://www.ncbi.nih.gov/entrez/query.fcgi?db=gene&cmd=Retrieve&dopt=summary&list_uids=1326) | Hs.432453 | 9.08 | 7.94 | -1.14 | 1 | 0.016 | 0.102 | Homo sapiens mitogen-activated protein kinase kinase kinase 8 (MAP3K8), mRNA [NM_005204] |
| [A_23_P382705](http://arrays.ucsf.edu/cgi-bin/oligo_db.pl?oligo=A_23_P382705) | TMTC2 | [160335](http://www.ncbi.nih.gov/entrez/query.fcgi?db=gene&cmd=Retrieve&dopt=summary&list_uids=160335) | Hs.577775 | 10.02 | 8.88 | -1.14 | 0.621 | 0.002 | 3.544 | Homo sapiens transmembrane and tetratricopeptide repeat containing 2 (TMTC2), mRNA [NM_152588] |
| [A_23_P62465](http://arrays.ucsf.edu/cgi-bin/oligo_db.pl?oligo=A_23_P62465) | ZFY | [7544](http://www.ncbi.nih.gov/entrez/query.fcgi?db=gene&cmd=Retrieve&dopt=summary&list_uids=7544) | Hs.522845 | 9.54 | 8.4 | -1.14 | 1 | 0.003 | 2.929 | Homo sapiens zinc finger protein, Y-linked (ZFY), mRNA [NM_003411] |
| [A_23_P81770](http://arrays.ucsf.edu/cgi-bin/oligo_db.pl?oligo=A_23_P81770) | PTP4A1 | [7803](http://www.ncbi.nih.gov/entrez/query.fcgi?db=gene&cmd=Retrieve&dopt=summary&list_uids=7803) | Hs.227777 | 14.06 | 12.92 | -1.14 | 0.278 | 0.002 | 4.331 | Homo sapiens protein tyrosine phosphatase type IVA, member 1 (PTP4A1), mRNA [NM_003463] |
| [A_23_P31399](http://arrays.ucsf.edu/cgi-bin/oligo_db.pl?oligo=A_23_P31399) | PON2 | [5445](http://www.ncbi.nih.gov/entrez/query.fcgi?db=gene&cmd=Retrieve&dopt=summary&list_uids=5445) | Hs.530077 | 14.08 | 12.94 | -1.14 | 0.519 | 0.002 | 3.721 | Homo sapiens paraoxonase 2 (PON2), transcript variant 1, mRNA [NM_000305] |
| [A_32_P218482](http://arrays.ucsf.edu/cgi-bin/oligo_db.pl?oligo=A_32_P218482) |  | [NA](../SIX3/NA) | Hs.32769 | 9.36 | 8.22 | -1.15 | 1 | 0.009 | 1.146 | Homo sapiens mRNA full length insert cDNA clone EUROIMAGE 362430. [AL109706] |
| [A_24_P53976](http://arrays.ucsf.edu/cgi-bin/oligo_db.pl?oligo=A_24_P53976) | GLUL | [2752](http://www.ncbi.nih.gov/entrez/query.fcgi?db=gene&cmd=Retrieve&dopt=summary&list_uids=2752) | Hs.518525 | 10.43 | 9.28 | -1.15 | 1 | 0.009 | 1.018 | Homo sapiens glutamate-ammonia ligase (glutamine synthetase) (GLUL), transcript variant 1, mRNA [NM_002065] |
| [A_24_P76725](http://arrays.ucsf.edu/cgi-bin/oligo_db.pl?oligo=A_24_P76725) | WDR42A | [50717](http://www.ncbi.nih.gov/entrez/query.fcgi?db=gene&cmd=Retrieve&dopt=summary&list_uids=50717) | Hs.632447 | 7.99 | 6.83 | -1.15 | 1 | 0.004 | 2.714 | Homo sapiens WD repeat domain 42A, mRNA (cDNA clone IMAGE:5743945), complete cds. [BC111063] |
| [A_23_P166109](http://arrays.ucsf.edu/cgi-bin/oligo_db.pl?oligo=A_23_P166109) | FLRT3 | [23767](http://www.ncbi.nih.gov/entrez/query.fcgi?db=gene&cmd=Retrieve&dopt=summary&list_uids=23767) | Hs.41296 | 9.34 | 8.18 | -1.16 | 1 | 0.008 | 1.348 | Homo sapiens fibronectin leucine rich transmembrane protein 3 (FLRT3), transcript variant 2, mRNA [NM_198391] |
| [A_23_P124438](http://arrays.ucsf.edu/cgi-bin/oligo_db.pl?oligo=A_23_P124438) | ZNF718 | [255403](http://www.ncbi.nih.gov/entrez/query.fcgi?db=gene&cmd=Retrieve&dopt=summary&list_uids=255403) | Hs.428579 | 7.9 | 6.74 | -1.16 | 1 | 0.017 | 0.001 | Homo sapiens zinc finger protein 718 (ZNF718), mRNA [NM_001039127] |
| [A_23_P335695](http://arrays.ucsf.edu/cgi-bin/oligo_db.pl?oligo=A_23_P335695) | OBSL1 | [23363](http://www.ncbi.nih.gov/entrez/query.fcgi?db=gene&cmd=Retrieve&dopt=summary&list_uids=23363) | Hs.526594 | 8.84 | 7.67 | -1.17 | 1 | 0.009 | 1.014 | Homo sapiens cDNA FLJ13792 fis, clone THYRO1000072, weakly similar to MYOSIN LIGHT CHAIN KINASE, SMOOTH MUSCLE AND NON-MUSCLE ISOZYMES (EC 2.7.1.117). [AK023854] |
| [A_23_P64617](http://arrays.ucsf.edu/cgi-bin/oligo_db.pl?oligo=A_23_P64617) | FZD4 | [8322](http://www.ncbi.nih.gov/entrez/query.fcgi?db=gene&cmd=Retrieve&dopt=summary&list_uids=8322) | Hs.591968 | 10.13 | 8.96 | -1.17 | 1 | 0.016 | 0.043 | Homo sapiens frizzled homolog 4 (Drosophila) (FZD4), mRNA [NM_012193] |
| [A_32_P45588](http://arrays.ucsf.edu/cgi-bin/oligo_db.pl?oligo=A_32_P45588) |  | [NA](../SIX3/NA) | Hs.416216 | 8.68 | 7.51 | -1.17 | 1 | 0.005 | 2.007 | BM474343 AGENCOURT_6489981 NIH_MGC_71 Homo sapiens cDNA clone IMAGE:5521645 5', mRNA sequence [BM474343] |
| [A_32_P152586](http://arrays.ucsf.edu/cgi-bin/oligo_db.pl?oligo=A_32_P152586) |  | [NA](../SIX3/NA) | Hs.94367 | 8.48 | 7.31 | -1.17 | 1 | 0.016 | 0.032 | Q7KZF6_HUMAN (Q7KZF6) Thyroid transcription factor 1, partial (15%) [THC2636875] |
| [A_32_P407245](http://arrays.ucsf.edu/cgi-bin/oligo_db.pl?oligo=A_32_P407245) | FLJ13236 | [79962](http://www.ncbi.nih.gov/entrez/query.fcgi?db=gene&cmd=Retrieve&dopt=summary&list_uids=79962) | Hs.659300 | 8.91 | 7.73 | -1.17 | 1 | 0.004 | 2.709 | Homo sapiens hypothetical protein FLJ13236 (FLJ13236), mRNA [NM_024902] |
| [A_24_P156781](http://arrays.ucsf.edu/cgi-bin/oligo_db.pl?oligo=A_24_P156781) | PIK3R3 | [8503](http://www.ncbi.nih.gov/entrez/query.fcgi?db=gene&cmd=Retrieve&dopt=summary&list_uids=8503) | Hs.655387 | 8.35 | 7.17 | -1.18 | 1 | 0.006 | 1.854 | Homo sapiens phosphoinositide-3-kinase, regulatory subunit 3 (p55, gamma) (PIK3R3), mRNA [NM_003629] |
| [A_23_P214267](http://arrays.ucsf.edu/cgi-bin/oligo_db.pl?oligo=A_23_P214267) | GPR110 | [266977](http://www.ncbi.nih.gov/entrez/query.fcgi?db=gene&cmd=Retrieve&dopt=summary&list_uids=266977) | Hs.256897 | 14.02 | 12.85 | -1.18 | 1 | 0.015 | 0.133 | Homo sapiens G protein-coupled receptor 110 (GPR110), transcript variant 1, mRNA [NM_153840] |
| [A_23_P134176](http://arrays.ucsf.edu/cgi-bin/oligo_db.pl?oligo=A_23_P134176) | SOD2 | [6648](http://www.ncbi.nih.gov/entrez/query.fcgi?db=gene&cmd=Retrieve&dopt=summary&list_uids=6648) | Hs.487046 | 15.29 | 14.11 | -1.18 | 1 | 0.005 | 1.99 | Homo sapiens superoxide dismutase 2, mitochondrial (SOD2), nuclear gene encoding mitochondrial protein, transcript variant 1, mRNA [NM_000636] |
| [A_23_P254434](http://arrays.ucsf.edu/cgi-bin/oligo_db.pl?oligo=A_23_P254434) | RFPL2 | [10739](http://www.ncbi.nih.gov/entrez/query.fcgi?db=gene&cmd=Retrieve&dopt=summary&list_uids=10739) | Hs.157427 | 7.73 | 6.55 | -1.18 | 0.348 | 0.002 | 4.112 | Homo sapiens ret finger protein-like 2 (RFPL2), mRNA [NM_006605] |
| [A_23_P434352](http://arrays.ucsf.edu/cgi-bin/oligo_db.pl?oligo=A_23_P434352) | CAST | [831](http://www.ncbi.nih.gov/entrez/query.fcgi?db=gene&cmd=Retrieve&dopt=summary&list_uids=831) | Hs.440961 | 15.45 | 14.27 | -1.18 | 0.17 | 0.001 | 4.805 | Homo sapiens calpastatin (CAST), transcript variant 1, mRNA [NM_001750] |
| [A_23_P124456](http://arrays.ucsf.edu/cgi-bin/oligo_db.pl?oligo=A_23_P124456) | PDE4D | [5144](http://www.ncbi.nih.gov/entrez/query.fcgi?db=gene&cmd=Retrieve&dopt=summary&list_uids=5144) | Hs.654358 | 8.12 | 6.94 | -1.18 | 0.422 | 0.002 | 3.923 | Homo sapiens phosphodiesterase 4D, cAMP-specific (phosphodiesterase E3 dunce homolog, Drosophila) (PDE4D), mRNA [NM_006203] |
| [A_23_P154526](http://arrays.ucsf.edu/cgi-bin/oligo_db.pl?oligo=A_23_P154526) | GRB14 | [2888](http://www.ncbi.nih.gov/entrez/query.fcgi?db=gene&cmd=Retrieve&dopt=summary&list_uids=2888) | Hs.411881 | 12.67 | 11.48 | -1.19 | 0.079 | 0.001 | 5.536 | Homo sapiens growth factor receptor-bound protein 14 (GRB14), mRNA [NM_004490] |
| [A_23_P90099](http://arrays.ucsf.edu/cgi-bin/oligo_db.pl?oligo=A_23_P90099) | UNQ501 | [374882](http://www.ncbi.nih.gov/entrez/query.fcgi?db=gene&cmd=Retrieve&dopt=summary&list_uids=374882) | Hs.8036 | 16.06 | 14.87 | -1.19 | 1 | 0.005 | 2.096 | Homo sapiens MBC3205 (UNQ501), mRNA [NM_198536] |
| [A_23_P203601](http://arrays.ucsf.edu/cgi-bin/oligo_db.pl?oligo=A_23_P203601) | UCP3 | [7352](http://www.ncbi.nih.gov/entrez/query.fcgi?db=gene&cmd=Retrieve&dopt=summary&list_uids=7352) | Hs.101337 | 8.77 | 7.58 | -1.2 | 1 | 0.009 | 0.984 | Homo sapiens uncoupling protein 3 (mitochondrial, proton carrier) (UCP3), nuclear gene encoding mitochondrial protein, transcript variant long, mRNA [NM_003356] |
| [A_23_P132388](http://arrays.ucsf.edu/cgi-bin/oligo_db.pl?oligo=A_23_P132388) | SCO2 | [9997](http://www.ncbi.nih.gov/entrez/query.fcgi?db=gene&cmd=Retrieve&dopt=summary&list_uids=9997) | Hs.658057 | 12.82 | 11.62 | -1.2 | 1 | 0.003 | 3.03 | Homo sapiens SCO cytochrome oxidase deficient homolog 2 (yeast) (SCO2), nuclear gene encoding mitochondrial protein, mRNA [NM_005138] |
| [A_23_P112220](http://arrays.ucsf.edu/cgi-bin/oligo_db.pl?oligo=A_23_P112220) | INSL4 | [3641](http://www.ncbi.nih.gov/entrez/query.fcgi?db=gene&cmd=Retrieve&dopt=summary&list_uids=3641) | Hs.418506 | 7.23 | 6.03 | -1.2 | 1 | 0.004 | 2.339 | Homo sapiens insulin-like 4 (placenta) (INSL4), mRNA [NM_002195] |
| [A_23_P124300](http://arrays.ucsf.edu/cgi-bin/oligo_db.pl?oligo=A_23_P124300) | BCMO1 | [53630](http://www.ncbi.nih.gov/entrez/query.fcgi?db=gene&cmd=Retrieve&dopt=summary&list_uids=53630) | Hs.212172 | 8.15 | 6.95 | -1.2 | 1 | 0.009 | 1.033 | Homo sapiens beta-carotene 15,15'-monooxygenase 1 (BCMO1), mRNA [NM_017429] |
| [A_32_P226205](http://arrays.ucsf.edu/cgi-bin/oligo_db.pl?oligo=A_32_P226205) | ZFHX2 | [85446](http://www.ncbi.nih.gov/entrez/query.fcgi?db=gene&cmd=Retrieve&dopt=summary&list_uids=85446) | Hs.525247 | 9.8 | 8.59 | -1.21 | 1 | 0.015 | 0.171 | Homo sapiens mRNA for KIAA1762 protein, partial cds. [AB051549] |
| [A_32_P149640](http://arrays.ucsf.edu/cgi-bin/oligo_db.pl?oligo=A_32_P149640) | EPHA5 | [2044](http://www.ncbi.nih.gov/entrez/query.fcgi?db=gene&cmd=Retrieve&dopt=summary&list_uids=2044) | Hs.654492 | 7.26 | 6.05 | -1.21 | 1 | 0.01 | 0.887 | Homo sapiens mRNA; cDNA DKFZp686C0686 (from clone DKFZp686C0686); complete cds. [BX537946] |
| [A_23_P345065](http://arrays.ucsf.edu/cgi-bin/oligo_db.pl?oligo=A_23_P345065) | SCLY | [51540](http://www.ncbi.nih.gov/entrez/query.fcgi?db=gene&cmd=Retrieve&dopt=summary&list_uids=51540) | Hs.512606 | 10.61 | 9.4 | -1.21 | 1 | 0.008 | 1.318 | Homo sapiens selenocysteine lyase (SCLY), mRNA [NM_016510] |
| [A_23_P37505](http://arrays.ucsf.edu/cgi-bin/oligo_db.pl?oligo=A_23_P37505) | DYX1C1 | [161582](http://www.ncbi.nih.gov/entrez/query.fcgi?db=gene&cmd=Retrieve&dopt=summary&list_uids=161582) | Hs.126403 | 8.74 | 7.53 | -1.22 | 0.801 | 0.003 | 3.292 | Homo sapiens dyslexia susceptibility 1 candidate 1 (DYX1C1), transcript variant 1, mRNA [NM_130810] |
| [A_23_P345928](http://arrays.ucsf.edu/cgi-bin/oligo_db.pl?oligo=A_23_P345928) | C12orf26 | [84190](http://www.ncbi.nih.gov/entrez/query.fcgi?db=gene&cmd=Retrieve&dopt=summary&list_uids=84190) | Hs.506222 | 9.25 | 8.03 | -1.22 | 1 | 0.005 | 2.216 | Homo sapiens chromosome 12 open reading frame 26 (C12orf26), mRNA [NM_032230] |
| [A_23_P76151](http://arrays.ucsf.edu/cgi-bin/oligo_db.pl?oligo=A_23_P76151) | NAB2 | [4665](http://www.ncbi.nih.gov/entrez/query.fcgi?db=gene&cmd=Retrieve&dopt=summary&list_uids=4665) | Hs.159223 | 12 | 10.77 | -1.22 | 0.097 | 0.001 | 5.338 | Homo sapiens NGFI-A binding protein 2 (EGR1 binding protein 2) (NAB2), mRNA [NM_005967] |
| [A_32_P146659](http://arrays.ucsf.edu/cgi-bin/oligo_db.pl?oligo=A_32_P146659) | LOC401431 | [401431](http://www.ncbi.nih.gov/entrez/query.fcgi?db=gene&cmd=Retrieve&dopt=summary&list_uids=401431) | Hs.652227 | 8.52 | 7.29 | -1.23 | 1 | 0.007 | 1.46 | Homo sapiens hypothetical gene LOC401431 (LOC401431), mRNA [NM_001008745] |
| [A_32_P85753](http://arrays.ucsf.edu/cgi-bin/oligo_db.pl?oligo=A_32_P85753) |  | [NA](../SIX3/NA) |  | 7.28 | 6.05 | -1.23 | 0.01 | 0 | 7.439 |  |
| [A_23_P133174](http://arrays.ucsf.edu/cgi-bin/oligo_db.pl?oligo=A_23_P133174) | TMEM165 | [55858](http://www.ncbi.nih.gov/entrez/query.fcgi?db=gene&cmd=Retrieve&dopt=summary&list_uids=55858) | Hs.479766 | 12.26 | 11.03 | -1.23 | 0.406 | 0.002 | 3.96 | Homo sapiens transmembrane protein 165 (TMEM165), mRNA [NM_018475] |
| [A_23_P357504](http://arrays.ucsf.edu/cgi-bin/oligo_db.pl?oligo=A_23_P357504) |  | [NA](../SIX3/NA) |  | 9.03 | 7.79 | -1.23 | 1 | 0.011 | 0.71 |  |
| [A_23_P41765](http://arrays.ucsf.edu/cgi-bin/oligo_db.pl?oligo=A_23_P41765) | IRF1 | [3659](http://www.ncbi.nih.gov/entrez/query.fcgi?db=gene&cmd=Retrieve&dopt=summary&list_uids=3659) | Hs.436061 | 10.83 | 9.59 | -1.24 | 0.038 | 0.001 | 6.211 | Homo sapiens interferon regulatory factor 1 (IRF1), mRNA [NM_002198] |
| [A_23_P136724](http://arrays.ucsf.edu/cgi-bin/oligo_db.pl?oligo=A_23_P136724) | LOC344887 | [344887](http://www.ncbi.nih.gov/entrez/query.fcgi?db=gene&cmd=Retrieve&dopt=summary&list_uids=344887) | Hs.128803 | 13.92 | 12.68 | -1.24 | 1 | 0.005 | 2.081 | Homo sapiens mRNA; cDNA DKFZp686B14224 (from clone DKFZp686B14224). [BX640843] |
| [A_23_P250353](http://arrays.ucsf.edu/cgi-bin/oligo_db.pl?oligo=A_23_P250353) | HERC6 | [55008](http://www.ncbi.nih.gov/entrez/query.fcgi?db=gene&cmd=Retrieve&dopt=summary&list_uids=55008) | Hs.529317 | 11.62 | 10.37 | -1.24 | 1 | 0.004 | 2.358 | Homo sapiens hect domain and RLD 6 (HERC6), mRNA [NM_017912] |
| [A_32_P205110](http://arrays.ucsf.edu/cgi-bin/oligo_db.pl?oligo=A_32_P205110) | FOXC1 | [2296](http://www.ncbi.nih.gov/entrez/query.fcgi?db=gene&cmd=Retrieve&dopt=summary&list_uids=2296) | Hs.348883 | 12.01 | 10.76 | -1.25 | 1 | 0.004 | 2.694 | Homo sapiens forkhead box C1 (FOXC1), mRNA [NM_001453] |
| [A_24_P598919](http://arrays.ucsf.edu/cgi-bin/oligo_db.pl?oligo=A_24_P598919) |  | [NA](../SIX3/NA) |  | 9.76 | 8.51 | -1.25 | 1 | 0.003 | 3.037 | Q6TDT1_HUMAN (Q6TDT1) Protein transactivated by hepatitis B virus E antigen, partial (34%) [THC2713545] |
| [A_23_P88404](http://arrays.ucsf.edu/cgi-bin/oligo_db.pl?oligo=A_23_P88404) | TGFB3 | [7043](http://www.ncbi.nih.gov/entrez/query.fcgi?db=gene&cmd=Retrieve&dopt=summary&list_uids=7043) | Hs.592317 | 10.29 | 9.04 | -1.25 | 1 | 0.006 | 1.795 | Homo sapiens transforming growth factor, beta 3 (TGFB3), mRNA [NM_003239] |
| [A_23_P218807](http://arrays.ucsf.edu/cgi-bin/oligo_db.pl?oligo=A_23_P218807) | ZC3H7B | [23264](http://www.ncbi.nih.gov/entrez/query.fcgi?db=gene&cmd=Retrieve&dopt=summary&list_uids=23264) | Hs.592188 | 10.38 | 9.13 | -1.26 | 1 | 0.011 | 0.742 | Homo sapiens zinc finger CCCH-type containing 7B (ZC3H7B), mRNA [NM_017590] |
| [A_24_P68079](http://arrays.ucsf.edu/cgi-bin/oligo_db.pl?oligo=A_24_P68079) | LBA1 | [9881](http://www.ncbi.nih.gov/entrez/query.fcgi?db=gene&cmd=Retrieve&dopt=summary&list_uids=9881) | Hs.170999 | 7.17 | 5.9 | -1.26 | 1 | 0.005 | 2.257 | CDNA FLJ39359 fis, clone PEBLM2004290. (Fragment). [Source:Uniprot/SPTREMBL;Acc:Q8N8K0] [ENST00000301807] |
| [A_23_P73012](http://arrays.ucsf.edu/cgi-bin/oligo_db.pl?oligo=A_23_P73012) | C9orf3 | [84909](http://www.ncbi.nih.gov/entrez/query.fcgi?db=gene&cmd=Retrieve&dopt=summary&list_uids=84909) | Hs.434253 | 12.51 | 11.25 | -1.27 | 0.006 | 0 | 7.88 | Homo sapiens chromosome 9 open reading frame 3 (C9orf3), mRNA [NM_032823] |
| [A_23_P75786](http://arrays.ucsf.edu/cgi-bin/oligo_db.pl?oligo=A_23_P75786) | SLC15A3 | [51296](http://www.ncbi.nih.gov/entrez/query.fcgi?db=gene&cmd=Retrieve&dopt=summary&list_uids=51296) | Hs.237856 | 8.13 | 6.86 | -1.27 | 1 | 0.006 | 1.884 | Homo sapiens solute carrier family 15, member 3 (SLC15A3), mRNA [NM_016582] |
| [A_23_P159255](http://arrays.ucsf.edu/cgi-bin/oligo_db.pl?oligo=A_23_P159255) | PTPRM | [5797](http://www.ncbi.nih.gov/entrez/query.fcgi?db=gene&cmd=Retrieve&dopt=summary&list_uids=5797) | Hs.49774 | 10.81 | 9.54 | -1.27 | 1 | 0.009 | 1.131 | Homo sapiens protein tyrosine phosphatase, receptor type, M (PTPRM), mRNA [NM_002845] |
| [A_24_P524462](http://arrays.ucsf.edu/cgi-bin/oligo_db.pl?oligo=A_24_P524462) |  | [NA](../SIX3/NA) |  | 9.31 | 8.04 | -1.27 | 0.614 | 0.002 | 3.554 |  |
| [A_23_P337753](http://arrays.ucsf.edu/cgi-bin/oligo_db.pl?oligo=A_23_P337753) | LOC93349 | [93349](http://www.ncbi.nih.gov/entrez/query.fcgi?db=gene&cmd=Retrieve&dopt=summary&list_uids=93349) | Hs.662198 | 8.18 | 6.9 | -1.27 | 1 | 0.012 | 0.517 | Homo sapiens hypothetical protein BC004921 (LOC93349), mRNA [NM_138402] |
| [A_32_P187176](http://arrays.ucsf.edu/cgi-bin/oligo_db.pl?oligo=A_32_P187176) | MRRF | [92399](http://www.ncbi.nih.gov/entrez/query.fcgi?db=gene&cmd=Retrieve&dopt=summary&list_uids=92399) | Hs.368011 | 11.37 | 10.09 | -1.28 | 1 | 0.004 | 2.61 | Homo sapiens mitochondrial ribosome recycling factor (MRRF), nuclear gene encoding mitochondrial protein, transcript variant 1, mRNA [NM_138777] |
| [A_23_P164814](http://arrays.ucsf.edu/cgi-bin/oligo_db.pl?oligo=A_23_P164814) | C19orf57 | [79173](http://www.ncbi.nih.gov/entrez/query.fcgi?db=gene&cmd=Retrieve&dopt=summary&list_uids=79173) | Hs.143288 | 10.27 | 8.99 | -1.28 | 1 | 0.004 | 2.365 | Homo sapiens chromosome 19 open reading frame 57 (C19orf57), mRNA [NM_024323] |
| [A_23_P71328](http://arrays.ucsf.edu/cgi-bin/oligo_db.pl?oligo=A_23_P71328) | MATN2 | [4147](http://www.ncbi.nih.gov/entrez/query.fcgi?db=gene&cmd=Retrieve&dopt=summary&list_uids=4147) | Hs.189445 | 8.6 | 7.29 | -1.3 | 1 | 0.009 | 0.987 | Homo sapiens matrilin 2 (MATN2), transcript variant 2, mRNA [NM_030583] |
| [A_24_P149124](http://arrays.ucsf.edu/cgi-bin/oligo_db.pl?oligo=A_24_P149124) | C5orf13 | [9315](http://www.ncbi.nih.gov/entrez/query.fcgi?db=gene&cmd=Retrieve&dopt=summary&list_uids=9315) | Hs.36053 | 10.5 | 9.19 | -1.31 | 0.779 | 0.003 | 3.32 | Homo sapiens chromosome 5 open reading frame 13 (C5orf13), mRNA [NM_004772] |
| [A_23_P102950](http://arrays.ucsf.edu/cgi-bin/oligo_db.pl?oligo=A_23_P102950) | TSGA2 | [89765](http://www.ncbi.nih.gov/entrez/query.fcgi?db=gene&cmd=Retrieve&dopt=summary&list_uids=89765) | Hs.661069 | 8.52 | 7.21 | -1.31 | 0.155 | 0.001 | 4.895 | Homo sapiens testis specific A2 homolog (mouse) (TSGA2), mRNA [NM_080860] |
| [A_23_P95594](http://arrays.ucsf.edu/cgi-bin/oligo_db.pl?oligo=A_23_P95594) | NAT1 | [9](http://www.ncbi.nih.gov/entrez/query.fcgi?db=gene&cmd=Retrieve&dopt=summary&list_uids=9) | Hs.591847 | 8.75 | 7.44 | -1.31 | 1 | 0.013 | 0.454 | Homo sapiens N-acetyltransferase 1 (arylamine N-acetyltransferase) (NAT1), mRNA [NM_000662] |
| [A_23_P29723](http://arrays.ucsf.edu/cgi-bin/oligo_db.pl?oligo=A_23_P29723) | SGOL1 | [151648](http://www.ncbi.nih.gov/entrez/query.fcgi?db=gene&cmd=Retrieve&dopt=summary&list_uids=151648) | Hs.105153 | 8.75 | 7.43 | -1.31 | 1 | 0.015 | 0.213 | Homo sapiens shugoshin-like 1 (S. pombe) (SGOL1), transcript variant A2, mRNA [NM_001012410] |
| [A_23_P146654](http://arrays.ucsf.edu/cgi-bin/oligo_db.pl?oligo=A_23_P146654) | BAG1 | [573](http://www.ncbi.nih.gov/entrez/query.fcgi?db=gene&cmd=Retrieve&dopt=summary&list_uids=573) | Hs.377484 | 14.8 | 13.48 | -1.31 | 1 | 0.004 | 2.485 | Homo sapiens BCL2-associated athanogene (BAG1), mRNA [NM_004323] |
| [A_24_P933838](http://arrays.ucsf.edu/cgi-bin/oligo_db.pl?oligo=A_24_P933838) |  | [NA](../SIX3/NA) | Hs.490551 | 9.45 | 8.13 | -1.32 | 1 | 0.006 | 1.702 | Ubiquitin-associated protein 2-like (Protein NICE-4). [Source:Uniprot/SWISSPROT;Acc:Q14157] [ENST00000317720] |
| [A_32_P542928](http://arrays.ucsf.edu/cgi-bin/oligo_db.pl?oligo=A_32_P542928) | ZNF789 | [285989](http://www.ncbi.nih.gov/entrez/query.fcgi?db=gene&cmd=Retrieve&dopt=summary&list_uids=285989) | Hs.440384 | 8.27 | 6.94 | -1.33 | 1 | 0.004 | 2.718 | Homo sapiens cDNA FLJ16554 fis, clone SPLEN2016135. [AK131429] |
| [A_23_P420417](http://arrays.ucsf.edu/cgi-bin/oligo_db.pl?oligo=A_23_P420417) | TLCD1 | [116238](http://www.ncbi.nih.gov/entrez/query.fcgi?db=gene&cmd=Retrieve&dopt=summary&list_uids=116238) | Hs.499952 | 13.53 | 12.2 | -1.33 | 0.434 | 0.002 | 3.896 | Homo sapiens TLC domain containing 1 (TLCD1), mRNA [NM_138463] |
| [A_23_P47691](http://arrays.ucsf.edu/cgi-bin/oligo_db.pl?oligo=A_23_P47691) | TRIM21 | [6737](http://www.ncbi.nih.gov/entrez/query.fcgi?db=gene&cmd=Retrieve&dopt=summary&list_uids=6737) | Hs.532357 | 9.3 | 7.95 | -1.34 | 1 | 0.004 | 2.579 | Homo sapiens tripartite motif-containing 21 (TRIM21), mRNA [NM_003141] |
| [A_24_P68908](http://arrays.ucsf.edu/cgi-bin/oligo_db.pl?oligo=A_24_P68908) | LOC344887 | [344887](http://www.ncbi.nih.gov/entrez/query.fcgi?db=gene&cmd=Retrieve&dopt=summary&list_uids=344887) | Hs.128803 | 13.04 | 11.7 | -1.34 | 1 | 0.004 | 2.343 | Homo sapiens mRNA; cDNA DKFZp686B14224 (from clone DKFZp686B14224). [BX640843] |
| [A_32_P56759](http://arrays.ucsf.edu/cgi-bin/oligo_db.pl?oligo=A_32_P56759) | PARP14 | [54625](http://www.ncbi.nih.gov/entrez/query.fcgi?db=gene&cmd=Retrieve&dopt=summary&list_uids=54625) | Hs.518203 | 8.24 | 6.9 | -1.34 | 1 | 0.007 | 1.467 | Homo sapiens poly (ADP-ribose) polymerase family, member 14 (PARP14), mRNA [NM_017554] |
| [A_24_P337012](http://arrays.ucsf.edu/cgi-bin/oligo_db.pl?oligo=A_24_P337012) | LOC93349 | [93349](http://www.ncbi.nih.gov/entrez/query.fcgi?db=gene&cmd=Retrieve&dopt=summary&list_uids=93349) | Hs.662198 | 7.97 | 6.62 | -1.35 | 1 | 0.005 | 2.038 | Homo sapiens hypothetical protein BC004921 (LOC93349), mRNA [NM_138402] |
| [A_32_P119744](http://arrays.ucsf.edu/cgi-bin/oligo_db.pl?oligo=A_32_P119744) | LOC344595 | [344595](http://www.ncbi.nih.gov/entrez/query.fcgi?db=gene&cmd=Retrieve&dopt=summary&list_uids=344595) | Hs.677855 | 9.37 | 8.02 | -1.35 | 1 | 0.011 | 0.657 | Homo sapiens hypothetical LOC344595, mRNA (cDNA clone IMAGE:5760770). [BC039550] |
| [A_24_P256513](http://arrays.ucsf.edu/cgi-bin/oligo_db.pl?oligo=A_24_P256513) | AGGF1 | [55109](http://www.ncbi.nih.gov/entrez/query.fcgi?db=gene&cmd=Retrieve&dopt=summary&list_uids=55109) | Hs.634849 | 9.76 | 8.4 | -1.36 | 1 | 0.004 | 2.449 | Homo sapiens angiogenic factor with G patch and FHA domains 1, mRNA (cDNA clone IMAGE:3659316), complete cds. [BC002828] |
| [A_23_P37127](http://arrays.ucsf.edu/cgi-bin/oligo_db.pl?oligo=A_23_P37127) | FOXA1 | [3169](http://www.ncbi.nih.gov/entrez/query.fcgi?db=gene&cmd=Retrieve&dopt=summary&list_uids=3169) | Hs.163484 | 11.44 | 10.08 | -1.36 | 0.12 | 0.001 | 5.14 | Homo sapiens forkhead box A1 (FOXA1), mRNA [NM_004496] |
| [A_23_P215956](http://arrays.ucsf.edu/cgi-bin/oligo_db.pl?oligo=A_23_P215956) | MYC | [4609](http://www.ncbi.nih.gov/entrez/query.fcgi?db=gene&cmd=Retrieve&dopt=summary&list_uids=4609) | Hs.202453 | 13.87 | 12.51 | -1.36 | 1 | 0.008 | 1.205 | Homo sapiens v-myc myelocytomatosis viral oncogene homolog (avian) (MYC), mRNA [NM_002467] |
| [A_23_P111804](http://arrays.ucsf.edu/cgi-bin/oligo_db.pl?oligo=A_23_P111804) | PARP12 | [64761](http://www.ncbi.nih.gov/entrez/query.fcgi?db=gene&cmd=Retrieve&dopt=summary&list_uids=64761) | Hs.12646 | 10.47 | 9.1 | -1.36 | 1 | 0.013 | 0.367 | Homo sapiens poly (ADP-ribose) polymerase family, member 12 (PARP12), mRNA [NM_022750] |
| [A_23_P62188](http://arrays.ucsf.edu/cgi-bin/oligo_db.pl?oligo=A_23_P62188) | KIAA1166 | [55906](http://www.ncbi.nih.gov/entrez/query.fcgi?db=gene&cmd=Retrieve&dopt=summary&list_uids=55906) | Hs.28249 | 11.3 | 9.93 | -1.37 | 1 | 0.006 | 1.739 | Homo sapiens KIAA1166 (KIAA1166), mRNA [NM_018684] |
| [A_23_P32404](http://arrays.ucsf.edu/cgi-bin/oligo_db.pl?oligo=A_23_P32404) | ISG20 | [3669](http://www.ncbi.nih.gov/entrez/query.fcgi?db=gene&cmd=Retrieve&dopt=summary&list_uids=3669) | Hs.459265 | 16.89 | 15.51 | -1.38 | 1 | 0.004 | 2.442 | Homo sapiens interferon stimulated exonuclease gene 20kDa (ISG20), mRNA [NM_002201] |
| [A_23_P106675](http://arrays.ucsf.edu/cgi-bin/oligo_db.pl?oligo=A_23_P106675) | PLCG2 | [5336](http://www.ncbi.nih.gov/entrez/query.fcgi?db=gene&cmd=Retrieve&dopt=summary&list_uids=5336) | Hs.413111 | 12.02 | 10.65 | -1.38 | 1 | 0.006 | 1.736 | Homo sapiens phospholipase C, gamma 2 (phosphatidylinositol-specific) (PLCG2), mRNA [NM_002661] |
| [A_32_P123589](http://arrays.ucsf.edu/cgi-bin/oligo_db.pl?oligo=A_32_P123589) |  | [NA](../SIX3/NA) | Hs.76884 | 9.15 | 7.77 | -1.38 | 0.379 | 0.002 | 4.027 | AW327568 dq04b07.y1 NIH_MGC_2 Homo sapiens cDNA clone IMAGE:2846557 3', mRNA sequence [AW327568] |
| [A_24_P795230](http://arrays.ucsf.edu/cgi-bin/oligo_db.pl?oligo=A_24_P795230) |  | [NA](../SIX3/NA) | Hs.660929 | 9.96 | 8.58 | -1.39 | 0.284 | 0.002 | 4.309 | Homo sapiens cDNA FLJ14635 fis, clone NT2RP2001196. [AK027541] |
| [A_23_P40453](http://arrays.ucsf.edu/cgi-bin/oligo_db.pl?oligo=A_23_P40453) | CBR3 | [874](http://www.ncbi.nih.gov/entrez/query.fcgi?db=gene&cmd=Retrieve&dopt=summary&list_uids=874) | Hs.154510 | 11.88 | 10.49 | -1.39 | 1 | 0.004 | 2.455 | Homo sapiens carbonyl reductase 3 (CBR3), mRNA [NM_001236] |
| [A_24_P347431](http://arrays.ucsf.edu/cgi-bin/oligo_db.pl?oligo=A_24_P347431) | FOXA1 | [3169](http://www.ncbi.nih.gov/entrez/query.fcgi?db=gene&cmd=Retrieve&dopt=summary&list_uids=3169) | Hs.163484 | 13.02 | 11.62 | -1.39 | 0.09 | 0.001 | 5.411 | Homo sapiens forkhead box A1 (FOXA1), mRNA [NM_004496] |
| [A_24_P64781](http://arrays.ucsf.edu/cgi-bin/oligo_db.pl?oligo=A_24_P64781) | KITLG | [4254](http://www.ncbi.nih.gov/entrez/query.fcgi?db=gene&cmd=Retrieve&dopt=summary&list_uids=4254) | Hs.1048 | 8.34 | 6.95 | -1.4 | 1 | 0.009 | 1.103 | Homo sapiens KIT ligand (KITLG), transcript variant b, mRNA [NM_000899] |
| [A_23_P12730](http://arrays.ucsf.edu/cgi-bin/oligo_db.pl?oligo=A_23_P12730) | CSTF2T | [23283](http://www.ncbi.nih.gov/entrez/query.fcgi?db=gene&cmd=Retrieve&dopt=summary&list_uids=23283) | Hs.591358 | 10.68 | 9.29 | -1.4 | 0.236 | 0.001 | 4.491 | Homo sapiens cleavage stimulation factor, 3' pre-RNA, subunit 2, 64kDa, tau variant (CSTF2T), mRNA [NM_015235] |
| [A_23_P3934](http://arrays.ucsf.edu/cgi-bin/oligo_db.pl?oligo=A_23_P3934) | RNF43 | [54894](http://www.ncbi.nih.gov/entrez/query.fcgi?db=gene&cmd=Retrieve&dopt=summary&list_uids=54894) | Hs.656319 | 12.48 | 11.07 | -1.41 | 0.008 | 0 | 7.643 | Homo sapiens ring finger protein 43 (RNF43), mRNA [NM_017763] |
| [A_23_P404902](http://arrays.ucsf.edu/cgi-bin/oligo_db.pl?oligo=A_23_P404902) |  | [NA](../SIX3/NA) | Hs.519574 | 9.3 | 7.89 | -1.41 | 0.379 | 0.002 | 4.027 | apical protein 2 [Source:RefSeq_peptide;Acc:NP_597713] [ENST00000378679] |
| [A_24_P917650](http://arrays.ucsf.edu/cgi-bin/oligo_db.pl?oligo=A_24_P917650) |  | [NA](../SIX3/NA) | Hs.657863 | 8.76 | 7.35 | -1.41 | 1 | 0.008 | 1.196 | Homo sapiens cDNA FLJ13014 fis, clone NT2RP3000592. [AK023076] |
| [A_32_P122494](http://arrays.ucsf.edu/cgi-bin/oligo_db.pl?oligo=A_32_P122494) |  | [NA](../SIX3/NA) | Hs.533336 | 7.95 | 6.54 | -1.41 | 0.002 | 0 | 8.751 | AI652920 wb40g09.x1 NCI_CGAP_GC6 Homo sapiens cDNA clone IMAGE:2308192 3' similar to SW:NMA_HUMAN Q13145 PUTATIVE TRANSMEMBRANE PROTEIN NMA PRECURSOR. ;, mRNA sequence [AI652920] |
| [A_23_P130343](http://arrays.ucsf.edu/cgi-bin/oligo_db.pl?oligo=A_23_P130343) | KCTD1 | [284252](http://www.ncbi.nih.gov/entrez/query.fcgi?db=gene&cmd=Retrieve&dopt=summary&list_uids=284252) | Hs.526630 | 12.64 | 11.22 | -1.42 | 1 | 0.006 | 1.877 | Homo sapiens potassium channel tetramerisation domain containing 1 (KCTD1), mRNA [NM_198991] |
| [A_23_P24586](http://arrays.ucsf.edu/cgi-bin/oligo_db.pl?oligo=A_23_P24586) | PHACS | [84680](http://www.ncbi.nih.gov/entrez/query.fcgi?db=gene&cmd=Retrieve&dopt=summary&list_uids=84680) | Hs.126706 | 7.72 | 6.3 | -1.42 | 1 | 0.008 | 1.259 | Homo sapiens 1-aminocyclopropane-1-carboxylate synthase (PHACS), mRNA [NM_032592] |
| [A_23_P63668](http://arrays.ucsf.edu/cgi-bin/oligo_db.pl?oligo=A_23_P63668) | IFIT5 | [24138](http://www.ncbi.nih.gov/entrez/query.fcgi?db=gene&cmd=Retrieve&dopt=summary&list_uids=24138) | Hs.252839 | 11.77 | 10.34 | -1.42 | 0.074 | 0.001 | 5.592 | Homo sapiens interferon-induced protein with tetratricopeptide repeats 5 (IFIT5), mRNA [NM_012420] |
| [A_32_P153361](http://arrays.ucsf.edu/cgi-bin/oligo_db.pl?oligo=A_32_P153361) |  | [NA](../SIX3/NA) |  | 8.41 | 6.99 | -1.42 | 1 | 0.007 | 1.456 |  |
| [A_24_P247316](http://arrays.ucsf.edu/cgi-bin/oligo_db.pl?oligo=A_24_P247316) | LOC116349 | [116349](http://www.ncbi.nih.gov/entrez/query.fcgi?db=gene&cmd=Retrieve&dopt=summary&list_uids=116349) | Hs.446702 | 10.19 | 8.77 | -1.42 | 1 | 0.015 | 0.235 | Homo sapiens hypothetical protein BC014011, mRNA (cDNA clone MGC:35369 IMAGE:5183143), complete cds. [BC029796] |
| [A_23_P160720](http://arrays.ucsf.edu/cgi-bin/oligo_db.pl?oligo=A_23_P160720) | SNFT | [55509](http://www.ncbi.nih.gov/entrez/query.fcgi?db=gene&cmd=Retrieve&dopt=summary&list_uids=55509) | Hs.62919 | 12.23 | 10.79 | -1.44 | 1 | 0.007 | 1.389 | Homo sapiens Jun dimerization protein p21SNFT (SNFT), mRNA [NM_018664] |
| [A_32_P162797](http://arrays.ucsf.edu/cgi-bin/oligo_db.pl?oligo=A_32_P162797) |  | [NA](../SIX3/NA) |  | 10.87 | 9.43 | -1.45 | 1 | 0.007 | 1.476 |  |
| [A_23_P320261](http://arrays.ucsf.edu/cgi-bin/oligo_db.pl?oligo=A_23_P320261) | DMKN | [93099](http://www.ncbi.nih.gov/entrez/query.fcgi?db=gene&cmd=Retrieve&dopt=summary&list_uids=93099) | Hs.417795 | 14.62 | 13.17 | -1.45 | 1 | 0.004 | 2.389 | Homo sapiens dermokine (DMKN), transcript variant 2, mRNA [NM_033317] |
| [A_24_P921264](http://arrays.ucsf.edu/cgi-bin/oligo_db.pl?oligo=A_24_P921264) |  | [NA](../SIX3/NA) |  | 9.94 | 8.49 | -1.45 | 1 | 0.005 | 2.272 |  |
| [A_23_P27606](http://arrays.ucsf.edu/cgi-bin/oligo_db.pl?oligo=A_23_P27606) | IL27RA | [9466](http://www.ncbi.nih.gov/entrez/query.fcgi?db=gene&cmd=Retrieve&dopt=summary&list_uids=9466) | Hs.132781 | 13.06 | 11.6 | -1.46 | 1 | 0.004 | 2.453 | Homo sapiens interleukin 27 receptor, alpha (IL27RA), mRNA [NM_004843] |
| [A_23_P301247](http://arrays.ucsf.edu/cgi-bin/oligo_db.pl?oligo=A_23_P301247) | HIST2H2AC | [8338](http://www.ncbi.nih.gov/entrez/query.fcgi?db=gene&cmd=Retrieve&dopt=summary&list_uids=8338) | Hs.408067 | 11.34 | 9.88 | -1.46 | 1 | 0.016 | 0.095 | Homo sapiens histone cluster 2, H2ac (HIST2H2AC), mRNA [NM_003517] |
| [A_23_P410017](http://arrays.ucsf.edu/cgi-bin/oligo_db.pl?oligo=A_23_P410017) | TBCEL | [219899](http://www.ncbi.nih.gov/entrez/query.fcgi?db=gene&cmd=Retrieve&dopt=summary&list_uids=219899) | Hs.632108 | 8.1 | 6.63 | -1.47 | 0.021 | 0 | 6.761 | Homo sapiens leucine rich repeat containing 35, mRNA (cDNA clone IMAGE:3913004). [BC020501] |
| [A_23_P321160](http://arrays.ucsf.edu/cgi-bin/oligo_db.pl?oligo=A_23_P321160) | ZNF594 | [84622](http://www.ncbi.nih.gov/entrez/query.fcgi?db=gene&cmd=Retrieve&dopt=summary&list_uids=84622) | Hs.658402 | 8.92 | 7.44 | -1.48 | 1 | 0.003 | 2.855 | Homo sapiens mRNA for KIAA1871 protein, partial cds. [AB058774] |
| [A_24_P197964](http://arrays.ucsf.edu/cgi-bin/oligo_db.pl?oligo=A_24_P197964) | TRIM14 | [9830](http://www.ncbi.nih.gov/entrez/query.fcgi?db=gene&cmd=Retrieve&dopt=summary&list_uids=9830) | Hs.575631 | 10.01 | 8.53 | -1.48 | 1 | 0.005 | 2.141 | Homo sapiens tripartite motif-containing 14 (TRIM14), transcript variant 1, mRNA [NM_014788] |
| [A_23_P219072](http://arrays.ucsf.edu/cgi-bin/oligo_db.pl?oligo=A_23_P219072) | SAMD9 | [54809](http://www.ncbi.nih.gov/entrez/query.fcgi?db=gene&cmd=Retrieve&dopt=summary&list_uids=54809) | Hs.65641 | 9.05 | 7.56 | -1.49 | 1 | 0.004 | 2.433 | Homo sapiens sterile alpha motif domain containing 9 (SAMD9), mRNA [NM_017654] |
| [A_23_P129358](http://arrays.ucsf.edu/cgi-bin/oligo_db.pl?oligo=A_23_P129358) | SETD6 | [79918](http://www.ncbi.nih.gov/entrez/query.fcgi?db=gene&cmd=Retrieve&dopt=summary&list_uids=79918) | Hs.592060 | 11.58 | 10.08 | -1.5 | 1 | 0.015 | 0.222 | Homo sapiens SET domain containing 6 (SETD6), mRNA [NM_024860] |
| [A_24_P942335](http://arrays.ucsf.edu/cgi-bin/oligo_db.pl?oligo=A_24_P942335) | C15orf42 | [90381](http://www.ncbi.nih.gov/entrez/query.fcgi?db=gene&cmd=Retrieve&dopt=summary&list_uids=90381) | Hs.441708 | 8.9 | 7.39 | -1.51 | 1 | 0.008 | 1.308 | Homo sapiens chromosome 15 open reading frame 42, mRNA (cDNA clone IMAGE:3940845), partial cds. [BC002881] |
| [A_32_P174385](http://arrays.ucsf.edu/cgi-bin/oligo_db.pl?oligo=A_32_P174385) |  | [NA](../SIX3/NA) |  | 10.88 | 9.35 | -1.53 | 0.841 | 0.003 | 3.244 |  |
| [A_24_P25640](http://arrays.ucsf.edu/cgi-bin/oligo_db.pl?oligo=A_24_P25640) |  | [NA](../SIX3/NA) |  | 10.17 | 8.64 | -1.53 | 1 | 0.008 | 1.311 | Human DF3 breast carcinoma-associated antigen mRNA, partial cds. [J03651] |
| [A_23_P153571](http://arrays.ucsf.edu/cgi-bin/oligo_db.pl?oligo=A_23_P153571) | IGFL2 | [147920](http://www.ncbi.nih.gov/entrez/query.fcgi?db=gene&cmd=Retrieve&dopt=summary&list_uids=147920) | Hs.99376 | 7.81 | 6.29 | -1.53 | 1 | 0.011 | 0.637 | Homo sapiens IGF-like family member 2 (IGFL2), mRNA [NM_001002915] |
| [A_23_P381945](http://arrays.ucsf.edu/cgi-bin/oligo_db.pl?oligo=A_23_P381945) | KRT7 | [3855](http://www.ncbi.nih.gov/entrez/query.fcgi?db=gene&cmd=Retrieve&dopt=summary&list_uids=3855) | Hs.411501 | 13.2 | 11.67 | -1.53 | 0.007 | 0 | 7.803 | Homo sapiens keratin 7 (KRT7), mRNA [NM_005556] |
| [A_23_P160881](http://arrays.ucsf.edu/cgi-bin/oligo_db.pl?oligo=A_23_P160881) | SMPDL3B | [27293](http://www.ncbi.nih.gov/entrez/query.fcgi?db=gene&cmd=Retrieve&dopt=summary&list_uids=27293) | Hs.123659 | 9.9 | 8.37 | -1.53 | 0.774 | 0.003 | 3.326 | Homo sapiens sphingomyelin phosphodiesterase, acid-like 3B (SMPDL3B), transcript variant 2, mRNA [NM_001009568] |
| [A_24_P183544](http://arrays.ucsf.edu/cgi-bin/oligo_db.pl?oligo=A_24_P183544) |  | [NA](../SIX3/NA) |  | 9.29 | 7.76 | -1.54 | 1 | 0.006 | 1.928 | Probable G-protein coupled receptor 110 precursor (G-protein coupled receptor PGR19) (G-protein coupled receptor KPG_012). [Source:Uniprot/SWISSPROT;Acc:Q5T601] [ENST00000371253] |
| [A_23_P159395](http://arrays.ucsf.edu/cgi-bin/oligo_db.pl?oligo=A_23_P159395) | AXIN2 | [8313](http://www.ncbi.nih.gov/entrez/query.fcgi?db=gene&cmd=Retrieve&dopt=summary&list_uids=8313) | Hs.156527 | 8.29 | 6.75 | -1.54 | 1 | 0.005 | 2.102 | Homo sapiens conductin mRNA, complete cds. [AF078165] |
| [A_23_P211488](http://arrays.ucsf.edu/cgi-bin/oligo_db.pl?oligo=A_23_P211488) | APOL2 | [23780](http://www.ncbi.nih.gov/entrez/query.fcgi?db=gene&cmd=Retrieve&dopt=summary&list_uids=23780) | Hs.474740 | 10.65 | 9.1 | -1.55 | 0.257 | 0.001 | 4.408 | Homo sapiens apolipoprotein L, 2 (APOL2), transcript variant beta, mRNA [NM_145637] |
| [A_23_P318604](http://arrays.ucsf.edu/cgi-bin/oligo_db.pl?oligo=A_23_P318604) | CYHR1 | [50626](http://www.ncbi.nih.gov/entrez/query.fcgi?db=gene&cmd=Retrieve&dopt=summary&list_uids=50626) | Hs.459379 | 11.39 | 9.83 | -1.55 | 1 | 0.008 | 1.3 | Homo sapiens cysteine/histidine-rich 1, mRNA (cDNA clone IMAGE:3945559), partial cds. [BC004544] |
| [A_23_P205428](http://arrays.ucsf.edu/cgi-bin/oligo_db.pl?oligo=A_23_P205428) | FOXG1B | [2290](http://www.ncbi.nih.gov/entrez/query.fcgi?db=gene&cmd=Retrieve&dopt=summary&list_uids=2290) | Hs.649934 | 10.93 | 9.37 | -1.56 | 0.082 | 0.001 | 5.506 | Homo sapiens forkhead box G1B (FOXG1B), mRNA [NM_005249] |
| [A_23_P372988](http://arrays.ucsf.edu/cgi-bin/oligo_db.pl?oligo=A_23_P372988) | MGC24975 | [163154](http://www.ncbi.nih.gov/entrez/query.fcgi?db=gene&cmd=Retrieve&dopt=summary&list_uids=163154) | Hs.631838 | 8.12 | 6.56 | -1.56 | 1 | 0.013 | 0.466 | Homo sapiens hypothetical protein MGC24975 (MGC24975), mRNA [NM_153359] |
| [A_23_P218358](http://arrays.ucsf.edu/cgi-bin/oligo_db.pl?oligo=A_23_P218358) | FBXW10 | [10517](http://www.ncbi.nih.gov/entrez/query.fcgi?db=gene&cmd=Retrieve&dopt=summary&list_uids=10517) | Hs.592128 | 8.53 | 6.96 | -1.57 | 1 | 0.013 | 0.414 | Homo sapiens F-box and WD repeat domain containing 10 (FBXW10), mRNA [NM_031456] |
| [A_32_P213002](http://arrays.ucsf.edu/cgi-bin/oligo_db.pl?oligo=A_32_P213002) |  | [NA](../SIX3/NA) | Hs.659222 | 10.41 | 8.83 | -1.58 | 1 | 0.009 | 1.127 | Q504T5_HUMAN (Q504T5) UNC84B protein, partial (12%) [THC2526402] |
| [A_32_P92415](http://arrays.ucsf.edu/cgi-bin/oligo_db.pl?oligo=A_32_P92415) |  | [NA](../SIX3/NA) | Hs.661532 | 9.25 | 7.66 | -1.58 | 0.063 | 0.001 | 5.748 | aa22e03.s1 NCI_CGAP_GCB1 Homo sapiens cDNA clone IMAGE:814012 3', mRNA sequence [AA455656] |
| [A_23_P112482](http://arrays.ucsf.edu/cgi-bin/oligo_db.pl?oligo=A_23_P112482) | AQP3 | [360](http://www.ncbi.nih.gov/entrez/query.fcgi?db=gene&cmd=Retrieve&dopt=summary&list_uids=360) | Hs.234642 | 7.88 | 6.29 | -1.59 | 0.096 | 0.001 | 5.355 | Homo sapiens aquaporin 3 (Gill blood group) (AQP3), mRNA [NM_004925] |
| [A_23_P91802](http://arrays.ucsf.edu/cgi-bin/oligo_db.pl?oligo=A_23_P91802) | ECGF1 | [1890](http://www.ncbi.nih.gov/entrez/query.fcgi?db=gene&cmd=Retrieve&dopt=summary&list_uids=1890) | Hs.592212 | 11.16 | 9.57 | -1.59 | 1 | 0.005 | 2.255 | Homo sapiens endothelial cell growth factor 1 (platelet-derived) (ECGF1), mRNA [NM_001953] |
| [A_23_P51487](http://arrays.ucsf.edu/cgi-bin/oligo_db.pl?oligo=A_23_P51487) | GBP3 | [2635](http://www.ncbi.nih.gov/entrez/query.fcgi?db=gene&cmd=Retrieve&dopt=summary&list_uids=2635) | Hs.656774 | 11.16 | 9.56 | -1.6 | 1 | 0.015 | 0.225 | Homo sapiens guanylate binding protein 3 (GBP3), mRNA [NM_018284] |
| [A_23_P154235](http://arrays.ucsf.edu/cgi-bin/oligo_db.pl?oligo=A_23_P154235) | NMI | [9111](http://www.ncbi.nih.gov/entrez/query.fcgi?db=gene&cmd=Retrieve&dopt=summary&list_uids=9111) | Hs.54483 | 11.66 | 10.06 | -1.6 | 1 | 0.008 | 1.195 | Homo sapiens N-myc (and STAT) interactor (NMI), mRNA [NM_004688] |
| [A_24_P370702](http://arrays.ucsf.edu/cgi-bin/oligo_db.pl?oligo=A_24_P370702) | GBP3 | [2635](http://www.ncbi.nih.gov/entrez/query.fcgi?db=gene&cmd=Retrieve&dopt=summary&list_uids=2635) | Hs.656774 | 11.82 | 10.21 | -1.6 | 1 | 0.009 | 1.029 | Homo sapiens guanylate binding protein 3 (GBP3), mRNA [NM_018284] |
| [A_24_P228302](http://arrays.ucsf.edu/cgi-bin/oligo_db.pl?oligo=A_24_P228302) | CEACAM7 | [1087](http://www.ncbi.nih.gov/entrez/query.fcgi?db=gene&cmd=Retrieve&dopt=summary&list_uids=1087) | Hs.74466 | 8.51 | 6.9 | -1.61 | 1 | 0.003 | 2.921 | Homo sapiens carcinoembryonic antigen-related cell adhesion molecule 7 (CEACAM7), mRNA [NM_006890] |
| [A_24_P166397](http://arrays.ucsf.edu/cgi-bin/oligo_db.pl?oligo=A_24_P166397) | KIAA0319 | [9856](http://www.ncbi.nih.gov/entrez/query.fcgi?db=gene&cmd=Retrieve&dopt=summary&list_uids=9856) | Hs.26441 | 9.86 | 8.23 | -1.63 | 0.447 | 0.002 | 3.867 | Homo sapiens KIAA0319 (KIAA0319), mRNA [NM_014809] |
| [A_23_P164451](http://arrays.ucsf.edu/cgi-bin/oligo_db.pl?oligo=A_23_P164451) | TBX2 | [6909](http://www.ncbi.nih.gov/entrez/query.fcgi?db=gene&cmd=Retrieve&dopt=summary&list_uids=6909) | Hs.693658 | 10.37 | 8.74 | -1.63 | 1 | 0.005 | 2.24 | Homo sapiens T-box 2 (TBX2), mRNA [NM_005994] |
| [A_23_P252541](http://arrays.ucsf.edu/cgi-bin/oligo_db.pl?oligo=A_23_P252541) | RAB7B | [338382](http://www.ncbi.nih.gov/entrez/query.fcgi?db=gene&cmd=Retrieve&dopt=summary&list_uids=338382) | Hs.534612 | 9.58 | 7.94 | -1.64 | 0.796 | 0.003 | 3.298 | Homo sapiens RAB7B, member RAS oncogene family (RAB7B), mRNA [NM_177403] |
| [A_24_P348326](http://arrays.ucsf.edu/cgi-bin/oligo_db.pl?oligo=A_24_P348326) | IL27RA | [9466](http://www.ncbi.nih.gov/entrez/query.fcgi?db=gene&cmd=Retrieve&dopt=summary&list_uids=9466) | Hs.132781 | 13.09 | 11.43 | -1.66 | 1 | 0.004 | 2.607 | Homo sapiens interleukin 27 receptor, alpha (IL27RA), mRNA [NM_004843] |
| [A_23_P309198](http://arrays.ucsf.edu/cgi-bin/oligo_db.pl?oligo=A_23_P309198) | C21orf69 | [84537](http://www.ncbi.nih.gov/entrez/query.fcgi?db=gene&cmd=Retrieve&dopt=summary&list_uids=84537) | Hs.534504 | 9.55 | 7.88 | -1.66 | 1 | 0.009 | 0.961 | Homo sapiens chromosome 21 open reading frame 69 (C21orf69), mRNA [NM_058189] |
| [A_24_P315056](http://arrays.ucsf.edu/cgi-bin/oligo_db.pl?oligo=A_24_P315056) | C10orf122 | [387718](http://www.ncbi.nih.gov/entrez/query.fcgi?db=gene&cmd=Retrieve&dopt=summary&list_uids=387718) | Hs.148259 | 9.23 | 7.56 | -1.67 | 0.317 | 0.002 | 4.202 | Homo sapiens chromosome 10 open reading frame 122, mRNA (cDNA clone MGC:72014 IMAGE:6101709), complete cds. [BC062717] |
| [A_32_P12327](http://arrays.ucsf.edu/cgi-bin/oligo_db.pl?oligo=A_32_P12327) | LOC643783 | [643783](http://www.ncbi.nih.gov/entrez/query.fcgi?db=gene&cmd=Retrieve&dopt=summary&list_uids=643783) | Hs.631514 | 8.98 | 7.3 | -1.68 | 0.026 | 0.001 | 6.567 | PREDICTED: Homo sapiens hypothetical LOC643783 (LOC643783), mRNA [XM_931798] |
| [A_24_P175187](http://arrays.ucsf.edu/cgi-bin/oligo_db.pl?oligo=A_24_P175187) | SAMD9 | [54809](http://www.ncbi.nih.gov/entrez/query.fcgi?db=gene&cmd=Retrieve&dopt=summary&list_uids=54809) | Hs.65641 | 9.93 | 8.25 | -1.68 | 1 | 0.009 | 0.964 | Homo sapiens sterile alpha motif domain containing 9 (SAMD9), mRNA [NM_017654] |
| [A_23_P23669](http://arrays.ucsf.edu/cgi-bin/oligo_db.pl?oligo=A_23_P23669) | PALMD | [54873](http://www.ncbi.nih.gov/entrez/query.fcgi?db=gene&cmd=Retrieve&dopt=summary&list_uids=54873) | Hs.483993 | 7.94 | 6.24 | -1.7 | 1 | 0.005 | 2.284 | Homo sapiens palmdelphin (PALMD), mRNA [NM_017734] |
| [A_23_P11408](http://arrays.ucsf.edu/cgi-bin/oligo_db.pl?oligo=A_23_P11408) | PRY2 | [442862](http://www.ncbi.nih.gov/entrez/query.fcgi?db=gene&cmd=Retrieve&dopt=summary&list_uids=442862) | Hs.632847 | 8.76 | 7.04 | -1.72 | 0.384 | 0.002 | 4.016 | Homo sapiens PTPN13-like, Y-linked 2 (PRY2), mRNA [NM_001002758] |
| [A_23_P20814](http://arrays.ucsf.edu/cgi-bin/oligo_db.pl?oligo=A_23_P20814) | DDX58 | [23586](http://www.ncbi.nih.gov/entrez/query.fcgi?db=gene&cmd=Retrieve&dopt=summary&list_uids=23586) | Hs.190622 | 14.34 | 12.62 | -1.72 | 1 | 0.013 | 0.465 | Homo sapiens DEAD (Asp-Glu-Ala-Asp) box polypeptide 58 (DDX58), mRNA [NM_014314] |
| [A_23_P337800](http://arrays.ucsf.edu/cgi-bin/oligo_db.pl?oligo=A_23_P337800) | IL29 | [282618](http://www.ncbi.nih.gov/entrez/query.fcgi?db=gene&cmd=Retrieve&dopt=summary&list_uids=282618) | Hs.406745 | 11.42 | 9.68 | -1.74 | 1 | 0.007 | 1.503 | Homo sapiens interleukin 29 (interferon, lambda 1) (IL29), mRNA [NM_172140] |
| [A_23_P10182](http://arrays.ucsf.edu/cgi-bin/oligo_db.pl?oligo=A_23_P10182) | ACOX2 | [8309](http://www.ncbi.nih.gov/entrez/query.fcgi?db=gene&cmd=Retrieve&dopt=summary&list_uids=8309) | Hs.444959 | 10.08 | 8.32 | -1.76 | 1 | 0.005 | 1.945 | Homo sapiens acyl-Coenzyme A oxidase 2, branched chain (ACOX2), mRNA [NM_003500] |
| [A_23_P168324](http://arrays.ucsf.edu/cgi-bin/oligo_db.pl?oligo=A_23_P168324) | KIAA0319 | [9856](http://www.ncbi.nih.gov/entrez/query.fcgi?db=gene&cmd=Retrieve&dopt=summary&list_uids=9856) | Hs.26441 | 12.46 | 10.7 | -1.76 | 1 | 0.004 | 2.354 | Homo sapiens KIAA0319 (KIAA0319), mRNA [NM_014809] |
| [A_23_P502879](http://arrays.ucsf.edu/cgi-bin/oligo_db.pl?oligo=A_23_P502879) | EDG2 | [1902](http://www.ncbi.nih.gov/entrez/query.fcgi?db=gene&cmd=Retrieve&dopt=summary&list_uids=1902) | Hs.126667 | 8.74 | 6.98 | -1.76 | 0.229 | 0.001 | 4.52 | Homo sapiens endothelial differentiation, lysophosphatidic acid G-protein-coupled receptor, 2 (EDG2), transcript variant 2, mRNA [NM_057159] |
| [A_23_P132515](http://arrays.ucsf.edu/cgi-bin/oligo_db.pl?oligo=A_23_P132515) | SIDT1 | [54847](http://www.ncbi.nih.gov/entrez/query.fcgi?db=gene&cmd=Retrieve&dopt=summary&list_uids=54847) | Hs.591291 | 8.35 | 6.58 | -1.77 | 0.05 | 0.001 | 5.973 | Homo sapiens SID1 transmembrane family, member 1 (SIDT1), mRNA [NM_017699] |
| [A_23_P358709](http://arrays.ucsf.edu/cgi-bin/oligo_db.pl?oligo=A_23_P358709) | AHRR | [57491](http://www.ncbi.nih.gov/entrez/query.fcgi?db=gene&cmd=Retrieve&dopt=summary&list_uids=57491) | Hs.50823 | 9.08 | 7.3 | -1.78 | 1 | 0.006 | 1.776 | Homo sapiens aryl-hydrocarbon receptor repressor (AHRR), mRNA [NM_020731] |
| [A_24_P804667](http://arrays.ucsf.edu/cgi-bin/oligo_db.pl?oligo=A_24_P804667) | LOC751071 | [751071](http://www.ncbi.nih.gov/entrez/query.fcgi?db=gene&cmd=Retrieve&dopt=summary&list_uids=751071) | Hs.640726 | 10.19 | 8.41 | -1.78 | 0.036 | 0.001 | 6.285 | Homo sapiens hypothetical protein LOC751071 (LOC751071), mRNA [NM_001043229] |
| [A_23_P204654](http://arrays.ucsf.edu/cgi-bin/oligo_db.pl?oligo=A_23_P204654) | KITLG | [4254](http://www.ncbi.nih.gov/entrez/query.fcgi?db=gene&cmd=Retrieve&dopt=summary&list_uids=4254) | Hs.1048 | 9.02 | 7.24 | -1.78 | 1 | 0.007 | 1.573 | Homo sapiens KIT ligand (KITLG), transcript variant b, mRNA [NM_000899] |
| [A_23_P29237](http://arrays.ucsf.edu/cgi-bin/oligo_db.pl?oligo=A_23_P29237) | APOL3 | [80833](http://www.ncbi.nih.gov/entrez/query.fcgi?db=gene&cmd=Retrieve&dopt=summary&list_uids=80833) | Hs.474737 | 8.46 | 6.67 | -1.79 | 1 | 0.006 | 1.85 | Homo sapiens apolipoprotein L, 3 (APOL3), transcript variant beta/a, mRNA [NM_145641] |
| [A_24_P175188](http://arrays.ucsf.edu/cgi-bin/oligo_db.pl?oligo=A_24_P175188) | SAMD9 | [54809](http://www.ncbi.nih.gov/entrez/query.fcgi?db=gene&cmd=Retrieve&dopt=summary&list_uids=54809) | Hs.65641 | 10.58 | 8.79 | -1.79 | 1 | 0.009 | 1.131 | Homo sapiens sterile alpha motif domain containing 9 (SAMD9), mRNA [NM_017654] |
| [A_23_P130515](http://arrays.ucsf.edu/cgi-bin/oligo_db.pl?oligo=A_23_P130515) | CEACAM3 | [1084](http://www.ncbi.nih.gov/entrez/query.fcgi?db=gene&cmd=Retrieve&dopt=summary&list_uids=1084) | Hs.11 | 8.33 | 6.53 | -1.8 | 1 | 0.005 | 2.182 | Homo sapiens carcinoembryonic antigen-related cell adhesion molecule 3 (CEACAM3), mRNA [NM_001815] |
| [A_23_P98399](http://arrays.ucsf.edu/cgi-bin/oligo_db.pl?oligo=A_23_P98399) | HTR3A | [3359](http://www.ncbi.nih.gov/entrez/query.fcgi?db=gene&cmd=Retrieve&dopt=summary&list_uids=3359) | Hs.413899 | 8.75 | 6.94 | -1.8 | 0.677 | 0.002 | 3.458 | Homo sapiens 5-hydroxytryptamine (serotonin) receptor 3A (HTR3A), transcript variant 1, mRNA [NM_213621] |
| [A_23_P355244](http://arrays.ucsf.edu/cgi-bin/oligo_db.pl?oligo=A_23_P355244) | SAMD9 | [54809](http://www.ncbi.nih.gov/entrez/query.fcgi?db=gene&cmd=Retrieve&dopt=summary&list_uids=54809) | Hs.65641 | 13.91 | 12.09 | -1.83 | 1 | 0.012 | 0.556 | Homo sapiens sterile alpha motif domain containing 9 (SAMD9), mRNA [NM_017654] |
| [A_23_P408955](http://arrays.ucsf.edu/cgi-bin/oligo_db.pl?oligo=A_23_P408955) | E2F2 | [1870](http://www.ncbi.nih.gov/entrez/query.fcgi?db=gene&cmd=Retrieve&dopt=summary&list_uids=1870) | Hs.194333 | 13.2 | 11.36 | -1.84 | 1 | 0.004 | 2.751 | Homo sapiens E2F transcription factor 2 (E2F2), mRNA [NM_004091] |
| [A_24_P341938](http://arrays.ucsf.edu/cgi-bin/oligo_db.pl?oligo=A_24_P341938) | ZC3HAV1 | [56829](http://www.ncbi.nih.gov/entrez/query.fcgi?db=gene&cmd=Retrieve&dopt=summary&list_uids=56829) | Hs.133512 | 9.9 | 8.06 | -1.84 | 0.038 | 0.001 | 6.211 | Homo sapiens zinc finger CCCH-type, antiviral 1 (ZC3HAV1), transcript variant 2, mRNA [NM_024625] |
| [A_23_P347040](http://arrays.ucsf.edu/cgi-bin/oligo_db.pl?oligo=A_23_P347040) | DTX3L | [151636](http://www.ncbi.nih.gov/entrez/query.fcgi?db=gene&cmd=Retrieve&dopt=summary&list_uids=151636) | Hs.518201 | 8.79 | 6.93 | -1.86 | 0.115 | 0.001 | 5.178 | Homo sapiens deltex 3-like (Drosophila) (DTX3L), mRNA [NM_138287] |
| [A_23_P393620](http://arrays.ucsf.edu/cgi-bin/oligo_db.pl?oligo=A_23_P393620) | TFPI2 | [7980](http://www.ncbi.nih.gov/entrez/query.fcgi?db=gene&cmd=Retrieve&dopt=summary&list_uids=7980) | Hs.438231 | 12.63 | 10.75 | -1.88 | 1 | 0.01 | 0.8 | Homo sapiens tissue factor pathway inhibitor 2 (TFPI2), mRNA [NM_006528] |
| [A_24_P153456](http://arrays.ucsf.edu/cgi-bin/oligo_db.pl?oligo=A_24_P153456) | ZDHHC11 | [79844](http://www.ncbi.nih.gov/entrez/query.fcgi?db=gene&cmd=Retrieve&dopt=summary&list_uids=79844) | Hs.659832 | 10.48 | 8.59 | -1.89 | 0.442 | 0.002 | 3.878 | Homo sapiens zinc finger, DHHC-type containing 11 (ZDHHC11), mRNA [NM_024786] |
| [A_23_P380857](http://arrays.ucsf.edu/cgi-bin/oligo_db.pl?oligo=A_23_P380857) | APOL4 | [80832](http://www.ncbi.nih.gov/entrez/query.fcgi?db=gene&cmd=Retrieve&dopt=summary&list_uids=80832) | Hs.115099 | 9.45 | 7.55 | -1.9 | 1 | 0.017 | 0.008 | Homo sapiens apolipoprotein L, 4 (APOL4), transcript variant a, mRNA [NM_030643] |
| [A_23_P67360](http://arrays.ucsf.edu/cgi-bin/oligo_db.pl?oligo=A_23_P67360) | PLEKHA4 | [57664](http://www.ncbi.nih.gov/entrez/query.fcgi?db=gene&cmd=Retrieve&dopt=summary&list_uids=57664) | Hs.9469 | 8.7 | 6.79 | -1.91 | 0.022 | 0 | 6.728 | Homo sapiens pleckstrin homology domain containing, family A (phosphoinositide binding specific) member 4 (PLEKHA4), mRNA [NM_020904] |
| [A_23_P819](http://arrays.ucsf.edu/cgi-bin/oligo_db.pl?oligo=A_23_P819) | ISG15 | [9636](http://www.ncbi.nih.gov/entrez/query.fcgi?db=gene&cmd=Retrieve&dopt=summary&list_uids=9636) | Hs.458485 | 17.91 | 15.97 | -1.94 | 0.373 | 0.002 | 4.043 | Homo sapiens ISG15 ubiquitin-like modifier (ISG15), mRNA [NM_005101] |
| [A_24_P61490](http://arrays.ucsf.edu/cgi-bin/oligo_db.pl?oligo=A_24_P61490) | TITF1 | [7080](http://www.ncbi.nih.gov/entrez/query.fcgi?db=gene&cmd=Retrieve&dopt=summary&list_uids=7080) | Hs.94367 | 12.55 | 10.61 | -1.94 | 1 | 0.009 | 1.129 | Homo sapiens thyroid transcription factor 1 (TITF1), transcript variant 2, mRNA [NM_003317] |
| [A_23_P256008](http://arrays.ucsf.edu/cgi-bin/oligo_db.pl?oligo=A_23_P256008) | ZDHHC11 | [79844](http://www.ncbi.nih.gov/entrez/query.fcgi?db=gene&cmd=Retrieve&dopt=summary&list_uids=79844) | Hs.659832 | 11.86 | 9.91 | -1.95 | 0.37 | 0.002 | 4.052 | Homo sapiens zinc finger, DHHC-type containing 11 (ZDHHC11), mRNA [NM_024786] |
| [A_23_P60120](http://arrays.ucsf.edu/cgi-bin/oligo_db.pl?oligo=A_23_P60120) | MLZE | [56169](http://www.ncbi.nih.gov/entrez/query.fcgi?db=gene&cmd=Retrieve&dopt=summary&list_uids=56169) | Hs.133244 | 10.47 | 8.52 | -1.95 | 1 | 0.016 | 0.086 | Homo sapiens melanoma-derived leucine zipper, extra-nuclear factor (MLZE), mRNA [NM_031415] |
| [A_24_P30194](http://arrays.ucsf.edu/cgi-bin/oligo_db.pl?oligo=A_24_P30194) | IFIT5 | [24138](http://www.ncbi.nih.gov/entrez/query.fcgi?db=gene&cmd=Retrieve&dopt=summary&list_uids=24138) | Hs.252839 | 11.81 | 9.86 | -1.96 | 0.368 | 0.002 | 4.058 | Homo sapiens interferon-induced protein with tetratricopeptide repeats 5 (IFIT5), mRNA [NM_012420] |
| [A_23_P137381](http://arrays.ucsf.edu/cgi-bin/oligo_db.pl?oligo=A_23_P137381) | ID3 | [3399](http://www.ncbi.nih.gov/entrez/query.fcgi?db=gene&cmd=Retrieve&dopt=summary&list_uids=3399) | Hs.76884 | 13.46 | 11.5 | -1.96 | 0.002 | 0 | 8.83 | Homo sapiens inhibitor of DNA binding 3, dominant negative helix-loop-helix protein (ID3), mRNA [NM_002167] |
| [A_24_P926960](http://arrays.ucsf.edu/cgi-bin/oligo_db.pl?oligo=A_24_P926960) | MEGF6 | [1953](http://www.ncbi.nih.gov/entrez/query.fcgi?db=gene&cmd=Retrieve&dopt=summary&list_uids=1953) | Hs.593645 | 10.29 | 8.32 | -1.96 | 1 | 0.005 | 2.077 | Homo sapiens multiple EGF-like-domains 6 (MEGF6), mRNA [NM_001409] |
| [A_23_P120002](http://arrays.ucsf.edu/cgi-bin/oligo_db.pl?oligo=A_23_P120002) | SP110 | [3431](http://www.ncbi.nih.gov/entrez/query.fcgi?db=gene&cmd=Retrieve&dopt=summary&list_uids=3431) | Hs.145150 | 11.78 | 9.8 | -1.98 | 0.143 | 0.001 | 4.97 | Homo sapiens SP110 nuclear body protein (SP110), transcript variant b, mRNA [NM_004510] |
| [A_32_P182395](http://arrays.ucsf.edu/cgi-bin/oligo_db.pl?oligo=A_32_P182395) |  | [NA](../SIX3/NA) |  | 11.13 | 9.14 | -1.98 | 0.3 | 0.002 | 4.257 | Q8WUP8_HUMAN (Q8WUP8) MGC21881 protein (Fragment), partial (36%) [THC2534222] |
| [A_23_P72127](http://arrays.ucsf.edu/cgi-bin/oligo_db.pl?oligo=A_23_P72127) | KIAA1446 | [57596](http://www.ncbi.nih.gov/entrez/query.fcgi?db=gene&cmd=Retrieve&dopt=summary&list_uids=57596) | Hs.211751 | 12.06 | 10.07 | -1.99 | 1 | 0.004 | 2.546 | Homo sapiens likely ortholog of rat brain-enriched guanylate kinase-associated protein (KIAA1446), mRNA [NM_020836] |
| [A_23_P145874](http://arrays.ucsf.edu/cgi-bin/oligo_db.pl?oligo=A_23_P145874) | SAMD9L | [219285](http://www.ncbi.nih.gov/entrez/query.fcgi?db=gene&cmd=Retrieve&dopt=summary&list_uids=219285) | Hs.489118 | 8.48 | 6.45 | -2.03 | 1 | 0.005 | 2.273 | Homo sapiens sterile alpha motif domain containing 9-like (SAMD9L), mRNA [NM_152703] |
| [A_23_P58266](http://arrays.ucsf.edu/cgi-bin/oligo_db.pl?oligo=A_23_P58266) | S100P | [6286](http://www.ncbi.nih.gov/entrez/query.fcgi?db=gene&cmd=Retrieve&dopt=summary&list_uids=6286) | Hs.2962 | 14.19 | 12.14 | -2.05 | 0.364 | 0.002 | 4.069 | Homo sapiens S100 calcium binding protein P (S100P), mRNA [NM_005980] |
| [A_23_P253661](http://arrays.ucsf.edu/cgi-bin/oligo_db.pl?oligo=A_23_P253661) | FLJ13236 | [79962](http://www.ncbi.nih.gov/entrez/query.fcgi?db=gene&cmd=Retrieve&dopt=summary&list_uids=79962) | Hs.659300 | 9.76 | 7.68 | -2.08 | 0.123 | 0.001 | 5.12 | Homo sapiens hypothetical protein FLJ13236 (FLJ13236), mRNA [NM_024902] |
| [A_24_P941912](http://arrays.ucsf.edu/cgi-bin/oligo_db.pl?oligo=A_24_P941912) | DTX3L | [151636](http://www.ncbi.nih.gov/entrez/query.fcgi?db=gene&cmd=Retrieve&dopt=summary&list_uids=151636) | Hs.518201 | 9.18 | 7.04 | -2.14 | 0.231 | 0.001 | 4.512 | Homo sapiens deltex 3-like (Drosophila) (DTX3L), mRNA [NM_138287] |
| [A_24_P56310](http://arrays.ucsf.edu/cgi-bin/oligo_db.pl?oligo=A_24_P56310) | TNFRSF19 | [55504](http://www.ncbi.nih.gov/entrez/query.fcgi?db=gene&cmd=Retrieve&dopt=summary&list_uids=55504) | Hs.149168 | 8.17 | 6.04 | -2.14 | 0 | 0 | 10.414 | Homo sapiens tumor necrosis factor receptor superfamily, member 19 (TNFRSF19), transcript variant 2, mRNA [NM_148957] |
| [A_24_P48898](http://arrays.ucsf.edu/cgi-bin/oligo_db.pl?oligo=A_24_P48898) | APOL2 | [23780](http://www.ncbi.nih.gov/entrez/query.fcgi?db=gene&cmd=Retrieve&dopt=summary&list_uids=23780) | Hs.474740 | 13.56 | 11.41 | -2.15 | 1 | 0.007 | 1.402 | Homo sapiens apolipoprotein L, 2 (APOL2), transcript variant beta, mRNA [NM_145637] |
| [A_23_P55738](http://arrays.ucsf.edu/cgi-bin/oligo_db.pl?oligo=A_23_P55738) | CEACAM1 | [634](http://www.ncbi.nih.gov/entrez/query.fcgi?db=gene&cmd=Retrieve&dopt=summary&list_uids=634) | Hs.512682 | 10.51 | 8.35 | -2.16 | 1 | 0.012 | 0.57 | Homo sapiens carcinoembryonic antigen-related cell adhesion molecule 1 (biliary glycoprotein) (CEACAM1), transcript variant 1, mRNA [NM_001712] |
| [A_23_P17065](http://arrays.ucsf.edu/cgi-bin/oligo_db.pl?oligo=A_23_P17065) | CCL20 | [6364](http://www.ncbi.nih.gov/entrez/query.fcgi?db=gene&cmd=Retrieve&dopt=summary&list_uids=6364) | Hs.75498 | 8.73 | 6.57 | -2.16 | 1 | 0.004 | 2.325 | Homo sapiens chemokine (C-C motif) ligand 20 (CCL20), mRNA [NM_004591] |
| [A_23_P59375](http://arrays.ucsf.edu/cgi-bin/oligo_db.pl?oligo=A_23_P59375) | ID4 | [3400](http://www.ncbi.nih.gov/entrez/query.fcgi?db=gene&cmd=Retrieve&dopt=summary&list_uids=3400) | Hs.519601 | 11.46 | 9.29 | -2.17 | 1 | 0.005 | 2.084 | Homo sapiens inhibitor of DNA binding 4, dominant negative helix-loop-helix protein (ID4), mRNA [NM_001546] |
| [A_23_P74012](http://arrays.ucsf.edu/cgi-bin/oligo_db.pl?oligo=A_23_P74012) | SPRR1A | [6698](http://www.ncbi.nih.gov/entrez/query.fcgi?db=gene&cmd=Retrieve&dopt=summary&list_uids=6698) | Hs.46320 | 10.36 | 8.19 | -2.18 | 1 | 0.007 | 1.484 | Homo sapiens small proline-rich protein 1A (SPRR1A), mRNA [NM_005987] |
| [A_24_P38815](http://arrays.ucsf.edu/cgi-bin/oligo_db.pl?oligo=A_24_P38815) | TPP1 | [1200](http://www.ncbi.nih.gov/entrez/query.fcgi?db=gene&cmd=Retrieve&dopt=summary&list_uids=1200) | Hs.523454 | 8.57 | 6.4 | -2.18 | 1 | 0.006 | 1.812 | Homo sapiens tripeptidyl peptidase I (TPP1), mRNA [NM_000391] |
| [A_24_P183150](http://arrays.ucsf.edu/cgi-bin/oligo_db.pl?oligo=A_24_P183150) | CXCL3 | [2921](http://www.ncbi.nih.gov/entrez/query.fcgi?db=gene&cmd=Retrieve&dopt=summary&list_uids=2921) | Hs.89690 | 10.03 | 7.82 | -2.21 | 1 | 0.007 | 1.399 | Homo sapiens chemokine (C-X-C motif) ligand 3 (CXCL3), mRNA [NM_002090] |
| [A_32_P171793](http://arrays.ucsf.edu/cgi-bin/oligo_db.pl?oligo=A_32_P171793) |  | [NA](../SIX3/NA) |  | 9.06 | 6.82 | -2.23 | 0.009 | 0 | 7.517 |  |
| [A_24_P161018](http://arrays.ucsf.edu/cgi-bin/oligo_db.pl?oligo=A_24_P161018) | PARP14 | [54625](http://www.ncbi.nih.gov/entrez/query.fcgi?db=gene&cmd=Retrieve&dopt=summary&list_uids=54625) | Hs.518203 | 10.82 | 8.58 | -2.24 | 0.465 | 0.002 | 3.827 | Homo sapiens poly (ADP-ribose) polymerase family, member 14 (PARP14), mRNA [NM_017554] |
| [A_23_P110196](http://arrays.ucsf.edu/cgi-bin/oligo_db.pl?oligo=A_23_P110196) | HERC5 | [51191](http://www.ncbi.nih.gov/entrez/query.fcgi?db=gene&cmd=Retrieve&dopt=summary&list_uids=51191) | Hs.26663 | 11.66 | 9.32 | -2.33 | 0.656 | 0.002 | 3.49 | Homo sapiens hect domain and RLD 5 (HERC5), mRNA [NM_016323] |
| [A_23_P112481](http://arrays.ucsf.edu/cgi-bin/oligo_db.pl?oligo=A_23_P112481) | AQP3 | [360](http://www.ncbi.nih.gov/entrez/query.fcgi?db=gene&cmd=Retrieve&dopt=summary&list_uids=360) | Hs.234642 | 9.15 | 6.78 | -2.37 | 0.885 | 0.003 | 3.193 | Homo sapiens aquaporin 3 (Gill blood group) (AQP3), mRNA [NM_004925] |
| [A_23_P29922](http://arrays.ucsf.edu/cgi-bin/oligo_db.pl?oligo=A_23_P29922) | TLR3 | [7098](http://www.ncbi.nih.gov/entrez/query.fcgi?db=gene&cmd=Retrieve&dopt=summary&list_uids=7098) | Hs.657724 | 8.72 | 6.33 | -2.39 | 0.006 | 0 | 7.885 | Homo sapiens toll-like receptor 3 (TLR3), mRNA [NM_003265] |
| [A_23_P215549](http://arrays.ucsf.edu/cgi-bin/oligo_db.pl?oligo=A_23_P215549) | PON3 | [5446](http://www.ncbi.nih.gov/entrez/query.fcgi?db=gene&cmd=Retrieve&dopt=summary&list_uids=5446) | Hs.440967 | 12.68 | 10.29 | -2.39 | 0.311 | 0.002 | 4.221 | Homo sapiens paraoxonase 3 (PON3), mRNA [NM_000940] |
| [A_24_P342096](http://arrays.ucsf.edu/cgi-bin/oligo_db.pl?oligo=A_24_P342096) |  | [NA](../SIX3/NA) | Hs.645763 | 12.44 | 10.05 | -2.39 | 0.218 | 0.001 | 4.567 | Homo sapiens cDNA clone IMAGE:6018774, partial cds. [BC073976] |
| [A_24_P916547](http://arrays.ucsf.edu/cgi-bin/oligo_db.pl?oligo=A_24_P916547) |  | [NA](../SIX3/NA) |  | 12.55 | 10.15 | -2.4 | 0.114 | 0.001 | 5.185 | Q5VT28_HUMAN (Q5VT28) Family with sequence similarity 27, member B (Family with sequence similarity 27, member A) (Family with sequence similarity 27, member C), partial (81%) [THC2565393] |
| [A_24_P365721](http://arrays.ucsf.edu/cgi-bin/oligo_db.pl?oligo=A_24_P365721) | SLC6A14 | [11254](http://www.ncbi.nih.gov/entrez/query.fcgi?db=gene&cmd=Retrieve&dopt=summary&list_uids=11254) | Hs.522109 | 8.58 | 6.16 | -2.42 | 0.001 | 0 | 9.431 | Homo sapiens solute carrier family 6 (amino acid transporter), member 14 (SLC6A14), mRNA [NM_007231] |
| [A_24_P287043](http://arrays.ucsf.edu/cgi-bin/oligo_db.pl?oligo=A_24_P287043) | IFITM2 | [10581](http://www.ncbi.nih.gov/entrez/query.fcgi?db=gene&cmd=Retrieve&dopt=summary&list_uids=10581) | Hs.174195 | 15.63 | 13.2 | -2.43 | 1 | 0.005 | 2.04 | Homo sapiens interferon induced transmembrane protein 2 (1-8D) (IFITM2), mRNA [NM_006435] |
| [A_23_P24004](http://arrays.ucsf.edu/cgi-bin/oligo_db.pl?oligo=A_23_P24004) | IFIT2 | [3433](http://www.ncbi.nih.gov/entrez/query.fcgi?db=gene&cmd=Retrieve&dopt=summary&list_uids=3433) | Hs.437609 | 16.02 | 13.59 | -2.43 | 1 | 0.006 | 1.863 | Homo sapiens interferon-induced protein with tetratricopeptide repeats 2 (IFIT2), mRNA [NM_001547] |
| [A_23_P160025](http://arrays.ucsf.edu/cgi-bin/oligo_db.pl?oligo=A_23_P160025) | IFI16 | [3428](http://www.ncbi.nih.gov/entrez/query.fcgi?db=gene&cmd=Retrieve&dopt=summary&list_uids=3428) | Hs.380250 | 9.31 | 6.87 | -2.44 | 1 | 0.006 | 1.828 | Homo sapiens interferon, gamma-inducible protein 16 (IFI16), mRNA [NM_005531] |
| [A_32_P180265](http://arrays.ucsf.edu/cgi-bin/oligo_db.pl?oligo=A_32_P180265) |  | [NA](../SIX3/NA) |  | 11.21 | 8.77 | -2.44 | 0.15 | 0.001 | 4.927 | Q5VT28_HUMAN (Q5VT28) Family with sequence similarity 27, member B (Family with sequence similarity 27, member A) (Family with sequence similarity 27, member C), partial (85%) [THC2538882] |
| [A_23_P17837](http://arrays.ucsf.edu/cgi-bin/oligo_db.pl?oligo=A_23_P17837) | APOL1 | [8542](http://www.ncbi.nih.gov/entrez/query.fcgi?db=gene&cmd=Retrieve&dopt=summary&list_uids=8542) | Hs.114309 | 9.47 | 7.03 | -2.44 | 0.754 | 0.003 | 3.352 | Homo sapiens apolipoprotein L, 1 (APOL1), transcript variant 2, mRNA [NM_145343] |
| [A_23_P131208](http://arrays.ucsf.edu/cgi-bin/oligo_db.pl?oligo=A_23_P131208) | NR4A2 | [4929](http://www.ncbi.nih.gov/entrez/query.fcgi?db=gene&cmd=Retrieve&dopt=summary&list_uids=4929) | Hs.563344 | 10.4 | 7.96 | -2.45 | 0.377 | 0.002 | 4.034 | Homo sapiens nuclear receptor subfamily 4, group A, member 2 (NR4A2), transcript variant 1, mRNA [NM_006186] |
| [A_23_P121253](http://arrays.ucsf.edu/cgi-bin/oligo_db.pl?oligo=A_23_P121253) | TNFSF10 | [8743](http://www.ncbi.nih.gov/entrez/query.fcgi?db=gene&cmd=Retrieve&dopt=summary&list_uids=8743) | Hs.478275 | 10.15 | 7.68 | -2.48 | 1 | 0.016 | 0.106 | Homo sapiens tumor necrosis factor (ligand) superfamily, member 10 (TNFSF10), mRNA [NM_003810] |
| [A_24_P303091](http://arrays.ucsf.edu/cgi-bin/oligo_db.pl?oligo=A_24_P303091) | CXCL10 | [3627](http://www.ncbi.nih.gov/entrez/query.fcgi?db=gene&cmd=Retrieve&dopt=summary&list_uids=3627) | Hs.632586 | 11.06 | 8.58 | -2.49 | 1 | 0.007 | 1.411 | Homo sapiens chemokine (C-X-C motif) ligand 10 (CXCL10), mRNA [NM_001565] |
| [A_23_P434118](http://arrays.ucsf.edu/cgi-bin/oligo_db.pl?oligo=A_23_P434118) | CEACAM1 | [634](http://www.ncbi.nih.gov/entrez/query.fcgi?db=gene&cmd=Retrieve&dopt=summary&list_uids=634) | Hs.512682 | 11.14 | 8.65 | -2.5 | 1 | 0.01 | 0.888 | Homo sapiens carcinoembryonic antigen-related cell adhesion molecule 1 (biliary glycoprotein) (CEACAM1), transcript variant 2, mRNA [NM_001024912] |
| [A_32_P17525](http://arrays.ucsf.edu/cgi-bin/oligo_db.pl?oligo=A_32_P17525) |  | [NA](../SIX3/NA) |  | 9.18 | 6.67 | -2.51 | 1 | 0.01 | 0.928 | Q9SI74_ARATH (Q9SI74) F23N19.12, partial (5%) [THC2664860] |
| [A_23_P148015](http://arrays.ucsf.edu/cgi-bin/oligo_db.pl?oligo=A_23_P148015) | AXIN2 | [8313](http://www.ncbi.nih.gov/entrez/query.fcgi?db=gene&cmd=Retrieve&dopt=summary&list_uids=8313) | Hs.156527 | 11.04 | 8.5 | -2.55 | 0.023 | 0.001 | 6.665 | Homo sapiens axin 2 (conductin, axil) (AXIN2), mRNA [NM_004655] |
| [A_24_P58620](http://arrays.ucsf.edu/cgi-bin/oligo_db.pl?oligo=A_24_P58620) | GRAMD1B | [57476](http://www.ncbi.nih.gov/entrez/query.fcgi?db=gene&cmd=Retrieve&dopt=summary&list_uids=57476) | Hs.144725 | 12.49 | 9.94 | -2.55 | 0.055 | 0.001 | 5.874 | Homo sapiens clone DNA132162 PTSS3032 (UNQ3032) mRNA, complete cds. [AY358924] |
| [A_23_P20122](http://arrays.ucsf.edu/cgi-bin/oligo_db.pl?oligo=A_23_P20122) | ZC3HAV1 | [56829](http://www.ncbi.nih.gov/entrez/query.fcgi?db=gene&cmd=Retrieve&dopt=summary&list_uids=56829) | Hs.133512 | 12.2 | 9.62 | -2.58 | 0.448 | 0.002 | 3.864 | Homo sapiens zinc finger CCCH-type, antiviral 1 (ZC3HAV1), transcript variant 2, mRNA [NM_024625] |
| [A_32_P168727](http://arrays.ucsf.edu/cgi-bin/oligo_db.pl?oligo=A_32_P168727) |  | [NA](../SIX3/NA) |  | 12.31 | 9.73 | -2.58 | 0.16 | 0.001 | 4.864 |  |
| [A_23_P68155](http://arrays.ucsf.edu/cgi-bin/oligo_db.pl?oligo=A_23_P68155) | IFIH1 | [64135](http://www.ncbi.nih.gov/entrez/query.fcgi?db=gene&cmd=Retrieve&dopt=summary&list_uids=64135) | Hs.163173 | 14.29 | 11.7 | -2.59 | 0.055 | 0.001 | 5.871 | Homo sapiens interferon induced with helicase C domain 1 (IFIH1), mRNA [NM_022168] |
| [A_23_P315364](http://arrays.ucsf.edu/cgi-bin/oligo_db.pl?oligo=A_23_P315364) | CXCL2 | [2920](http://www.ncbi.nih.gov/entrez/query.fcgi?db=gene&cmd=Retrieve&dopt=summary&list_uids=2920) | Hs.590921 | 10.21 | 7.62 | -2.59 | 1 | 0.012 | 0.582 | Homo sapiens chemokine (C-X-C motif) ligand 2 (CXCL2), mRNA [NM_002089] |
| [A_32_P42705](http://arrays.ucsf.edu/cgi-bin/oligo_db.pl?oligo=A_32_P42705) |  | [NA](../SIX3/NA) | Hs.201854 | 10.28 | 7.58 | -2.69 | 0.864 | 0.003 | 3.217 | Homo sapiens, clone IMAGE:4429392, mRNA, partial cds. [BC017721] |
| [A_24_P304071](http://arrays.ucsf.edu/cgi-bin/oligo_db.pl?oligo=A_24_P304071) | IFIT2 | [3433](http://www.ncbi.nih.gov/entrez/query.fcgi?db=gene&cmd=Retrieve&dopt=summary&list_uids=3433) | Hs.437609 | 12.3 | 9.59 | -2.7 | 1 | 0.007 | 1.44 | Homo sapiens interferon-induced protein with tetratricopeptide repeats 2 (IFIT2), mRNA [NM_001547] |
| [A_23_P52207](http://arrays.ucsf.edu/cgi-bin/oligo_db.pl?oligo=A_23_P52207) | BAMBI | [25805](http://www.ncbi.nih.gov/entrez/query.fcgi?db=gene&cmd=Retrieve&dopt=summary&list_uids=25805) | Hs.533336 | 15.07 | 12.35 | -2.72 | 0.013 | 0 | 7.203 | Homo sapiens BMP and activin membrane-bound inhibitor homolog (Xenopus laevis) (BAMBI), mRNA [NM_012342] |
| [A_23_P152838](http://arrays.ucsf.edu/cgi-bin/oligo_db.pl?oligo=A_23_P152838) | CCL5 | [6352](http://www.ncbi.nih.gov/entrez/query.fcgi?db=gene&cmd=Retrieve&dopt=summary&list_uids=6352) | Hs.514821 | 14.36 | 11.6 | -2.75 | 1 | 0.006 | 1.814 | Homo sapiens chemokine (C-C motif) ligand 5 (CCL5), mRNA [NM_002985] |
| [A_23_P217866](http://arrays.ucsf.edu/cgi-bin/oligo_db.pl?oligo=A_23_P217866) | IFI16 | [3428](http://www.ncbi.nih.gov/entrez/query.fcgi?db=gene&cmd=Retrieve&dopt=summary&list_uids=3428) | Hs.380250 | 10.47 | 7.69 | -2.77 | 0.724 | 0.003 | 3.392 | Homo sapiens interferon, gamma-inducible protein 16 (IFI16), mRNA [NM_005531] |
| [A_23_P139786](http://arrays.ucsf.edu/cgi-bin/oligo_db.pl?oligo=A_23_P139786) | OASL | [8638](http://www.ncbi.nih.gov/entrez/query.fcgi?db=gene&cmd=Retrieve&dopt=summary&list_uids=8638) | Hs.118633 | 16.45 | 13.65 | -2.8 | 1 | 0.012 | 0.521 | Homo sapiens 2'-5'-oligoadenylate synthetase-like (OASL), transcript variant 1, mRNA [NM_003733] |
| [A_24_P250922](http://arrays.ucsf.edu/cgi-bin/oligo_db.pl?oligo=A_24_P250922) | PTGS2 | [5743](http://www.ncbi.nih.gov/entrez/query.fcgi?db=gene&cmd=Retrieve&dopt=summary&list_uids=5743) | Hs.196384 | 10.42 | 7.61 | -2.81 | 1 | 0.004 | 2.473 | Homo sapiens prostaglandin-endoperoxide synthase 2 (prostaglandin G/H synthase and cyclooxygenase) (PTGS2), mRNA [NM_000963] |
| [A_24_P254933](http://arrays.ucsf.edu/cgi-bin/oligo_db.pl?oligo=A_24_P254933) |  | [NA](../SIX3/NA) |  | 15.33 | 12.51 | -2.83 | 0.193 | 0.001 | 4.684 | interferon induced transmembrane protein 3 (1-8U) (IFITM3), mRNA [Source:RefSeq_dna;Acc:NM_021034] [ENST00000270031] |
| [A_24_P15502](http://arrays.ucsf.edu/cgi-bin/oligo_db.pl?oligo=A_24_P15502) |  | [NA](../SIX3/NA) |  | 15.02 | 12.19 | -2.83 | 0.043 | 0.001 | 6.115 |  |
| [A_32_P167592](http://arrays.ucsf.edu/cgi-bin/oligo_db.pl?oligo=A_32_P167592) |  | [NA](../SIX3/NA) | Hs.646882 | 16.15 | 13.3 | -2.85 | 0.108 | 0.001 | 5.237 | similar to Interferon-induced transmembrane protein 3 (Interferon-inducible protein 1-8U) (LOC650205), mRNA [Source:RefSeq_dna;Acc:XR_018421] [ENST00000339867] |
| [A_24_P77008](http://arrays.ucsf.edu/cgi-bin/oligo_db.pl?oligo=A_24_P77008) | PTGS2 | [5743](http://www.ncbi.nih.gov/entrez/query.fcgi?db=gene&cmd=Retrieve&dopt=summary&list_uids=5743) | Hs.196384 | 9.92 | 7.07 | -2.85 | 1 | 0.012 | 0.514 | Homo sapiens prostaglandin-endoperoxide synthase 2 (prostaglandin G/H synthase and cyclooxygenase) (PTGS2), mRNA [NM_000963] |
| [A_24_P16124](http://arrays.ucsf.edu/cgi-bin/oligo_db.pl?oligo=A_24_P16124) | IFITM4P | [340198](http://www.ncbi.nih.gov/entrez/query.fcgi?db=gene&cmd=Retrieve&dopt=summary&list_uids=340198) |  | 14.64 | 11.76 | -2.89 | 0.083 | 0.001 | 5.487 | Homo sapiens interferon induced transmembrane protein 4 pseudogene (IFITM4P) on chromosome 6 [NR_001590] |
| [A_23_P87545](http://arrays.ucsf.edu/cgi-bin/oligo_db.pl?oligo=A_23_P87545) | IFITM3 | [10410](http://www.ncbi.nih.gov/entrez/query.fcgi?db=gene&cmd=Retrieve&dopt=summary&list_uids=10410) | Hs.374650 | 16.67 | 13.7 | -2.97 | 0.031 | 0.001 | 6.399 | Homo sapiens interferon induced transmembrane protein 3 (1-8U) (IFITM3), mRNA [NM_021034] |
| [A_24_P941167](http://arrays.ucsf.edu/cgi-bin/oligo_db.pl?oligo=A_24_P941167) | APOL6 | [80830](http://www.ncbi.nih.gov/entrez/query.fcgi?db=gene&cmd=Retrieve&dopt=summary&list_uids=80830) | Hs.257352 | 10.11 | 7.13 | -2.98 | 1 | 0.005 | 2.195 | Homo sapiens apolipoprotein L, 6 (APOL6), mRNA [NM_030641] |
| [A_23_P310921](http://arrays.ucsf.edu/cgi-bin/oligo_db.pl?oligo=A_23_P310921) | PCDH7 | [5099](http://www.ncbi.nih.gov/entrez/query.fcgi?db=gene&cmd=Retrieve&dopt=summary&list_uids=5099) | Hs.570785 | 11.49 | 8.46 | -3.03 | 1 | 0.006 | 1.809 | Homo sapiens protocadherin 7 (PCDH7), transcript variant a, mRNA [NM_002589] |
| [A_23_P218442](http://arrays.ucsf.edu/cgi-bin/oligo_db.pl?oligo=A_23_P218442) | CEACAM6 | [4680](http://www.ncbi.nih.gov/entrez/query.fcgi?db=gene&cmd=Retrieve&dopt=summary&list_uids=4680) | Hs.466814 | 9.74 | 6.63 | -3.11 | 1 | 0.003 | 2.876 | Homo sapiens carcinoembryonic antigen-related cell adhesion molecule 6 (non-specific cross reacting antigen) (CEACAM6), mRNA [NM_002483] |
| [A_24_P408047](http://arrays.ucsf.edu/cgi-bin/oligo_db.pl?oligo=A_24_P408047) | PLEKHA4 | [57664](http://www.ncbi.nih.gov/entrez/query.fcgi?db=gene&cmd=Retrieve&dopt=summary&list_uids=57664) | Hs.9469 | 11.81 | 8.68 | -3.13 | 0.088 | 0.001 | 5.438 | Homo sapiens pleckstrin homology domain containing, family A (phosphoinositide binding specific) member 4 (PLEKHA4), mRNA [NM_020904] |
| [A_24_P7040](http://arrays.ucsf.edu/cgi-bin/oligo_db.pl?oligo=A_24_P7040) | LOC123862 | [123862](http://www.ncbi.nih.gov/entrez/query.fcgi?db=gene&cmd=Retrieve&dopt=summary&list_uids=123862) | Hs.647379 | 15.21 | 12.08 | -3.14 | 0.042 | 0.001 | 6.123 | PREDICTED: Homo sapiens similar to Interferon-induced transmembrane protein 3 (Interferon-inducible protein 1-8U) (LOC123862), mRNA [XR_018504] |
| [A_23_P69383](http://arrays.ucsf.edu/cgi-bin/oligo_db.pl?oligo=A_23_P69383) | PARP9 | [83666](http://www.ncbi.nih.gov/entrez/query.fcgi?db=gene&cmd=Retrieve&dopt=summary&list_uids=83666) | Hs.518200 | 11.9 | 8.76 | -3.15 | 0.172 | 0.001 | 4.793 | Homo sapiens poly (ADP-ribose) polymerase family, member 9 (PARP9), mRNA [NM_031458] |
| [A_24_P11506](http://arrays.ucsf.edu/cgi-bin/oligo_db.pl?oligo=A_24_P11506) | KYNU | [8942](http://www.ncbi.nih.gov/entrez/query.fcgi?db=gene&cmd=Retrieve&dopt=summary&list_uids=8942) | Hs.470126 | 11 | 7.79 | -3.21 | 1 | 0.004 | 2.772 | Homo sapiens kynureninase (L-kynurenine hydrolase) (KYNU), transcript variant 1, mRNA [NM_003937] |
| [A_23_P52266](http://arrays.ucsf.edu/cgi-bin/oligo_db.pl?oligo=A_23_P52266) | IFIT1 | [3434](http://www.ncbi.nih.gov/entrez/query.fcgi?db=gene&cmd=Retrieve&dopt=summary&list_uids=3434) | Hs.20315 | 16.99 | 13.77 | -3.22 | 0.727 | 0.003 | 3.387 | Homo sapiens interferon-induced protein with tetratricopeptide repeats 1 (IFIT1), transcript variant 2, mRNA [NM_001548] |
| [A_23_P23074](http://arrays.ucsf.edu/cgi-bin/oligo_db.pl?oligo=A_23_P23074) | IFI44 | [10561](http://www.ncbi.nih.gov/entrez/query.fcgi?db=gene&cmd=Retrieve&dopt=summary&list_uids=10561) | Hs.82316 | 14.72 | 11.49 | -3.23 | 0 | 0 | 10.591 | Homo sapiens interferon-induced protein 44 (IFI44), mRNA [NM_006417] |
| [A_23_P45871](http://arrays.ucsf.edu/cgi-bin/oligo_db.pl?oligo=A_23_P45871) | IFI44L | [10964](http://www.ncbi.nih.gov/entrez/query.fcgi?db=gene&cmd=Retrieve&dopt=summary&list_uids=10964) | Hs.389724 | 12.48 | 9.19 | -3.29 | 0.016 | 0 | 6.988 | Homo sapiens interferon-induced protein 44-like (IFI44L), mRNA [NM_006820] |
| [A_23_P59005](http://arrays.ucsf.edu/cgi-bin/oligo_db.pl?oligo=A_23_P59005) | TAP1 | [6890](http://www.ncbi.nih.gov/entrez/query.fcgi?db=gene&cmd=Retrieve&dopt=summary&list_uids=6890) | Hs.352018 | 12.99 | 9.69 | -3.3 | 0.083 | 0.001 | 5.487 | Homo sapiens transporter 1, ATP-binding cassette, sub-family B (MDR/TAP) (TAP1), mRNA [NM_000593] |
| [A_23_P56898](http://arrays.ucsf.edu/cgi-bin/oligo_db.pl?oligo=A_23_P56898) | KYNU | [8942](http://www.ncbi.nih.gov/entrez/query.fcgi?db=gene&cmd=Retrieve&dopt=summary&list_uids=8942) | Hs.470126 | 12.72 | 9.4 | -3.32 | 0.965 | 0.003 | 3.107 | Homo sapiens kynureninase (L-kynurenine hydrolase) (KYNU), transcript variant 1, mRNA [NM_003937] |
| [A_23_P420209](http://arrays.ucsf.edu/cgi-bin/oligo_db.pl?oligo=A_23_P420209) | GCNT3 | [9245](http://www.ncbi.nih.gov/entrez/query.fcgi?db=gene&cmd=Retrieve&dopt=summary&list_uids=9245) | Hs.194710 | 10.88 | 7.54 | -3.33 | 0.38 | 0.002 | 4.026 | Homo sapiens glucosaminyl (N-acetyl) transferase 3, mucin type (GCNT3), mRNA [NM_004751] |
| [A_23_P35412](http://arrays.ucsf.edu/cgi-bin/oligo_db.pl?oligo=A_23_P35412) | IFIT3 | [3437](http://www.ncbi.nih.gov/entrez/query.fcgi?db=gene&cmd=Retrieve&dopt=summary&list_uids=3437) | Hs.47338 | 13.97 | 10.61 | -3.36 | 0.022 | 0 | 6.705 | Homo sapiens interferon-induced protein with tetratricopeptide repeats 3 (IFIT3), mRNA [NM_001549] |
| [A_24_P868905](http://arrays.ucsf.edu/cgi-bin/oligo_db.pl?oligo=A_24_P868905) | LOC391020 | [391020](http://www.ncbi.nih.gov/entrez/query.fcgi?db=gene&cmd=Retrieve&dopt=summary&list_uids=391020) | Hs.647618 | 14.53 | 10.95 | -3.58 | 0.001 | 0 | 9.662 | PREDICTED: Homo sapiens similar to Interferon-induced transmembrane protein 3 (Interferon-inducible protein 1-8U) (LOC391020), mRNA [XR_018907] |
| [A_23_P421483](http://arrays.ucsf.edu/cgi-bin/oligo_db.pl?oligo=A_23_P421483) | CEACAM6 | [4680](http://www.ncbi.nih.gov/entrez/query.fcgi?db=gene&cmd=Retrieve&dopt=summary&list_uids=4680) | Hs.466814 | 11.1 | 7.33 | -3.77 | 1 | 0.004 | 2.684 | Homo sapiens carcinoembryonic antigen-related cell adhesion molecule 6 (non-specific cross reacting antigen), mRNA (cDNA clone MGC:10467 IMAGE:3640231), complete cds. [BC005008] |
| [A_23_P201459](http://arrays.ucsf.edu/cgi-bin/oligo_db.pl?oligo=A_23_P201459) | IFI6 | [2537](http://www.ncbi.nih.gov/entrez/query.fcgi?db=gene&cmd=Retrieve&dopt=summary&list_uids=2537) | Hs.523847 | 13.96 | 10.14 | -3.82 | 0.653 | 0.002 | 3.494 | Homo sapiens interferon, alpha-inducible protein 6 (IFI6), transcript variant 3, mRNA [NM_022873] |
| [A_23_P151915](http://arrays.ucsf.edu/cgi-bin/oligo_db.pl?oligo=A_23_P151915) | GCNT3 | [9245](http://www.ncbi.nih.gov/entrez/query.fcgi?db=gene&cmd=Retrieve&dopt=summary&list_uids=9245) | Hs.194710 | 14.67 | 10.83 | -3.84 | 1 | 0.003 | 2.918 | glucosaminyl (N-acetyl) transferase 3, mucin type [Source:RefSeq_peptide;Acc:NP_004742] [ENST00000267857] |
| [A_23_P64828](http://arrays.ucsf.edu/cgi-bin/oligo_db.pl?oligo=A_23_P64828) | OAS1 | [4938](http://www.ncbi.nih.gov/entrez/query.fcgi?db=gene&cmd=Retrieve&dopt=summary&list_uids=4938) | Hs.524760 | 11.07 | 7.11 | -3.96 | 1 | 0.004 | 2.777 | Homo sapiens 2',5'-oligoadenylate synthetase 1, 40/46kDa (OAS1), transcript variant 2, mRNA [NM_002534] |
| [A_23_P153301](http://arrays.ucsf.edu/cgi-bin/oligo_db.pl?oligo=A_23_P153301) | CEACAM5 | [1048](http://www.ncbi.nih.gov/entrez/query.fcgi?db=gene&cmd=Retrieve&dopt=summary&list_uids=1048) | Hs.466814 | 11.2 | 6.93 | -4.27 | 0.427 | 0.002 | 3.912 | Homo sapiens carcinoembryonic antigen-related cell adhesion molecule 5 (CEACAM5), mRNA [NM_004363] |
| [A_24_P270460](http://arrays.ucsf.edu/cgi-bin/oligo_db.pl?oligo=A_24_P270460) | IFI27 | [3429](http://www.ncbi.nih.gov/entrez/query.fcgi?db=gene&cmd=Retrieve&dopt=summary&list_uids=3429) | Hs.532634 | 12.69 | 8.1 | -4.59 | 0.703 | 0.003 | 3.421 | Homo sapiens interferon, alpha-inducible protein 27 (IFI27), mRNA [NM_005532] |
| [A_23_P48513](http://arrays.ucsf.edu/cgi-bin/oligo_db.pl?oligo=A_23_P48513) | IFI27 | [3429](http://www.ncbi.nih.gov/entrez/query.fcgi?db=gene&cmd=Retrieve&dopt=summary&list_uids=3429) | Hs.532634 | 13.35 | 8.68 | -4.66 | 0.978 | 0.003 | 3.094 | Homo sapiens interferon, alpha-inducible protein 27 (IFI27), mRNA [NM_005532] |

Note:

– Listed genes were selected by B>0 & >2-fold down-regulation.

M – log_2_ (S2/S1). Log 2 based fold change of entity of interest. M=1 means two-fold increase in S2 compared to S1. M=0 means equal expression. M= -1 means 2 fold down-regulation.

aveA – average log_2_ based intensity of the same probe across all arrays, a proxy for gene expression level.

B - log posterior odds ratios, ratio between the probability that a given gene is differentially expressed (DE) over the probability that a given gene is not differentially expressed; B≥0 means equal or more probability that a gene is DE than non-DE.

FDR – False Discovery Rate, which is the percentage of falsely declared DE genes among the set of declared DE genes. A FDR cutoff of 0.01 indicates that 1% of the declared DE genes are expected to false positives.

AdjP – Adjusted p-value, which controls for family-wise error rates, the probability of having more than one false discovery. An adjusted p-value cutoff of 0.01 indicates that the declared DE set has 1% chance to have one false positive.
