# Supplementary material for: Down-Regulation of SIX3 is Associated with Clinical Outcome in Lung Adenocarcinoma
Source: PLoS One. 2013 Aug 16;8(8):e71816. doi: 10.1371/journal.pone.0071816 (PMC3745425; doi:10.1371/journal.pone.0071816)
Supplement: Table S2 — qPCR Analysis of SIX3 Expression in Tissue Samples of NSCLC Patients. (DOC) [file pone.0071816.s002.doc]

| Table S2. qPCR Analysis of SIX3 Expression in Tissue Samples of NSCLC Patients | | |
| --- | --- | --- |
| Case Number | Pathology | Relative SIX3 Expression (calculated by 2^-dCt^ x 100 method) |
| 358 | Lung-Squamous Cell | 0.02805840 |
| 506 | Lung-Adenocarcinoma | 0.02888736 |
| 497 | Lung-Adenocarcinoma | 0.03659221 |
| 9801 | Lung-BAC features | 0.04641511 |
| 354 | Lung-Squamous Cell | 0.04757550 |
| 9705 | Lung-BAC features | 0.05061401 |
| 294 | Lung-BAC features | 0.05164378 |
| 422 | Lung-Squamous Cell | 0.05305240 |
| 238 | Lung-Adenocarcinoma | 0.06916294 |
| 153 | Lung-Adenocarcinoma | 0.07028565 |
| 496 | Lung-Adenocarcinoma | 0.07774597 |
| 614 | Lung-Adenocarcinoma | 0.08075160 |
| 565 | Lung-BAC features | 0.11194519 |
| 483 | Lung-Adenocarcinoma | 0.12037457 |
| 224 | Lung-BAC features | 0.13527955 |
| 525 | Lung-BAC features | 0.15725068 |
| 504 | Lung-Adenocarcinoma | 0.17206697 |
| 508 | Lung-Adenocarcinoma | 0.17776038 |
| 312 | Lung-Adenocarcinoma | 0.19315448 |
| 520 | Lung-Adenocarcinoma | 0.23799448 |
| 9908 | Lung-Adenocarcinoma | 0.25268779 |
| 615 | Lung-Adenocarcinoma | 0.27437323 |
| 532 | Lung-Squamous Cell | 0.27658887 |
| 476 | Lung-BAC features | 0.29315725 |
| 291 | Lung-Adenocarcinoma | 0.30076389 |
| 478 | Lung-Adenocarcinoma | 0.31936445 |
| 284 | Lung-BAC features | 0.33669534 |
| 457 | Lung-Adenocarcinoma | 0.34362432 |
| 355 | Lung-BAC features | 0.36178856 |
| 9843 | Lung-Adenocarcinoma | 0.36534040 |
| 231 | Lung-Adenocarcinoma | 0.37861413 |
| 408 | Lung-BAC features | 0.38063784 |
| 444 | Lung-Adenocarcinoma | 0.38382966 |
| 345 | Lung-Adenocarcinoma | 0.39643448 |
| 356 | Lung-Adenocarcinoma | 0.40432861 |
| 628 | Lung-Adenocarcinoma | 0.42987125 |
| 511 | Lung-BAC features | 0.45033671 |
| 701 | Lung-Adenocarcinoma | 0.45220350 |
| 559 | Lung-BAC features | 0.51907220 |
| 308 | Lung-Adenocarcinoma | 0.52136202 |
| 621 | Lung-BAC features | 0.53601817 |
| 494 | Lung-Adenocarcinoma | 0.54033250 |
| 680 | Lung-Adenocarcinoma | 0.54774961 |
| 9834 | Lung-BAC features | 0.55007080 |
| 225 | Lung-Adenocarcinoma | 0.56661311 |
| 313 | Lung-BAC features | 0.56709543 |
| 316 | Lung-Adenocarcinoma | 0.58254956 |
| 516 | Lung-Adenocarcinoma | 0.59927670 |
| 380 | Lung-Squamous Cell | 0.60266571 |
| 9913 | Lung-Adenocarcinoma | 0.66105199 |
| 309 | Lung-BAC features | 0.67162095 |
| 420 | Lung-Adenocarcinoma | 0.70323187 |
| 535 | Lung-Adenocarcinoma | 0.70710800 |
| 427 | Lung-BAC features | 0.77252026 |
| 500 | Lung-Squamous Cell | 0.77307063 |
| 461 | Lung-Adenocarcinoma | 0.77533065 |
| 406 | Lung-Adenocarcinoma | 0.87703900 |
| 381 | Lung-BAC features | 0.88548329 |
| 433 | Lung-BAC features | 0.89264495 |
| 460 | Lung-Squamous Cell | 0.93426251 |
| 607 | Lung-BAC features | 0.94212510 |
| 230 | Lung-Squamous Cell | 0.95535906 |
| 489 | Lung-BAC features | 0.96226286 |
| 515 | Lung-Adenocarcinoma | 1.05206587 |
| 80 | Lung-Adenocarcinoma | 1.10447960 |
| 426 | Lung-BAC features | 1.13076009 |
| 378 | Lung-Adenocarcinoma | 1.13813334 |
| 586 | Lung-BAC features | 1.14517110 |
| 451 | Lung-Adenocarcinoma | 1.29917124 |
| 454 | Lung-Adenocarcinoma | 1.38775854 |
| 329 | Lung-Adenocarcinoma | 1.43684862 |
| 575 | Lung-Adenocarcinoma | 1.62679695 |
| 436 | Lung-Adenocarcinoma | 1.63606719 |
| 452 | Lung-Adenocarcinoma | 1.67348858 |
| 9803 | Lung-Adenocarcinoma | 1.67715087 |
| 333 | Lung-BAC features | 1.73567975 |
| 507 | Lung-Adenocarcinoma | 1.86368517 |
| 9855 | Lung-Adenocarcinoma | 1.86381238 |
| 411 | Lung-Squamous Cell | 1.89285817 |
| 269 | Lung-Squamous Cell | 1.90639233 |
| 304 | Lung-Adenocarcinoma | 1.96660885 |
| 310 | Lung-Adenocarcinoma | 2.00069882 |
| 919 | Lung-BAC features | 2.15324577 |
| 583 | Lung-Adenocarcinoma | 2.21549535 |
| 9854 | Lung-Adenocarcinoma | 2.26645818 |
| 556 | Lung-BAC features | 2.27547793 |
| 107 | Lung-Squamous Cell | 2.30421407 |
| 112 | Lung-BAC features | 2.37126937 |
| 377 | Lung-Adenocarcinoma | 2.41535057 |
| 513 | Lung-Adenocarcinoma | 2.41699471 |
| 471 | Lung-BAC features | 2.43816455 |
| 493 | Lung-Adenocarcinoma | 2.45623631 |
| 373 | Lung-BAC features | 2.54894590 |
| 626 | Lung-BAC features | 2.58269836 |
| 234 | Lung-Squamous Cell | 2.67589182 |
| 613 | Lung-BAC features | 2.71462123 |
| 435 | Lung-Squamous Cell | 2.88957438 |
| 449 | Lung-BAC features | 3.19648512 |
| 437 | Lung-Adenocarcinoma | 3.28362972 |
| 9739 | Lung-BAC features | 3.30555580 |
| 424 | Lung-Adenocarcinoma | 3.46739869 |
| 465 | Lung-BAC features | 3.48759646 |
| 344 | Lung-Adenocarcinoma | 3.57181090 |
| 417 | Lung-Adenocarcinoma | 3.67175788 |
| 113 | Lung-BAC features | 3.74332099 |
| 462 | Lung-Squamous Cell | 3.93094718 |
| 85 | Lung-Squamous Cell | 4.27805597 |
| 24 | Lung-BAC features | 4.34210211 |
| 279 | Lung-Adenocarcinoma | 4.50169442 |
| 235 | Lung-Adenocarcinoma | 4.85377635 |
| 139 | Lung-Adenocarcinoma | 4.94511560 |
| 9896 | Lung-Adenocarcinoma | 5.45455201 |
| 684 | Lung-BAC features | 6.21468740 |
| 527 | Lung-Adenocarcinoma | 6.40855902 |
| 9891 | Lung-BAC features | 6.77262542 |
| 130 | Lung-Adenocarcinoma | 6.95783337 |
| 442 | Lung-BAC features | 7.34654364 |
| 744 | Lung-Adenocarcinoma | 8.40116910 |
| 9864 | Lung-BAC features | 8.47733335 |
| 375 | Lung-Adenocarcinoma | 9.41942196 |
| 836 | Lung-Adenocarcinoma | 9.47478930 |
| 222 | Lung-BAC features | 10.88020592 |
| 963 | Lung-Adenocarcinoma | 10.97302125 |
| 711 | Lung-BAC features | 11.47327118 |
| 9890 | Lung-BAC features | 11.82786971 |
| 360 | Lung-BAC features | 12.01344546 |
| 66 | Lung-BAC features | 12.13292520 |
| 93 | Lung-BAC features | 12.17361497 |
| 146 | Lung-Adenocarcinoma | 12.21013307 |
| 386 | Lung-Adenocarcinoma | 12.48220284 |
| 404 | Lung-Adenocarcinoma | 14.27313618 |
| 327 | Lung-Adenocarcinoma | 14.41285166 |
| 658 | Lung-BAC features | 16.77325656 |
| 9844 | Lung-BAC features | 17.26885043 |
| 718 | Lung-BAC features | 17.75980702 |
| 866 | Lung-Adenocarcinoma | 18.47795800 |
| 102 | Lung-Adenocarcinoma | 19.51476919 |
| 774 | Lung-Adenocarcinoma | 20.49772365 |
| 927 | Lung-Adenocarcinoma | 23.63391157 |
| 854 | Lung-Adenocarcinoma | 27.24133210 |
| 526 | Lung-BAC features | 28.89512875 |
| 611 | Lung-BAC features | 30.99502266 |
| 90 | Lung-BAC features | 31.21854717 |
| 635 | Lung-BAC features | 31.64280193 |
| 470 | Lung-BAC features | 31.90413216 |
| 640 | Lung-BAC features | 38.44986741 |
| 26 | Lung-BAC features | 40.43423961 |
| 677 | Lung-BAC features | 40.70038528 |
| 748 | Lung-BAC features | 45.03987744 |
| 643 | Lung-Adenocarcinoma | 53.28378585 |
| 735 | Lung-BAC features | 71.44141587 |
| 730 | Lung-Adenocarcinoma | 73.82156903 |
| 219 | Lung-Adenocarcinoma | 123.21682723 |
| 353 | Lung-BAC features | 164.67873757 |
| 629 | Lung-BAC features | 220.35080546 |
| 338 | Lung-Adenocarcinoma | 305.94361795 |
| 295 | Lung-BAC features | 329.18254345 |
| 624 | Lung-BAC features | 405.24909299 |
| 364 | Lung-BAC features | 438.31867335 |
| 418 | Lung-Adenocarcinoma | 452.78530599 |
|  |  |  |
